# Protein O-Mannosylation in the Murine Brain: Occurrence of Mono-O-Mannosyl Glycans and Identification of New Substrates

**DOI:** 10.1371/journal.pone.0166119

**Published:** 2016-11-03

**Authors:** Markus F. Bartels, Patrick R. Winterhalter, Jin Yu, Yan Liu, Mark Lommel, Frank Möhrlen, Huaiyu Hu, Ten Feizi, Ulrika Westerlind, Thomas Ruppert, Sabine Strahl

**Affiliations:** 1 Centre for Organismal Studies, Department of Cell Chemistry, Heidelberg University, Heidelberg, Germany; 2 Center for Molecular Biology Heidelberg (ZMBH), Core Facility for Mass Spectrometry and Proteomics, Heidelberg University, Heidelberg, Germany; 3 Gesellschaft zur Förderung der Analytischen Wissenschaften e.V., ISAS–Leibniz Institute for Analytical Sciences, Dortmund, Germany; 4 Glycosciences Laboratory, Department of Medicine, Imperial College London, Hammersmith Campus, London, United Kingdom; 5 Centre for Organismal Studies, Department of Animal Molecular Physiology, Heidelberg University, Heidelberg, Germany; 6 Institute for Human Performance, Department of Neuroscience and Physiology, Upstate Medical University, New York, United States of America; Universidade de Sao Paulo, BRAZIL

## Abstract

Protein O-mannosylation is a post-translational modification essential for correct development of mammals. In humans, deficient O-mannosylation results in severe congenital muscular dystrophies often associated with impaired brain and eye development. Although various O-mannosylated proteins have been identified in the recent years, the distribution of O-mannosyl glycans in the mammalian brain and target proteins are still not well defined. In the present study, rabbit monoclonal antibodies directed against the O-mannosylated peptide YAT(α1-Man)AV were generated. Detailed characterization of clone RKU-1-3-5 revealed that this monoclonal antibody recognizes O-linked mannose also in different peptide and protein contexts. Using this tool, we observed that mono-O-mannosyl glycans occur ubiquitously throughout the murine brain but are especially enriched at inhibitory GABAergic neurons and at the perineural nets. Using a mass spectrometry-based approach, we further identified glycoproteins from the murine brain that bear single O-mannose residues. Among the candidates identified are members of the cadherin and plexin superfamilies and the perineural net protein neurocan. In addition, we identified neurexin 3, a cell adhesion protein involved in synaptic plasticity, and inter-alpha-trypsin inhibitor 5, a protease inhibitor important in stabilizing the extracellular matrix, as new O-mannosylated glycoproteins.

## Introduction

Protein O-mannosylation is an essential post-translational modification in fungi, animals and humans (reviewed in [1, 2]). In mammals, a heteromeric complex of protein O-mannosyltransferase 1 and 2 (POMT1, POMT2) transfers the initial mannose to serine and threonine residues of target proteins entering the endoplasmic reticulum (ER) using dolichol phosphate-activated mannose as carbohydrate donor [3]. The further modification of the protein-linked mannose includes its elongation in the Golgi apparatus. Linear core m1 (GlcNAcß1,2Man) and branched core m2 glycans ((GlcNAcß1,6)GlcNAcß1,2Man) are initiated by protein O-mannose β1,2-N-acetylglucosaminyltransferase 1 (POMGnT1) and β1,6-N-acetylglucosaminyltransferase (GnT-Vb/GnT-IX). These structures are further elongated by ß1,4-linked galactose and neuraminic acid derivatives [4, 5]. In addition, the synthesis of core m3 glycans is initiated by β1,4-N-acetylglucosaminyltransferase 2 (POMGnT2) in the ER. Core m3 glycans (GlcNAcß1,4Man) are elongated by β1,3-linked N-acetylgalactosamine and the addition of a phosphate group to the 6-position of the initial mannose. In the Golgi, the phosphate group is further modified by repetitive disaccharide units of [-3-xylose-α1,3-glucuronic acid-β1-]. This so called matriglycan is so far only found on α-dystroglycan (α-DG), which is the best investigated O-mannosylated protein today (reviewed in [6]).

In mice, absence of POMT1 or POMT2 results in embryonic lethality [7, 8]. Defective protein O-mannosylation in humans leads to a group of congenital muscular dystrophies referred to as α-dystroglycanopathies. Hallmarks are muscular dystrophy and severe malformations of the eye and brain [9, 10]. α-Dystroglycanopathies vary in severity, with the most fatal ones being the Walker-Warburg syndrome (WWS) and the muscle-eye-brain disease (MEB). In the brain, symptoms comprise neuronal over-migration leading to cobblestone (type II) lissencephaly and accompanying hydrocephalus in the cerebral cortex region, as well as drastic brain stem, pons and cerebellar hypoplasia [11–13]. Most of the clinical manifestations can be explained by a structurally impaired pial basement membrane, which allows for over-migration, subsequent perturbed placement of neurons past the *glia limitans*, and lamination defects [14, 15]. Causative for these defects is the insufficient glycosylation of α-DG, leading to impaired binding to extracellular matrix (ECM) proteins such as laminin, agrin or neurexin (reviewed in [6]). Notably, this binding requires the functional matriglycan, which can be stained for by the IIH6 antibody [16, 17]. It was suggested that the length of the matriglycan directly correlates with potential binding events and therefore a more tightly packed ECM [18]. Disease-relevant mouse models verified that O-mannosylation is of major importance during embryonic brain development, especially for the structural build-up of the pial basement membrane [19, 20].

Early structural analysis of O-glycans in rabbit brain indicated 30% of all O-linked glycans to be of the O-mannosyl type [21, 22]. Also more recent global O-glycomics approaches revealed significant levels of O-mannosyl glycans in the mouse brain [23]. But, α-DG bears only a minor amount of the total O-mannosyl glycans in the mammalian brain [24]. In the past years, the identification of further O-mannosylated proteins by high-throughput mass spectrometry (MS) -based methods was limited by technical and methodical constraints, such as the lack of suitable strategies for the enrichment of O-mannosyl glycoproteins or -peptides, handling the sample complexity of multiple and heterogeneously glycosylated peptides, or the instability of glycan modifications in MS fragmentation techniques. Thus, only a limited number of O-mannosylated proteins were identified from mouse or bovine brain, such as the cell surface glycoprotein CD24, the protein tyrosine phosphatase receptor-type ζ polypeptide 1 (RPTPζ, encoded by the PTPRZ1 gene), the cell adhesion protein neurofascin, and the ECM proteins versican and neurocan [25–30]. Recent advances in enrichment strategies of O-mannosyl glycopeptides followed by MS-based sequencing have paved the way for the identification of new O-mannosylated glycoproteins. Vester-Christensen and coworkers described the human O-mannose glycoproteome from glyco-engineered breast cancer cells where early glycan elongation steps in the Golgi apparatus were omitted to reduce the glycan complexity of the sample [31]. In combination with lectin weak affinity chromatography (LWAC) and advanced MS/MS techniques this so-called “SimpleCell” strategy resulted in the identification of cadherins and plexins as major targets of O-mannosylation. In order to discover O-mannosylated proteins directly from native tissues, we recently applied glycosidase treatment to generate glycopeptides bearing predominantly O-linked mannoses, and extracted those from complex peptide mixtures by the mannose-binding lectin concanavalin A (ConA). O-mannosylated peptides were then identified by combining the enzymatic specificity of α-mannosidase with quantitative liquid chromatography (LC)-MS/MS analyses after dimethyl labeling using stable isotopes [32]. This approach identified T- and E-cadherin from rabbit skeletal muscle and human embryonic kidney cells (HEK293), respectively, and revealed that in contrast to other known O-mannosylated proteins, predominantly single mannose residues are attached to these cell adhesion molecules [8, 32].

Despite the recent advances, the knowledge about the occurrence of O-mannosyl glycans in the mammalian brain is still limited. Here we took advantage of a new rabbit monoclonal antibody for detection of unsubstituted O-mannosyl glycans and characterized their distribution in the adult murine brain. Furthermore, using the recently established MS-based approaches, we identified new proteins from this organ which contain single O-linked mannose residues.

## Materials and Methods

### Animals

Mice used for this study were kept by the animal welfare officers of the University of Heidelberg. POMT2^f/f;Emx1-Cre+^ and POMT2^f/f;Emx1-Cre-^ mice were raised and sustained by the Institutional Animal Care and Use Committee of SUNY Upstate Medical University according to the guidelines of the National Institute of Health. Adult C57BL/6 (Black6) mice in between 8 and 24 weeks of age were sacrificed using isoflurane and freshly extracted brains were immediately processed for cryo-embedding. GAD^Cre^ mice were kindly provided by Dr. Hannah Monyer and Dr. Anne Herb. All experiments were in agreement to the directives and permits of the University of Heidelberg and the Animal Protection Law.

### Monoclonal antibody production and hybridoma culture

Rabbit monoclonal antibodies were produced in contract with Epitomics Inc (Woerdern, Austria). The immunogen KLH-C-Ahx-YAT[α1-Man]AV was synthesized, consisting of the keyhole limpet hemocyanin (KLH) as an immunostimulant, the O-mannosylated hexapeptide and the linker amino acid aminocaproic acid (Ahx). Four rabbits were immunized with the immunogen for five consecutive times and sera were evaluated using an established protocol from Epitomics for differential enzyme-linked immunosorbent assay (ELISA) using O-mannosylated or non-modified peptides. Sera were further evaluated for their specificity using enriched α-DG fractions from rabbit muscle as previously described [8]. One rabbit serum was tested positive, lymphocytes were extracted by splenectomy and fused with myeloma. 144 single hybridomas were screened by differential ELISA. A total of 38 clones specifically reacted with the mannosylated pentapeptide, and were further analyzed by Western blot using enriched α-DG fractions. In addition, reactivity against the recombinant proteins Nfasc186, hDGdel2 and hDG5 was tested (see below). Seven clones were identified to be exclusively directed against the mannosylated peptide. Another ten clones reacted with the O-mannosylated proteins as well. From these positive clones, RKU-1-3-5 was chosen for further propagation. Hybridoma cells were cultivated in RPMI1640 medium (Invitrogen/Life Technologies, Darmstadt, Germany), supplemented with Rabbit Hybridoma Supplement A (ab138912, Abcam, Cambridge, UK), fetal bovine serum (HYCLSH30080.03, VWR, Darmstadt, Germany), 1x HAT (21060–017, Invitrogen), 2 mM GlutaMAX-I (35050–061, Invitrogen) and 55 μM 2-mercaptoethanol (21985–023, Invitrogen). Antibody production was conducted according to Epitomics established protocols and suggestions. In brief, cells were adapted to serum-free medium consisting of IS MAB-CD medium (IS MAB-CD 91104, Irvine Scientific, Santa Ana, USA), supplemented with 4% (v/v) GlutaMAX (35050–061, Invitrogen) and 1x antibiotic-antimycotic (15240–062, Invitrogen), transferred to the cell compartment of a CELLine CL1000 incubator flask (Integra, Fernwald, Germany) and supplemented with 1 mg/ml BSA (A9578-50mL, Sigma-Aldrich, Munich, Germany). After cultivation for a total of 22 to 25 days, cell supernatants were collected, monoclonal IgG antibodies were enriched using Protein G columns and desalted with desalting columns following manufacturer’s instructions (GE Healthcare/Amersham, Munich, Germany and Epitomics).

### Cryosectioning and immunofluorescence analyses

Extracted brains were immediately embedded in tissue freezing medium (Jung) in embedding bowls (16848B; Polysciences, Eppelheim, Germany) on dry ice. 12 μm sections were mounted on Superfrost Plus carrier slides (H867.1; Carl Roth, Karlsruhe, Germany) and stored at—20°C. Sections were thawed for 30 min prior to fixation with 2% (wt/vol) paraformaldehyde in PBS for 20 min. After washing 3 times with 0.5x Tris-buffered saline (0.5x TBS), sections were blocked with 5% (vol/vol) Chemiblocker (2170-S; Chemicon/Merck Chemicals, Darmstadt, Germany) in 0.5x TBS containing 0.05% (vol/vol) Tween-20 (0.5x TBS/T) for 1 h. Primary antibodies were applied overnight in 0.5x TBS/T containing 5% (vol/vol) Chemiblocker in a humid chamber at 4°C. Secondary antibodies were incubated for 1 h diluted in 0.5x TBS/T containing 5% (vol/vol) Chemiblocker. Nuclei were counterstained with 0.3 μM DAPI for 3 min and sections were mounted in AquaPolyMount (Polysciences). For a list of used antibodies please refer to S1 Table (Santa Cruz, Heidelberg, Germany; R&D systems, Minneapolis, USA; Vector Laboratories, Burlingame, USA).

If necessary, antibody solutions were pre-incubated with 150 μM and 190 μM of O-mannosylated YATAV or non-modified peptide on a nutator for 24 h prior to application to the staining protocol above. Antibodies and peptides were diluted in 0.5x TBS/T containing 5% Chemiblocker.

For co-localization of neurocan with the epitopes recognized by the α-O-Man antibody, sections were detected in a sequential manner. After thawing, fixing and blocking the sections as described above, the primary neurocan antibody was applied in 5% Chemiblocker in 0.5x TBS/T for 1h. The secondary α-sheepCy3 antibody was applied thereafter diluted in 0.5x TBS/T containing 5% Chemiblocker for 1 h. Subsequently, sections were incubated with the primary α-O-Man antibody diluted in 0.5x TBS/T containing 5% Chemiblocker overnight at 4°C in a humid chamber and the staining protocol described above was further utilized. All other double stainings were carried out using the standard staining protocol and applying both primary and secondary antibodies together.

Detection of mouse antigens on mouse tissue was essentially performed as described in Beedle *et al*. 2012 [33]. Briefly, sections were thawed and fixed as described above and incubated with 0.1 M glycine/PBS for 10 min and 0.05% SDS/PBS for 30 min at 50°C. After blocking with 5% (vol/vol) goat serum (Jackson Immuno Research, West Grove, USA) in PBS for 1 h, endogenous IgG antibodies were blocked with 0.1 mg/ml PBS mouse F(ab)-fragment (Jackson ImmunoResearch) for 1 to 2 h and primary antibodies diluted in 5% goat serum in PBS were applied overnight in a humid chamber. Nuclei were stained and sections were mounted as described above.

For removal of N-linked glycans, murine brain cryosections were thawed, fixed with 4% (w/v) Paraformaldehyde in PBS for 20 min at RT and washed 3 times in 0.5x TBS. Glycan trimming was performed in the recommended buffer at 37°C for 3 h using 500 U of PNGase F (P0704L, New England Biolabs (NEB), Frankfurt am Main, Germany). After 5 washing steps with 0.5x TBS, antibody or lectin detection was continued as stated.

All immunostained images were acquired with a confocal Nikon 90i microscope. Pictures were analyzed using Adobe Photoshop CS4, wherein tonal values, contrast and gradation curves were optimized in comparable manners.

### Recombinant proteins

Recombinant proteins were produced and purified as described in Pacharra *et al*. 2012 for recombinant NFASC186 and in Breloy *et al*. 2008 for recombinant hDGdel2 and hDG5 [27, 34].

### Western blot analysis

Recombinant proteins (~ 0.4 μg each) were separated on 12% SDS-PA gels and transferred to nitrocellulose, as described in Sambrook *et al*. 2001 [35]. Blots were incubated with rabbit α-O-Man (1:25) or mouse α-pentaHis (Qiagen, Hilden, Germany; 1:2500) antibodies and the respective HRP-conjugated secondary antibodies (Sigma-Aldrich; 1:5000). Detection was performed by using ECL solutions (Amersham) and ImageQuant LAS500 (GE lifesciences).

### Glycopeptide enrichment

Sample preparation was done as described [31] with minor modifications: Mouse brain of a 8–12 weeks old mouse (~0.4 g) was pulverized in liquid nitrogen and dissolved in 4 ml lysis buffer (1% sodium deoxycholate (SDC), 10 mM tris(2-carboxyethyl)phosphine, 40 mM 2-chloroacetamide, 50 mM triethylammonium bicarbonate pH 8.5), heat inactivated at 90°C for 15 min and sonified (output control 6, duty cycle 60%) for 30 min. After centrifugation (1,000 × rcf for 10 min) the supernatant was incubated with TrypZean trypsin (1.6 mg, 1:250, Sigma-Aldrich) dissolved in 160 μl 0.01% trifluoroacetic acid (TFA) at 37°C overnight (16 h). Trypsin was heat inactivated (90°C, 30 min) and N-glycans were removed with PNGase F (4 μl, 37°C, 6 h, NEB). The sample was acidified with 1% TFA to precipitate SDC. After centrifugation (4000 × rcf for 5 min) peptides were desalted with two C18 Sep-Paks (500 mg, Waters, Milford, USA) and dried by SpeedVac overnight. For ConA separation the peptides were dissolved in 1 ml freshly prepared ConA buffer (10 mM Tris/HCl, pH 7.4, 150 mM NaCl, 1 mM CaCl_2_, 1 mM MgCl_2_, 1 mM MnCl_2_, 1 mM ZnCl_2_, 0.5 M urea) and loaded on a preequilibrated ConA lectin agarose column (3.5 m x 1 mm). The column was washed with 20 ml (~10 CVs) ConA buffer (100 μL/min) and peptides were eluted with ConA buffer containing 0.5 M α-D-Glucose (10 ml, 100 μL/min). The wash fractions (2 ml after the main flow-through passed) and the elution fractions were desalted by a C18 Sep-Pak column (100 mg, Waters), dried by SpeedVac and resuspended in a total volume of 30 μl of 0.1% TFA.

### Liquid chromatography and mass spectrometry

Peptides were subjected to dimethyl labeling treatment as described previously [32], or alternatively directly analyzed by subsequent trapping (UltiMate 3000 nanoRSLC; 5 μm Acclaim PepMap100 300 μm × 5 mm, Thermo Scientific, Schwerte, Germany) at a flow rate of 30 μl/min (1% acetonitrile (ACN), 1% formic acid (FA)). After 3 min peptides were separated on an analytical column (2 μm Acclaim PepMap RSLC 75 μm × 25 cm, Thermo Scientific) using a gradient of solution A (1% ACN, 5% DMSO, 0.1% FA) and B (90% ACN, 5% DMSO, 0.1% FA): 3% to 40% B in 110 min; 40% to 90% B in 10 min.; flow rate: 300 nl/min. Full-scan mass spectra (LTQ Orbitrap Elite MS (Thermo Scientific)) were acquired in the positive ion mode at 30000 resolution, followed by higher-energy collisional dissociation (HCD) fragmentation of the twenty most intense ions in the profile mode at 15000 resolution.

### Data processing

The MS data were analyzed as described [32] with minor modifications: Analysis was done with standard settings except noted otherwise using MaxQuant version 1.5.1.2 [36] and Andromeda search engine [37]. The UniProt mouse database was used (downloaded 01/16/2015, database entries: 83101). Carbamidomethylation of cysteine was set as a fixed modification. Methionine oxidation, asparagine/glutamine deamidation, protein N-terminus acetylation and serine/threonine hexosylation were set as variable modifications. For quantitation light and medium dimethyl label were added for the protein N-terminus and lysine. A minimal peptide length of 7 amino acids and a maximum of 2 miscleavages were allowed. The minimal score and deltascore for modified peptides were set to the default values 40 and 8, respectively. The maximum peptide spectrum match, proteins and site decoy fraction (FDR) were set to 0.01. ‘‘Re-quantify” and ‘‘Match between runs (2 min)” was activated. Bioinformatic data analysis was done with Perseus. In addition all the fragment spectra of modified peptides were hand annotated. Extracted ion chromatograms were done using the Qual browser software (Thermo Scientific) with the calculated peptide mass and a tolerance of 10 ppm.

## Results

### RKU-1-3-5 rabbit monoclonal antibody detects mono-O-mannosyl glycans in all regions of the mouse brain

**Fig 1:**
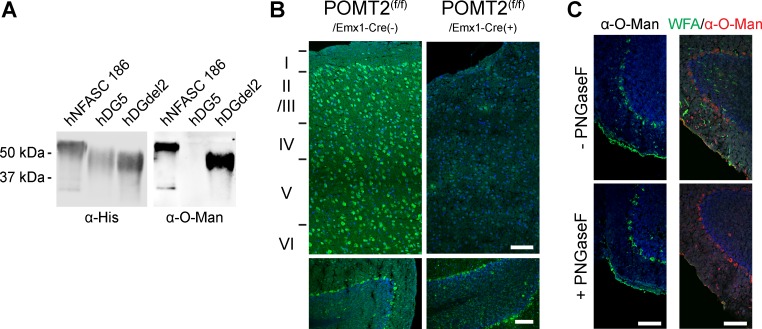
Specificity of the α-O-Man monoclonal antibody. A) Western blot analysis of recombinant His-tagged proteins [27, 34]. The α-O-Man antibody only detected O-mannosylated proteins (hNFASC186 and hDGdel2), whereas non-modified hDG5 was not recognized. B) Immunofluorescent staining with α-O-Man was drastically reduced in the cerebral cortex of POMT2^f/f;Emx1-Cre+^ mice (upper panel). Cerebral cortex layers are indicated on the left (I to VI). In contrast, staining of the Purkinje cell layer was comparable in the cerebellum, where cre is not expressed (lower panels). C) Cryosections of wild-type mouse brain were treated with PNGase F to remove all types of N-glycans. Efficient elimination is demonstrated by *Wisteria floribunda* agglutinin (WFA) staining, a marker of perineural nets [38] that is reactive to complex type N-glycans [39, 40]. No reduction in α-O-Man antibody signal upon N-glycan removal was observed. B,C) Nuclei were counterstained with DAPI, sections were cut sagittally. Scale bars = 50 μm.

We aimed to decipher the expression / distribution of protein O-mannosyl glycans in the mouse brain. Therefore, rabbit monoclonal antibody directed against the peptide YAT[α1-Man]AV, which does not react with the unglycosylated peptide YATAV, were established as detailed in Materials and Methods. 38 selected hybridoma clones were further evaluated for their ability to detect recombinant variants of human α-DG (hDG5 and hDGdel2) and human neurofascin 186 purified from HEK293T cells. For these proteins distinct O-mannosylation patterns have been demonstrated previously by mass spectrometry. On α-DG variant hDG5 no O-mannosyl glycans have been identified whereas on the hDGdel2 variant and on neurofascin 186 different types of O-mannosyl glycans, including unextended mannose residues, have been shown [27, 34]. In Western blots the monoclonal antibody RKU-1-3-5 (hereafter referred to as α-O-Man) recognized human neurofascin 186 and hDGdel2, but not the α-DG variant hDG5 (Fig 1A), further indicating its specificity. Glycopeptide array analysis demonstrated that the α-O-Man antibody reacts with Thr-coupled mannose in various peptide environments although to differing degrees (S1 Fig). Within the same peptides, elongated linear O-mannosyl glycans of the structures GlcNAcß1,2Man and Galß1,4GlcNAcß1,2Man as well as clustered mannoses and clustered GlcNAcß1,2Man glycans were not recognized (S1 Fig). Furthermore, to examine the specificity of α-O-Man towards diverse glycan sequences, it was analyzed using a neoglycolipid (NGL)-based oligosaccharide microarray of 492 oligosaccharide probes (S2 Fig). The repertoire of the probes encompasses a variety of mammalian type sequences, representative of N-glycans (high-mannose-type and neutral and sialylated complex-type); peripheral regions of O-glycans; blood group antigen-related sequences (A, B, H, Lewis^a^, Lewis^b^, Lewis^x^, and Lewis^y^) on linear or branched backbones and their sialylated and/or sulphated analogues; linear and branched poly-N-acetyllactosamine sequences; gangliosides; oligosaccharide fragments of glycosaminoglycans and polysialic acid. The array also included microbial and plant-derived homo-oligomers of glucose and of other monosaccharides. Screening analysis using the NGL-based microarray revealed binding of α-O-Man to both serine and threonine-linked mannose with a preference to the former (S2 Fig). Under the assay conditions, some cross reactivities were found of a number of mannose-terminating sequences, e.g. short α-linked mannose sequences, small oligomannose N-glycans (found in relatively large amounts in invertebrates and plants but not in mammals) and β1-4-linked oligomannose glycans (derived from plant polysaccharides and absent in mammals). To further investigate the binding of α-O-Man to oligomannose/high-mannose type N-glycans, the antibody was tested at different dilutions (S2 Table; S3 Fig), and the results suggest that at higher dilutions the antibody is more specific to α1-O-mannose amino acid probes. When coupled to a peptide backbone, no cross reactivities towards the core N-glycan structure Man_3_GlcNAc_2_ was detected (S4 Fig).

**Fig 2:**
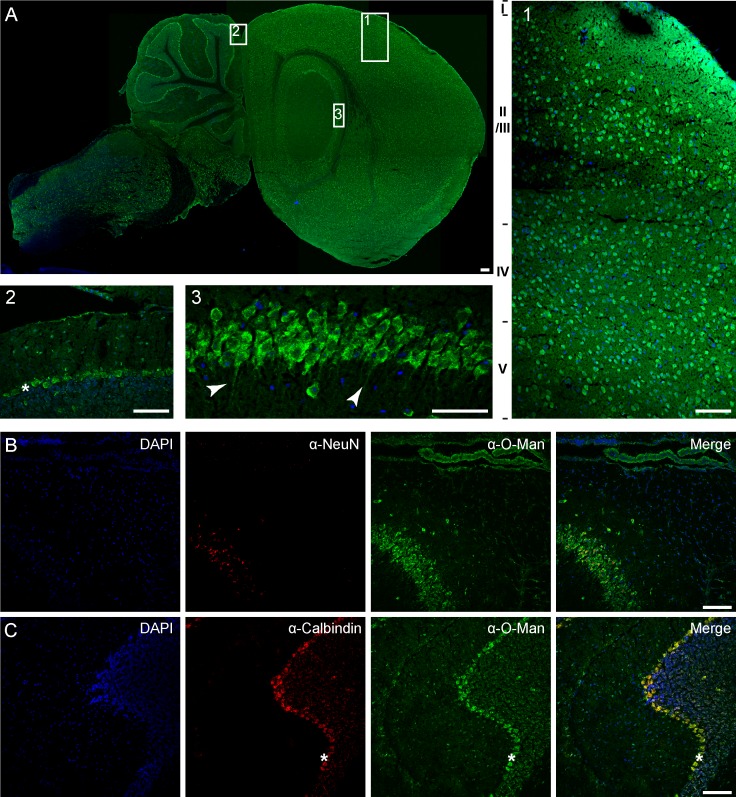
Mono-O-mannosyl glycans localize to distinct cell types throughout the murine brain. Sagittal section of WT murine brain counterstained with DAPI for nuclei labeling. A) Broad staining was achieved as shown in the overview (scale bar = 100 μm) and at higher magnifications in the cerebral cortex (1), the cerebellum (2), and the hippocampus (3). In more detail, staining in the cerebellum included single cells of the molecular layer, cells of the granular cell layer and cells of the Purkinje cell layer (indicated by asterisks). In the hippocampus, cells of the *cornu ammonis* region were labeled including their neuronal projections as indicated by arrowheads (picture 3 shows cells of the CA2 field). Individual cells of regions II to V of the cerebral cortex were stained by the α-O-Man antibody. Co-localization of mono-O-mannosyl glycans with neuronal cell marker (NeuN/Fox3) in the hippocampus (B) or with Purkinje cell marker (Calbindin) in the cerebellum (C) showing single channel signal and merged channels. NeuN-labeled hippocampal neurons of the *cornu ammonis* 2 (CA2) region were stained by the α-O-Man antibody. Purkinje cell localization of mono-O-mannosyl glycans was demonstrated by co-localization with Calbindin. Scale bar = 50 μm.

For further evaluation of clone RKU-1-3-5, we performed immunostaining of brain cross sections of adult wild-type mice. The α-O-Man antibody showed significant reactivity throughout the entire brain (Fig 2A). Immunoreactivity was completely lost when the α-O-Man antibody was pre-adsorbed with the O-mannosylated peptide (YAT[α1-Man]AV) whilst the unmodified peptide (YATAV) did not alter the staining intensity or pattern (S5A Fig). In addition, we took advantage of transgenic mice with a brain-specific knockout of POMT2 to further investigate the specificity of the α-O-Man monoclonal antibody. In POMT2^f/f;Emx1-Cre+^ mice O-mannosylation is abrogated in the neurons of the neocortex and the hippocampus, and in the glial cells of the pallium, whereas the cerebellum is not affected [20]. In agreement, staining of the Purkinje cell layer of the cerebellum (see below) of POMT2^f/f;Emx1-Cre+^ and control mice showed similar α-O-Man staining patterns (Fig 1B, lower panel). As shown in Fig 1B (upper panel), immunoreactivity of the α-O-Man antibody was almost absent in the cerebral cortex of POMT2^f/f;Emx1-Cre+^ mice as compared to wild-type littermates. Removal of N-glycans by PNGase F did not interfere with α-O-Man antibody binding (Fig 1C), substantiating the specificity of the immunostaining on cryosections. In addition, when O-mannosyl glycan biosynthesis was blocked by a POMT-specific inhibitor [8] in Madin-Darby canine kidney epithelial cells α-O-Man staining was omitted (S5B Fig), further demonstrating that the new α-O-Man monoclonal antibody is well suited for detection of mono-O-mannosyl glycans *in situ*.

### Mono-O-mannosyl glycans are particularly concentrated in inhibitory GABAergic neurons

α-O-Man immunostaining revealed mono-O-mannosyl glycans in all regions of the adult wild-type mouse brain (Fig 2A). Concentrated staining of neuronal cell bodies of the layers II to VI of the cerebral cortex (Fig 2A, panel 1), and single cells in the molecular cell layer, as well as the Purkinje cell and the granular cell layers of the cerebellum (Fig 2A, panel 2) was observed. Further, staining of pyramidal cells of the *cornu ammonis* (CA), including the projecting neurons, and of granular cells of the *dentate gyrus* (DG) in the hippocampus was pronounced (Fig 2A, panel 3). To better illustrate the staining profile in the hippocampus, α-O-Man antibody staining was compared to that of the neuronal marker NeuN/Fox3 [41]. We observed overlapping cell labeling of the CA field, but also identified additional cells in the areas surrounding the CA region (Fig 2B). In the cerebellum, α-O-Man staining of Purkinje cells was verified by Calbindin, a marker for Purkinje cells and their processes in cerebellar nerve tracts [42]. As shown in Fig 2C, in addition to Purkinje cells (indicated by asterisks) single cells from the molecular layer and, to some extent, granule cells were labeled by the α-O-Man antibody.

**Fig 3:**
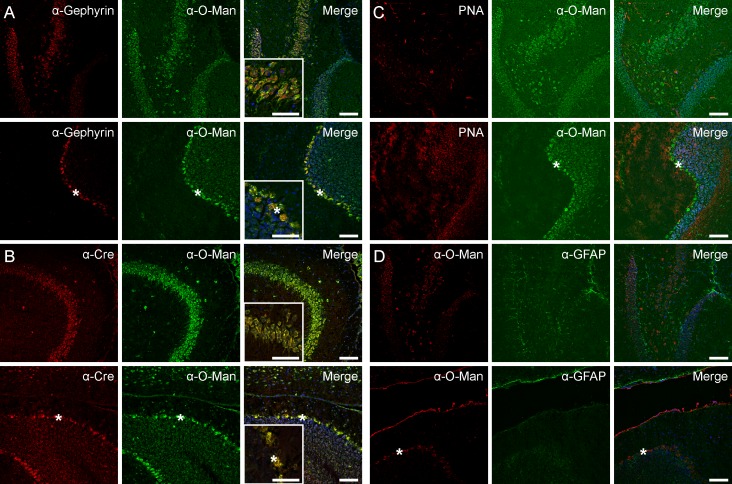
Mono-O-mannosyl glycan staining is pronounced in inhibitory neurons. Single channel images and merged channels including counterstained nuclei by DAPI (sagittal sections) are shown. A) Co-staining of gephyrin, an inhibitory synapse protein of GABAergic neurons and the α-O-Man antibody on WT murine brain cryosections of the hippocampus (first row) and cerebellum (second row). B) Staining of cryosections from GAD^Cre^ mice, expressing the cre protein specifically in GABAergic neurons. Cre-labeled GABAergic neurons were positive for α-O-Man antibody staining in the hippocampus (third row) and cerebellum (fourth row). Inlay pictures in A and B, as well as C and D show higher magnification pictures. Asterisks indicate the Purkinje cell layer. C) Peanut agglutinin (PNA), a marker for myelinated axons, did not co-localize with the signal of the α-O-Man antibody in the hippocampus (first row) or the cerebellum (second row). D) Glial marker glial fibrillary acidic protein (GFAP) did not overlap with staining of the α-O-Man antibody. Scale bars = 50 μm.

Our data indicated that GABAergic neurons (e.g., Purkinje cells), are preferentially stained by the α-O-Man antibody. To further verify this issue, we compared the pattern obtained by the α-O-Man antibody to gephyrin, a known marker for inhibitory GABAergic neurons [43]. As shown in Fig 3A, the staining overlapped to a high extent in the hippocampus (upper panels) and cerebellum (lower panels), although additional α-O-Man epitopes were detected for example in the granular cell layer (see also inlay in lower panel 3). Moreover, in brains from transgenic mice expressing the cre protein in GABAergic neurons [44] perfect co-localization of α-O-Man immunoreactivity with cre-positve neurons was observed. (Fig 3B), further corroborating the pronounced occurrence of mono-O-mannosyl glycans in GABAergic inhibitory neurons. In contrast, no matching or overlapping staining patterns of α-O-Man and peanut agglutinin (PNA), a marker for myelinated axons and white matter (Fig 3C), as well as the glial cell marker glial fibrillary acidic protein (GFAP; Fig 3D) were observed.

**Fig 4:**
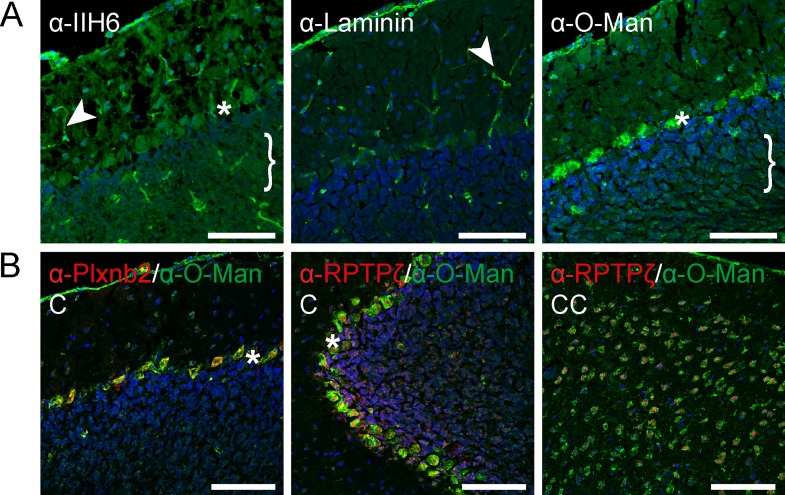
Known O-mannosylated proteins are located to the cell types identified by the α-O-Man antibody. A) α-DG as indicated by the IIH6 antibody reactive to matriglycan, and its interacting partner laminin preferentially stained vasculature (arrowhead) and the *glia limitans*. Purkinje cell layer (asterisks) and granular cell layer (bracket) were stained by the anti-laminin, the IIH6 and α-O-Man antibodies. IIH6 staining was performed following the protocol of Beedle *et al*. 2012 to detect mouse antigens on mouse tissue [33]. B) Plexin-B2 (Plxnb2) and RPTPζ showed comparable signal distribution as the α-O-Man antibody. Plxnb2 signals were located exclusively to the Purkinje cell layer (asterisks), whereas RPTPζ was primarily located to the granular cell layer in the cerebellum (abbreviated by C). In the cerebral cortex (CC), RPTPζ labeled single cells all of which were also stained for by the α-O-Man antibody. Nuclei were stained by DAPI, sagittal cryosections of WT mouse brains were used. Scale bars = 50 μm.

Next, we compared staining patterns of α-O-Man with previously described O-mannosylation target proteins. The most intensive studied glycoprotein carrying O-mannosyl glycans is α-DG. Localization of α-DG was assessed by the monoclonal antibody clone IIH6, which is known to bind to a carbohydrate-moiety that mediates the interaction between α-DG and laminin [16, 17]. Staining of sequential brain sections of wild-type mice showed comparable signals as illustrated exemplarily for the cerebellum (Fig 4A and S5C Fig and data not shown). As shown in Fig 4A, α-DG was detected in Purkinje cell and granular cell layers. In addition, prominent staining of vasculature was observed for IIH6 and laminin-reactive antibodies, but not for α-O-Man suggesting that the α-O-Man-directed epitope is not accessible in these structures. Furthermore, Plexin B2, an O-mannosylated transmembrane receptor involved in axon guidance and cell migration [45], co-localized with the α-O-Man signal of the Purkinje cell layer, but was absent in granular and molecular cell layers (Fig 4B, left panel). Also, the O-mannosylated glycoprotein RPTPζ ([25, 30]; see also below) could be co-localized with the mono-O-mannosyl glycan staining in the Purkinje cell layer (Fig 4B, middle panel) and in the cerebral cortex (Fig 4B, right panel).

### Mono-O-mannosyl glycans are localized to perineural nets

**Fig 5:**
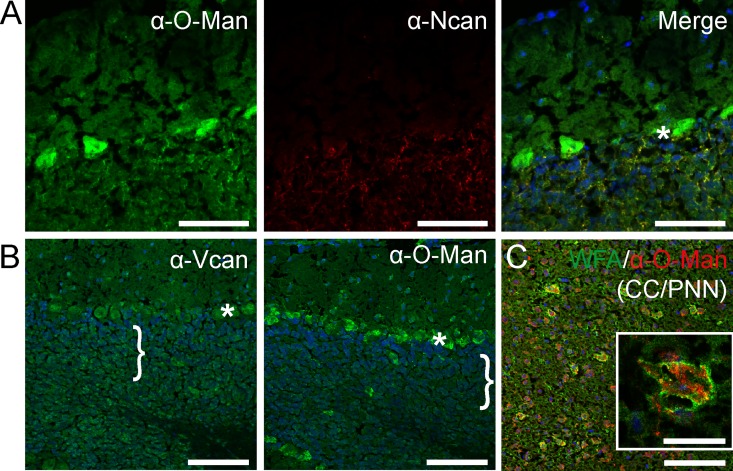
Mono-O-mannosyl glycans are concentrated in perineural nets. Sagittal cryosections were counterstained with DAPI and stained with the indicated antibody or lectin. A) Perineural net protein neurocan (Ncan) overlapped with the α-O-Man antibody signal in granular cell layer in the cerebellum (see merge), but was absent from Purkinje cell layer (asterisk; see also single channel images). B) Cerebellar distribution of versican (Vcan) was comparable to that of the α-O-Man antibody. Vcan staining was primarily detected in the granular cell layer (bracket) and Purkinje cell layer (asterisk). C) Neurons positive for the α-O-Man antibody were often surrounded by glycans reactive to WFA, a marker for perineural nets (PNNs), in the cerebral cortex (CC). Scale bar = 50 μm and for inlay 25 μm.

Glycoproteomic analysis identified the perineural net (PNN) proteins neurocan and versican to be O-mannosylated ([28]; see also below). In the cerebellum, co-localization with neurocan overlapped perfectly in the granular cell layer, whilst the Purkinje cell layer was not marked by neurocan antibodies (Fig 5A). On sequential sections, versican distribution was highly comparable to the α-O-Man antibody signal (Fig 5B). To further address the presence of mono-O-mannosyl glycans in the PNN, we performed co-staining of the new α-O-Man antibody and *Wisteria floribunda* agglutinin (WFA), a frequently used marker of the PNN [38]. We detected a significant overlap of the staining, especially in the cerebral cortex (Fig 5C). In many cases, WFA staining enclosed mono-O-mannosyl glycan staining, suggesting that O-mannosylated proteins are more abundant close to cell bodies (Fig 5C, inlay).

### Identification of mono-O-mannosylated proteins from the murine brain by a glycoproteomics approach

**Fig 6:**
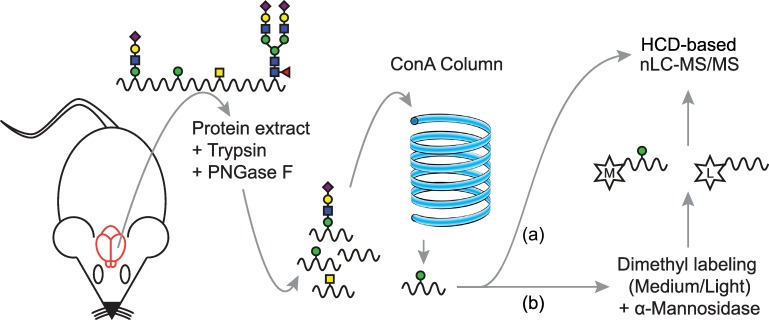
Schematic workflow for the enrichment of peptides bearing O-linked mannose. For the detailed protocol please refer to the Materials and Methods section. In brief, murine brains were extracted and pulverized before protein extraction. Protein suspensions were digested with trypsin and N-glycans removed by PNGase F treatment. Tryptic peptides were thereafter subjected to ConA LWAC to enrich for mono-O-mannosylated peptides. Elution fractions were either analyzed directly (a), or after dimethyl labeling (medium (M), light (L)) and α-mannosidase treatment (b) by LC-MS/MS and HCD fragmentation according to previously described protocols [31].

**Fig 7:**
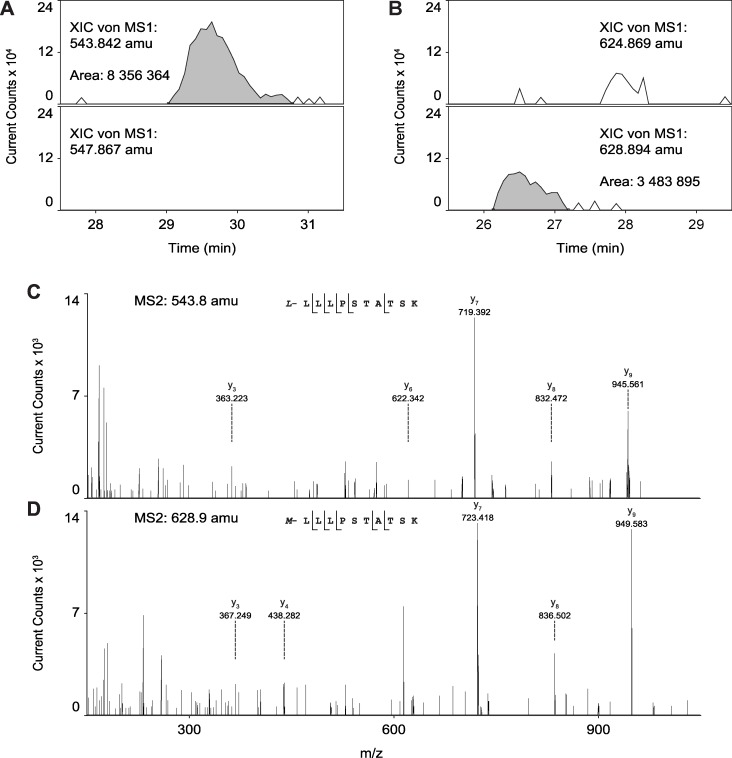
Validation of O-mannosylation on RPTPζ. Samples were dimethyl labeled in the light and medium form, respectively. After treating the light sample with α-mannosidase, both samples were mixed and analyzed by LC-MS/MS. Shown are four extracted ion chromatograms of the m/z values of light and medium labeled peptide LLLPSTATSK in (A) the deglycosylated form (543.8 amu and 547.9 amu) and in (B) the mannosylated form (624.9 amu and 628.9 amu, respectively). The glycopeptide was detected only in the untreated sample (medium labeled), whereas the deglycosylated peptide (light labeled) was only observed after mannosidase treatment. (C) HCD fragment spectrum of precursor mass 543.8 amu and (D) 628.9 amu confirmed the sequence of the deglycosylated and O-mannosylated peptide LLLPSTATSK of RPTPζ.

**Fig 8:**
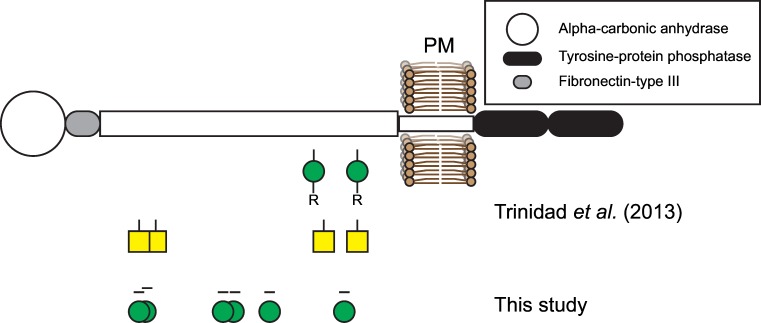
Localization of O-mannosylated peptides of human RPTPζ. Schematic model of RPTPζ, illustrating the annotated domains. Plasma membrane (PM) is indicated as a lipid bilayer. Positions of peptides containing extended O-mannosyl glycans (indicated by green circles and -R) and O-linked N-Acetylgalactosamine mucin-type glycans (yellow squares) found by Trinidad and coworkers are shown [29]. The position of O-hexosylated peptides identified in this study is indicated as a green circle with a horizontal black bar.

Although O-mannosyl glycans are abundant, so far only few of the target proteins have been identified from mammalian brain. To explore the portfolio of mono-O-mannosylated proteins, we used previously established strategies for the detection of O-mannosylated peptides [31, 32]. The herein applied workflow is summarized in Fig 6. Briefly, proteins were extracted from brains of 8–12 weeks old mice as detailed in Materials and Methods. After digestion with trypsin, peptides/glycopeptides were treated with PNGase F to remove N-linked glycans. Glycopeptides bearing O-linked mannoses were then extracted by the mannose-binding lectin ConA using LWAC following the previously described protocol of Vester-Christensen and coworkers [31]. O-mannosylated peptides were identified by higher-energy collision-induced dissociation (HCD)-based mass spectrometry leading to informative peptide fragments due to a complete loss of the glycan moieties from the precursor ion masses, either with or without the combination of α-mannosidase treatment, and liquid chromatography (LC)-MS after differential tagging using stable isotopes. For the latter approach, samples were divided into two parts and peptides labeled with either normal or deuterated formaldehyde. In one of the samples O-linked mannoses were clipped off with α-mannosidase. After inactivation of the enzyme, de-mannosylated and mock treated peptides were combined and analyzed by LC-MS. We succeeded to unambiguously identify a total of 16 glycoproteins that are summarized in Table 1 (see also S6 Fig). LC-MS analysis of α-mannosidase treatment was obtained for a fraction of these peptides (shown in Table 1 (bold letters), Fig 7 and S7 Fig). For RPTPζ, we identified a total of six new glycopeptides (Table 1, Fig 8 and S8 Fig). Further, nine of the identified glycoproteins are members of the cadherin superfamily of cell-adhesion proteins, including the type II cadherin CDH11, the glycosylphosphatidylinisotol (GPI)-anchored CDH13 and seven protocadherins (Pcdhs). Amongst those, for Fat3, Pcdhgb5 and Pcdhga12 O-mannosylation is described for the first time. We found two members of the plexin family (Plxna1 and Plxnb2), and the ER-localized protein disulfide isomerase Pdia3, which, like cadherins, have been described to be O-mannosylated previously in a “SimpleCell” system [31], and the PNN protein neurocan. In addition, mono-O-mannosyl glycans were identified for the first time on neurexin 3 (Nrxn3), a known interactor of α-DG [46], and on inter-alpha-trypsin inhibitor heavy chain family member 5 (Itih5), a modulator of the extracellular matrix [47].

**Table 1:** Mono-O-mannosylated glycoproteins from murine brain.

UniProt	Gene name	Published	Sequence	Nr. of hexoses	PEP	Score	Δ Score
P55288	Cdh11	[1]	**DMGGHMGGLSGTTK**	2	4.4E-07	64.5	54.8
Q9WTR5	Cdh13	[1]	EGPYIGHVMEGSPTGTTVMR	1	4.5E-155	156.8	138.5
Q9QXA3	Fat1	[1]	EDVPTGSSVMTVSAHDEDTGR	2	1.9E-36	100.2	87.3
Q8BNA6	Fat3		VTDQGSPPMSATAIVR	2	7.7E-04	46.4	37.8
Q8BJD1	Itih5		QPEPDLKKTYDPR	1	1.5E-03	48.6	27.0
P55066	Ncan	[2]	AQGMPTLTSTSSEGHPEPK	2	2.0E-03	41.5	36.2
Q6P9K9	Nrxn3		NVPTANPTEPGIR	1	1.2E-03	58.8	47.9
E9PXQ7	Pcdh10	[1]	TGTALLTIR	2	3.4E-19	100.0	65.1
F8VPK8	Pcdh9	[1]	**YSTVGVITVTDEDAGENK**	2	2.6E-12	76.9	68.9
Q91Y09	Pcdhac2	[1]	**LNASDPDEGSNGELR**	2	5.2E-10	87.7	76.1
Q91XY7	Pcdhga12		GASIASVTAHDPDSDK	2	9.1E-21	85.4	70.0
Q91XX5	Pcdhgb5		LPENVPPGTTVLR	1	2.4E-03	68.5	48.8
P27773	Pdia3	[1]	**YGVSGYPTLK**	1	6.1E-08	88.0	73.3
P70206	Plxna1	[1]	LTITGENLGLR	1	1.5E-03	50.9	28.3
B2RXS4	Plxnb2	[1]	QGPQAGGTTLTINGTHLDTGSK	1	2.7E-09	62.8	48.6
B9EKR1	Ptprz1	[3]	EPQVSTTTHYNHMGTK	1	3.1E-11	66.2	53.4
B9EKR1	Ptprz1	[3]	TSMVSQIESPR	3	1.2E-10	89.5	74.7
B9EKR1	Ptprz1	[3]	SDVPNTSPNSTSQHVAEFETER	1	3.0E-05	40.6	34.3
B9EKR1	Ptprz1	[3]	HLHTVSQTLPQVTSAAER	4	8.1E-05	46.9	35.2
B9EKR1	Ptprz1	[3]	MSSFSDMAYPSK	1	4.1E-04	70.2	56.6
B9EKR1	Ptprz1	[3]	**LLLPSTATSK**	1	2.0E-06	77.2	49.9
[1] see [31]							
[2] see [28]							
[3] see [29]							
In bold: Mono-O-mannosylated peptides validated by α-Mannosidase treatment [32].							
Underlined: Corresponding peptides identified in the referred publications.							

Extracted proteins were digested, O-mannosylated peptides enriched by ConA-lectin chromatography, and identified by LC-MS/MS. The quality of peptide identification is indicated by PEP, Score, and Δ Score calculated by MaxQuant [36]. The UniProt accession numbers of all database entries containing the identified peptide sequence are listed.

## Discussion

Since the discovery of O-mannosyl glycans associated to rat proteoglycans [48], considerable efforts have been made to characterize O-mannosyl glycan structures and to understand their functions. An early study of Chai and co-workers showed that 30% of the total O-linked glycans released from rabbit brain extracts belong to the O-mannosidic type [21]. Today, in the mouse brain a wide variety of O-mannosyl glycan structures is known, including linear core M1 and branched core M2 glycans, the core M3 matriglycan and also unsubstituted O-linked mannoses [5, 24]. To investigate the distribution of O-mannosyl glycans *in vivo*, we established a monoclonal antibody which, to the best of our current knowledge, specifically detects mono-O-mannosyl glycans on cryosections from the murine brain.

The monoclonal antibody α-O-Man binds to O-mannosylated proteins on Western Blots (Fig 1A). That these proteins bear not only elongated O-mannosyl residues but also unsubstituted O-linked mannoses was shown by the work of Breloy and coworkers who identified permethylated mannitol when analyzing glycan structures present on α-DG variants [34]. On glycopeptide arrays α-O-Man binds highly specifically to an O-mannose peptide derived from the POMT substrate protein KIAA1549 [31] and two peptides from α-DG [49], although to a lower extent when compared with the original antigen (S1 Fig, #17–19, 26). Two of the tested O-mannose peptides (S1 Fig, #20 and 21) as well as peptides with two adjacent threonine-linked O-mannose residues were not recognized (S1 Fig, #22–24). The new antibody showed high selectivity towards mono-O-mannosylated peptides when compared to the same peptides bearing linear Galß1,4GlcNAcß1,2Man and GlcNAcß1,2Man O-mannosyl glycans coupled to threonine (S1 Fig). Furthermore, when presented as neoglycolipid, serine- as well as threonine-linked α1-mannose was detected by α-O-Man (S2 Fig). In combination, these data suggest that the new monoclonal antibody α-O-Man prefers a specific glycopeptide conformation for recognition. In addition, underlying hydrophobic effects from the peptide backbone might further support the antibody glycan recognition.

Among the almost 500 different glycan structures tested on the NGL-based oligosaccharide microarrays serine- and threonine-linked α1-O-mannose probes were recognized by α-O-Man. The cross-reactivities of other mannose-related probes observed in the screening analysis are unlikely to be physiologically significant, as the probes that showed binding intensities similar to those for the serine- and threonine-linked α1-O-mannose probes, e.g. short α-linked mannose sequences and β1-4-linked oligomannose glycans, are not represented on murine brain glycoproteins to our knowledge. Moreover, the monoclonal α-O-Man did not target Man_3_GlcNAc_2_, when presented in a peptide surrounding (S4 Fig). Hybrid or complex N-glycans were not recognized at all (S2 Fig). Thus, it is highly unlikely, that minor interactions with N-glycans impact on the immunofluorescence analyses presented. This is further supported by the fact that PNGase F treatment of cryosections revealed unaltered distribution and intensity of α-O-Man staining, whereas the primarily N-glycan dependent WFA-staining [39, 40] was almost completely lost after N-glycan removal (Fig 1C). Moreover, the α-O-Man immunostaining of the murine brain is highly dependent on functional protein O-mannosylation, as demonstrated by the absence of cell surface staining upon O-mannosylation inhibition in Madin-Darby canine kidney cells (S5B Fig) and the strong reduction in signal intensity in POMT2^f/f;Emx1-Cre+^ mouse cerebral cortex (Fig 2B). The weak residual α-O-Man signal obtained in the latter mice is most likely due to the conditional knockout strategy not targeting all of the neuronal cells. Importantly, the pre-absorption of the α-O-Man antibody with the O-mannosylated peptide obliterated the neural tissue immunostaining (S5A Fig). In addition, expression of the POMT2 mannosyltransferase (revealed by POMT2-directed polyclonal antibody staining) is high in cells with abundant α-O-Man immunostaining, such as the Purkinje cell layer in the cerebellum, and the *cornu ammonis* in the hippocampus (S9 Fig) further supporting the significance of our findings.

We found that mono-O-mannosyl glycans are prevalent in inhibitory GABAergic neurons, such as the Purkinje cells in the cerebellum, as demonstrated by gephyrin co-staining and co-localization with the cre protein expressed under the control of a GABAergic neuron-specific promoter (Figs 2 and 3). Various known O-mannosylated proteins localize to the GABAergic Purkinje cell layer (Fig 4), such as α-DG and RPTPζ [50], or are involved in Purkinje cell placement like Plxnb2 [51]. The dystrophin-associated glycoprotein complex component α-DG and α-DG O-mannosylation have been demonstrated to be not only important for neuroblast formation and axon guidance during development of the nervous system, but also affect synaptic plasticity at GABAergic synapses in the adult brain [52–54]. RPTPζ was identified in this study to be subject to substantial modification by mono-O-mannoses (Table 1, Figs 7 and 8 and S8 Fig). RPTPζ contributes to a diverse array of biological processes required for normal development and brain function, including regulation of the formation of Purkinje cell dendrites [50] and aberrant glycosylation of RPTPζ has been observed in a MEB mouse model lacking functional POMGnT1 [30]. So far, only branching of O-mannosyl glycans by GnT-Vb was investigated in respect to the function of O-mannosyl glycans on RPTPζ [25]. Therein, the authors showed that GnT-Vb promoted RPTPζ dimer formation, leading to an inhibition of intrinsic phosphatase activity. It will be a future challenge to investigate the functional role of unsubstituted O-mannosyl glycans on RPTPζ.

Mono-O-mannosyl glycans were further localized in the PNN which provides structural stability of the brain tissue (Fig 5). Mono-O-mannosyl glycan staining significantly overlaps with that of the lecticans (e.g., versican and neurocan, Fig 5A and 5B) which interact with hyaluronan and other extracellular matrix proteins to form the PNN [55]. Our glycoproteomics analysis further demonstrated mono-O-mannosylation of neurocan (Table 1, S6 Fig). These findings are in good agreement with recent biochemical data that showed O-mannosylation of neurocan and versican in high molecular weight protein extracts from bovine and mouse brain [27, 28]. In these studies, O-glycans released from lectican-positive fractions, were analyzed by MALDI-MS/MS and proteins were identified by LC-ESI-MS/MS.

Glycoproteomics revealed mono-O-mannosyl glycans on neurexin-3, marking the first interactor of α-DG to carry O-mannosylation, and inter-alpha-trypsin inhibitor heavy chain family member 5 (Itih5), being yet another protein involved in the upkeep and remodeling of the extracellular matrix. The main carriers of mono-O-mannosyl glycans identified herein belong to the cadherin and plexin family, yielding 7 and 2 members, respectively (Table 1, S6 and S7 Figs). O-mannosylation of numerous members of these protein families has been recently demonstrated in simplified human breast cancer cells [31], but, with the exception of CDH13, not yet from native tissues. Further, for Fat3, Pcdhgb5 and Pcdhga12 O-mannosylation is described for the first time. The fact that the largest portion of proteins identified are cadherin and plexin family members, demonstrates mono-O-mannosylation of these families *in vivo*.

Moreover, we identified mono-O-mannosyl glycans on the ER-localized protein disulfide isomerase Pdia3 (Table 1, S6 and S7 Figs). PDIs transfer oxidizing equivalents to newly synthesized polypeptides, leading to the formation or rearrangement of protein disulfide bonds. Thereby they fulfill a crucial role for oxidative protein folding in the ER which is an essential function of eukaryotic cells (reviewed in [56]). The mannosylated peptide identified in mouse Pdia3, includes a conserved ProThr-motif that has been demonstrated to carry an O-linked mannose in PDIA3 from human breast cancer cells [31] (S10 Fig). Most interestingly, we very recently found O-mannosyl glycans also in the ER-luminal PDIs and PDI-like proteins from baker´s yeast and found that modification by O-mannosylation occurred in a conserved residue in the proximity of the catalytic CysXXCys motifs [57].

The combination of *in situ* staining and glycoproteomics illustrates that mono-O-mannosylation is abundant in the mammalian brain and targets many glycoproteins. Numerous of these proteins are possible contributors to the neurological abnormalities observed in WWS and other α-dystroglycanopathy patients. However, it will be a challenge to address this issue since in animal models that lack O-mannosylation the concomitant aberrant glycosylation of α-DG drives highly dominant neurological phenotypes.

## Supporting Information

S1 Fig The monoclonal α-O-Man antibody specifically recognizes unsubstituted O-mannose linked to threonine in a diverse peptide context. Microarray analysis elucidating the monoclonal antibody RKU-1-3-5 antibody recognition of *O*-mannosyl peptides **7–31** at different antibody concentrations. Strong recognition of antigen peptide **26** and weak recognition of *O*-mannosyl peptides **17–19** were observed. Fluorescence read-out after incubation with a biotin labeled secondary anti-rabbit IgG antibody and streptavidin Cy5. The diagram shows mean values and standard deviations of 5 spot replicates per peptide. Peptide backbones were the same for peptides no. **8**, **17**, **28** (PVPGKPTVTIR), no. **9**, **18**, **29** (RGAIIQTPTLG), no. **30**, **31** (GTG), no. **7**, **10**, **25** (YATAVA), no. **11**, **19** (SQSLEETISPR), no. **12**, **20** (SGPLDGGTLLTIR), no. **13**, **14**, **21**, **22** (NAPSGTTVIHLNA), no. **15**, **23** (QGPQAGGTTLTIHG), no. **16**, **24** (EPGGSYITTVSATD) and no. **26**, **27** (YATAV). Green circles, blue squares and yellow circle represent mannose, N-Acetylglucosamine and galactose, respectively. Linkage conformations are indicated. For a comprehensive summary of the peptides used in this array and their protein origin please refer to S3 Table.
(TIF)

S2 Fig The α-O-Man antibody recognizes serine- and threonine-linked mannoses among the mammalian type glycan sequences. A) The 492 lipid-linked probes are arranged according to their backbone sequences as annotated in the coloured panels below the Fig Lac, lactose; LacNAc, N-acetyllactosamine; LNnT, lacto-N-neotetraose; LNT, lacto-N-tetraose; PolyLac, polylactosamine; GAGs, glycosaminoglycans; Misc., miscellaneous. The signals are means of fluorescence intensities of duplicate spots, printed at 5 fmol with error bars representing half of the difference between the two values. X denotes signal with large error bar (no significant binding). The signals shown together with the probe sequences are in S3 File. In separate experiments (not shown) binding signals were not detected when using the detection antibody, biotinylated anti-rabbit IgG. B) Structures of O-mannosyl serine or threonine related probes investigated in the microarray. DHPE, 1,2-dihexadecyl-sn-glycero-3-phosphoethanolamine; Succ, succinic anhydride linker.
(TIF)

S3 Fig Results for 15 probes selected from ‘N-glycan related Array Set 1’ at different dilutions of RKU-1-3-5 antibody. Probes of oligo- and high-mannose type N-glycans and O-mannosyl serine and threonine probes are shown here. The complete list of probes in the array, their sequences and binding scores are provided in supplemental S2 Table.
(TIF)

S4 Fig The N-glycan structure Man_3_GlcNAc_2_ is not identified by α-O-Man in a varying peptide context. Microarray analysis elucidating a) the mAb RKU1-3-5 antibody recognition of the pentasaccharide core Man_3_GlcNAc_2_ *N*-glycopeptides **38–41**, no recognition; b) positive control, Biotin-ConA, recognition of all pentasaccharide core glycopeptides. Fluorescence read-out: a) incubation with a biotin labeled secondary anti-rabbit IgG antibody and then streptavidin Cy5; b) incubation with streptavidin Cy5. The diagrams shows mean values and standard deviations of 5 spot replicates per peptide. Peptide sequences are: no. **38** VVN*STTGPGEHLR, no. **39** WVSN*KTEGR, no. **40** N*LTALPPDLPK and no. **41** LQNLTLPTN*ASIK (an asterisk marks the site of the pentasaccharide). Green circles and blue squares represent mannose and N-Acetylglucosamine, respectively. Linkage conformations are indicated. For further information on the used peptides see S4 Table.
(TIF)

S5 Fig Preadsorption studies of α-O-Man antibody and comparison of α-O-Man antibody reactivity on Madin-Darby canine kidney cells. A) Pre-adsorption with an O-mannosylated peptide completely abrogated signal pattern obtained by the α-O-Man antibody on wild-type (WT) murine cerebellar cryosections (right panel), whereas pre-adsorption with the corresponding peptide lacking the O-linked mannose did not influence the staining. Nuclei were counterstained with DAPI, sections were cut sagittally. B) Madin-Darby canine kidney cells were grown in the presence or absence of O-mannosylation inhibitor R3A-5a [8] for three days. Fixed cells were stained with a monoclonal antibody (this study; α-O-Man) or a previously described polyclonal antibody (T[α-1Man]; [8]), both of which were raised against a peptide harboring an α-1-mannosylated threonine residue. Inhibitor-treatment completely abolished staining of cell-contact sites. Cellular nuclei are counterstained with DAPI. C) Co-localization of α-DG using α-IIH6 and α-O-Man on sequential sections showed substantial overlap, for example in the Purkinje cell layer (asterisk), whereas vasculature (arrowhead) was not labeled by α-O-Man antibody. Scale bar = 50 μm (A/C),10 μm (B).
(TIF)

S6 Fig Peptide spectra from HCD analysis. Glycopeptides identified in HCD-MS/MS analysis. Individual HCD fragment spectra/patterns of all identified glycopeptides are given. y-fragments are colored red and b-fragments are colored blue. Peptide sequence and the corresponding fragment patterns are depicted below each spectrum. Deamidation at asparagine is indicated by “de”. Presence of glycosylation was determined by neutral loss of hexoses from the precursor ion mass. Localization of glycosylation can be ambiguously mapped to serine and threonine residues. Position of hexose indicator “he” is based on the suggestion by MaxQuant.
(PDF)

S7 Fig Validation of O-mannosylation of selected peptides. Samples were dimethyl labeled in the light and medium form, respectively. After treating the light sample with α-mannosidase both samples were mixed and analyzed by LC-MS/MS. Shown are four extracted ion chromatograms of the m/z values of light and medium labeled peptides for CDH11 (A), deaminated PCDHAC2 (B), PDIA3 (C) and PCDH9 (D). Inlay picture 1 always shows the respective deglycosylated, whereas inlay picture 2 refers to the mannosylated form. The glycopeptide was detected only in the untreated sample (medium labeled), whereas the deglycosylated peptide (light labeled) was only observed after mannosidase treatment. Inlay picture 3 shows HCD fragment spectrum of the respective precursor mass and inlay picture 4 confirms the sequence of the deglycosylated and O-mannosylated peptide of the respective protein.
(PDF)

S8 Fig Localization of O-mannosylated peptides in human RPTPζ. Amino acid sequence of the extracellular domain of human RPTPζ. O-hexosylated peptides identified in this study are given in bold red, whereas peptides previously identified by Trinidad and coworkers are highlighted in bold green [29].
(TIF)

S9 Fig Distribution of POMT2 in WT murine brain cryosections. Sagittal cryosections of WT murine brain were stained with the previously described α-POMT2 antibody (see S1 File). As a control, POMT2 antibodies were preadsorbed to nitrocellulose-bound recombinant epitope and the resulting supernatant was used for immunodetection (pread α-POMT2; for details see S1 File section). POMT2 signal distribution was comparable to α-O-Man immunostaining (compare to Fig 2) and completely absent after preadsorption. Nuclei were counterstained with DAPI. Scale bar = 50 μm.
(TIF)

S10 Fig Conservation of amino acids in protein disulfide-isomerase A3 (PDIA3). Alignment of sequences for PDIA3 protein or respective homologues from different species including, from top to bottom, *Saccharomyces cerevisiae*, *Caenorhabditis elegans*, *Xenopus laevis*, *Danio rerio*, *Gallus gallus*, *Rattus norvegicus*, *Bos taurus*, *Mus musculus* and *Homo sapiens*. The sequence is an extract from both Thioredoxin domains. Similarity is indicated by “.”, high similarity by “:” and identical amino acids by” *”. Identified glycopeptides from mouse brain (this study) and human breast cancer cells [31] are depicted in bold green letters. Bold red letters indicate O-mannosylated serine or threonine residues.
(TIF)

S11 Fig Synthesis outline for the glycopeptides used in this study. A) An overview of the Fmoc-Solid-phase peptide synthesis of the O-mannosyl glycopeptides. B) An overview of the Fmoc-Solid-phase peptide synthesis of the N-glycopeptides. Synthesis yields are given in parenthesis.
(TIF)

S1 File Supplementary methods. (DOCX)

S2 File HPLC chromatograms of synthesized glycopeptides used in the peptide glycoarrays. (DOCX)

S3 File Data file of NGL-based microarray screening analysis: List of 492 probes included in the NGL-based microarray screening analysis, their sequences and the fluorescence intensities at 5 fmol per probe spot of binding with monoclonal antibody RKU-1-3-5. (XLSX)

S1 Table Antibody and lectins used in this study. Monoclonal or polyclonal antibodies are indicated with m or p, respectively. Vendors and order numbers are provided, Antibody IDs from the Antibody Registry are indicated when available and used dilutions are given.
(DOCX)

S2 Table Tabulation of 52 probes included in N-glycan related Array Set 1 and their binding scores (fluorescence intensities at 5 fmol per probe spot) at different RKU-1-3-5 antibody dilutions. (DOCX)

S3 Table Synthetic O-mannosyl peptides used in the antibody microarray analysis. Glycan positions are indicated in the peptide sequence by an asterisk (*). Spacers are indicated in the peptide sequence (*Sp*).
(DOCX)

S4 Table Synthetic N-glycopeptides used in the mAb RKU-1-3-5 antibody microarray analysis. Glycan positions are indicated in the peptide sequence by an asterisk (*). Spacers are indicated in the peptide sequence (*Sp*).
(DOCX)
