# Supplementary figures and images for: Protein O-Mannosylation in the Murine Brain: Occurrence of Mono-O-Mannosyl Glycans and Identification of New Substrates
Source: PLoS One. 2016 Nov 3;11(11):e0166119. doi: 10.1371/journal.pone.0166119 (PMC5094735; doi:10.1371/journal.pone.0166119)

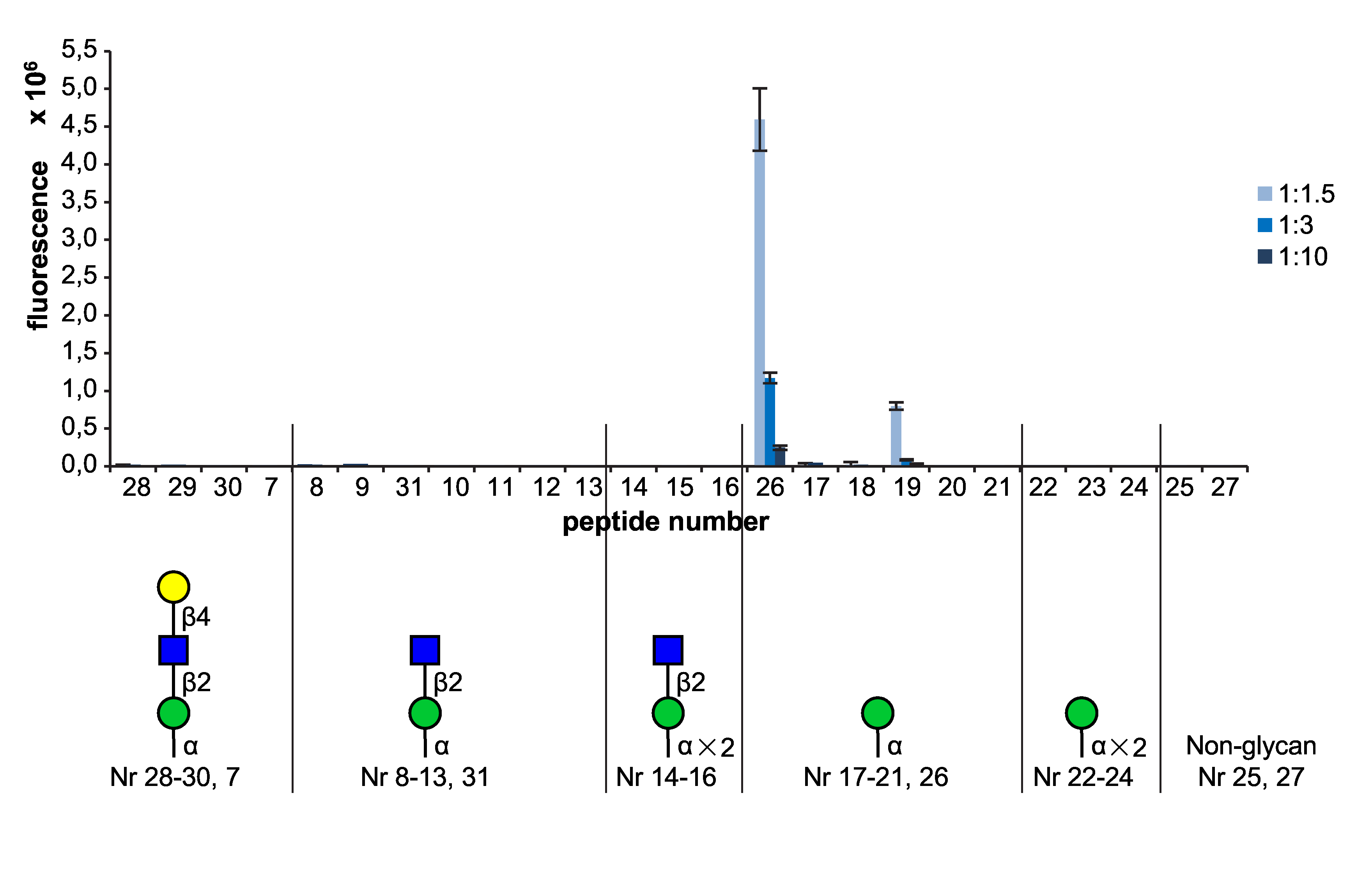

Supplement: S1 Fig — Microarray analysis elucidating the monoclonal antibody RKU-1-3-5 antibody recognition of O-mannosyl peptides 7–31 at different antibody concentrations. Strong recognition of antigen peptide 26 and weak recognition of O-mannosyl peptides 17–19 were observed. Fluorescence read-out after incubation with a biotin labeled secondary anti-rabbit IgG antibody and streptavidin Cy5. The diagram shows mean values and standard deviations of 5 spot replicates per peptide. Peptide backbones were the same for peptides no. 8, 17, 28 (PVPGKPTVTIR), no. 9, 18, 29 (RGAIIQTPTLG), no. 30, 31 (GTG), no. 7, 10, 25 (YATAVA), no. 11, 19 (SQSLEETISPR), no. 12, 20 (SGPLDGGTLLTIR), no. 13, 14, 21, 22 (NAPSGTTVIHLNA), no. 15, 23 (QGPQAGGTTLTIHG), no. 16, 24 (EPGGSYITTVSATD) and no. 26, 27 (YATAV). Green circles, blue squares and yellow circle represent mannose, N-Acetylglucosamine and galactose, respectively. Linkage conformations are indicated. For a comprehensive summary of the peptides used in this array and their protein origin please refer to S3 Table. (TIF) [file pone.0166119.s001.tif]

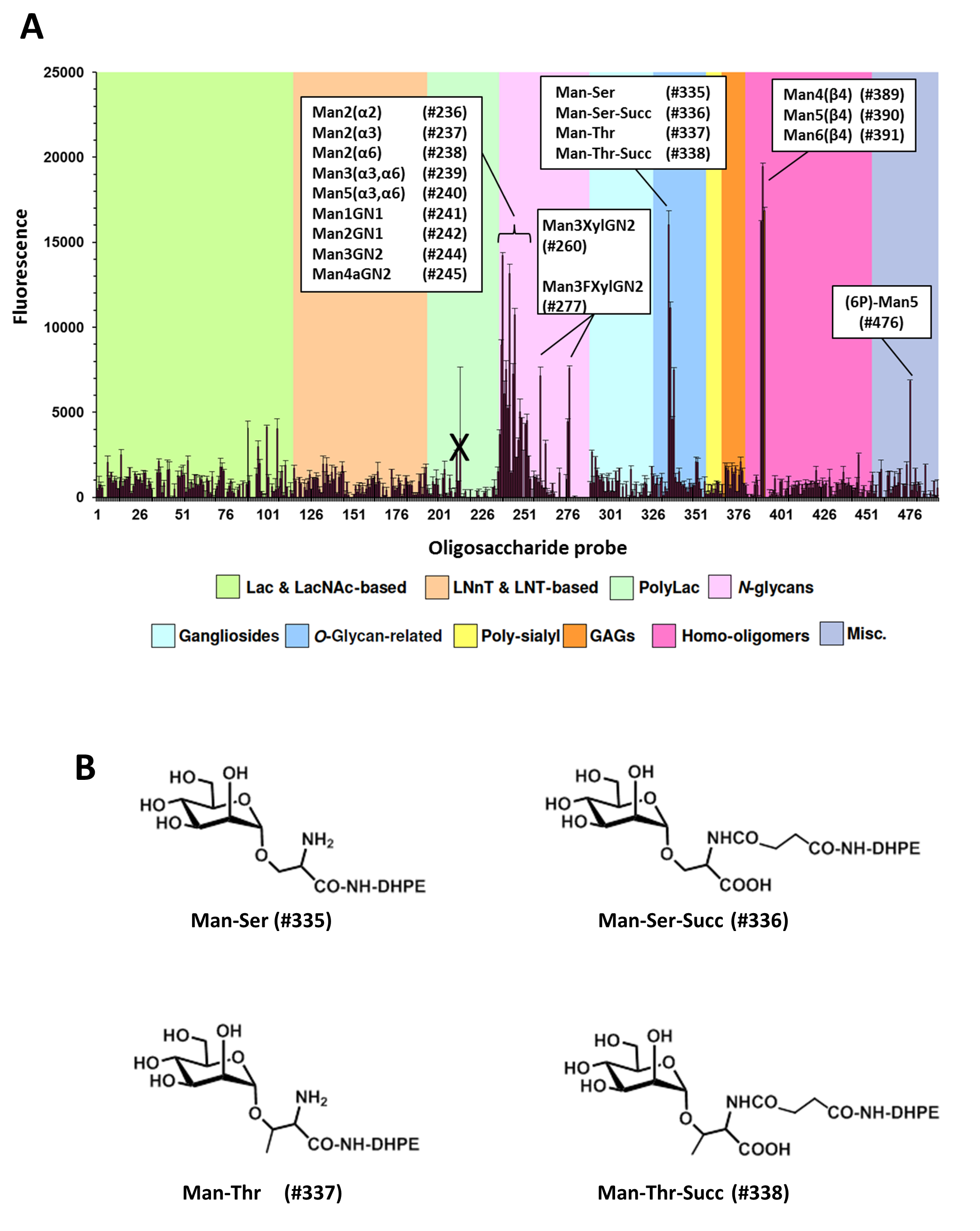

Supplement: S2 Fig — A) The 492 lipid-linked probes are arranged according to their backbone sequences as annotated in the coloured panels below the Fig Lac, lactose; LacNAc, N-acetyllactosamine; LNnT, lacto-N-neotetraose; LNT, lacto-N-tetraose; PolyLac, polylactosamine; GAGs, glycosaminoglycans; Misc., miscellaneous. The signals are means of fluorescence intensities of duplicate spots, printed at 5 fmol with error bars representing half of the difference between the two values. X denotes signal with large error bar (no significant binding). The signals shown together with the probe sequences are in S3 File. In separate experiments (not shown) binding signals were not detected when using the detection antibody, biotinylated anti-rabbit IgG. B) Structures of O-mannosyl serine or threonine related probes investigated in the microarray. DHPE, 1,2-dihexadecyl-sn-glycero-3-phosphoethanolamine; Succ, succinic anhydride linker. (TIF) [file pone.0166119.s002.tif]

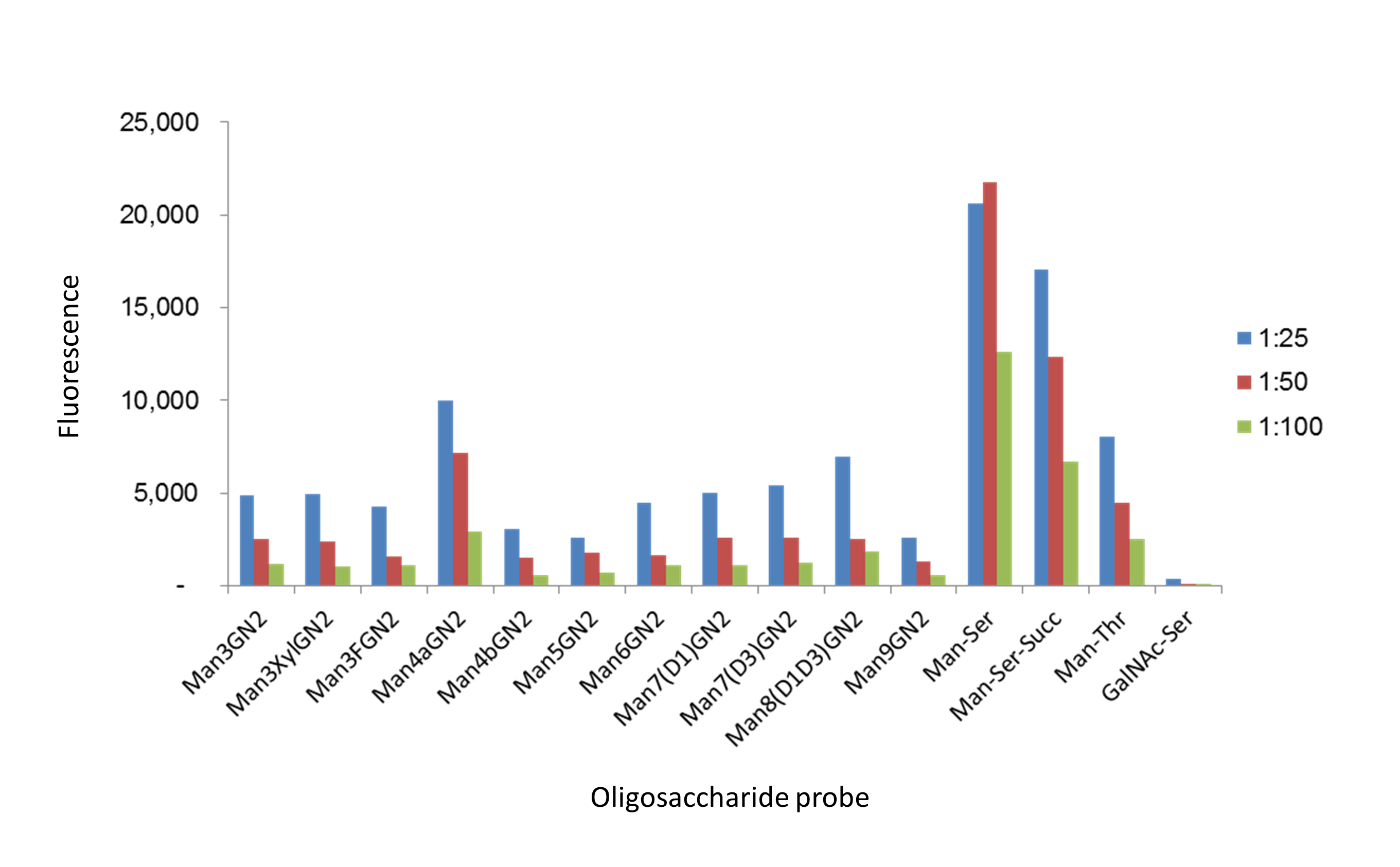

Supplement: S3 Fig — Probes of oligo- and high-mannose type N-glycans and O-mannosyl serine and threonine probes are shown here. The complete list of probes in the array, their sequences and binding scores are provided in supplemental S2 Table. (TIF) [file pone.0166119.s003.tif]

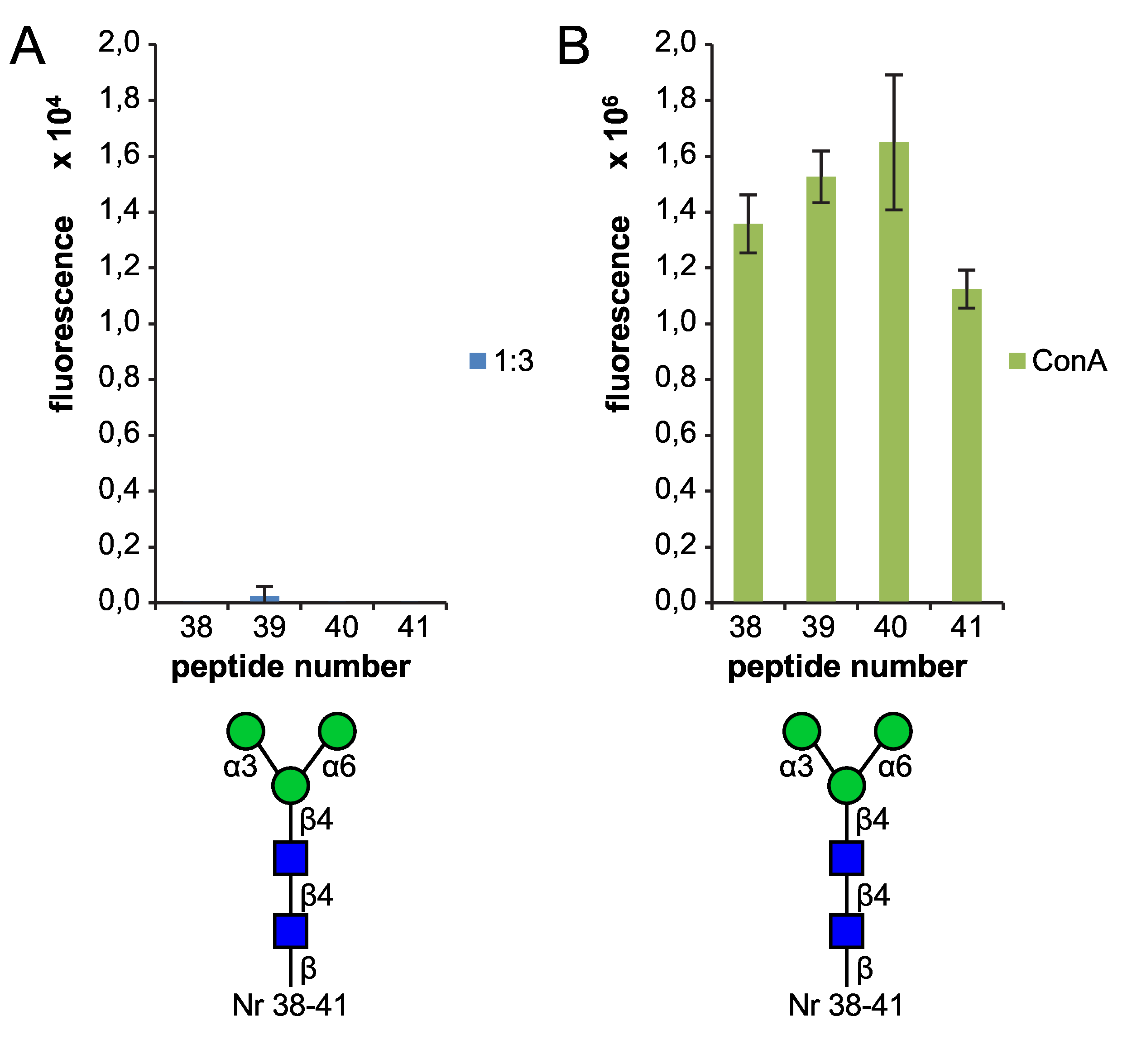

Supplement: S4 Fig — Microarray analysis elucidating a) the mAb RKU1-3-5 antibody recognition of the pentasaccharide core Man3GlcNAc2 N-glycopeptides 38–41, no recognition; b) positive control, Biotin-ConA, recognition of all pentasaccharide core glycopeptides. Fluorescence read-out: a) incubation with a biotin labeled secondary anti-rabbit IgG antibody and then streptavidin Cy5; b) incubation with streptavidin Cy5. The diagrams shows mean values and standard deviations of 5 spot replicates per peptide. Peptide sequences are: no. 38 VVN*STTGPGEHLR, no. 39 WVSN*KTEGR, no. 40 N*LTALPPDLPK and no. 41 LQNLTLPTN*ASIK (an asterisk marks the site of the pentasaccharide). Green circles and blue squares represent mannose and N-Acetylglucosamine, respectively. Linkage conformations are indicated. For further information on the used peptides see S4 Table. (TIF) [file pone.0166119.s004.tif]

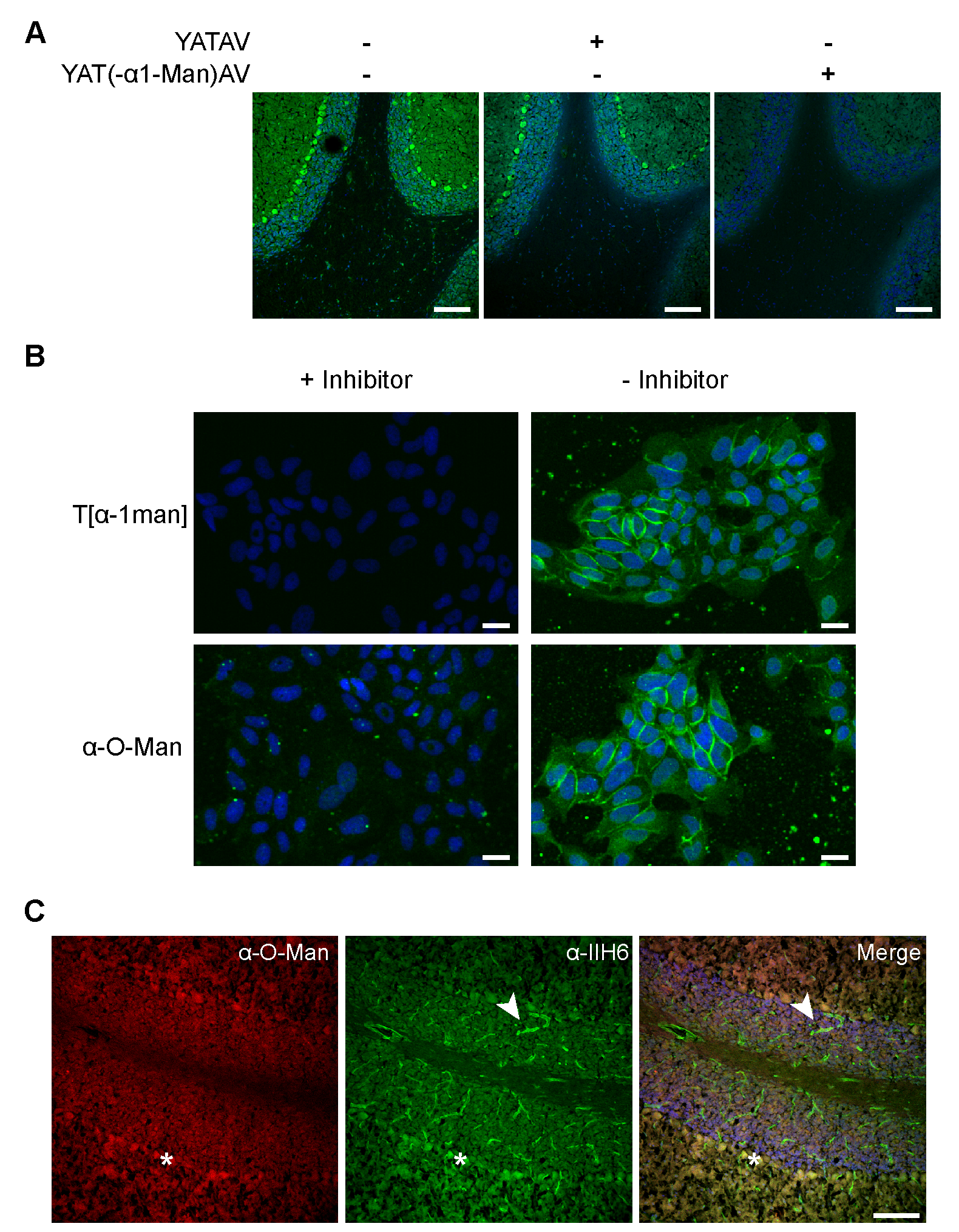

Supplement: S5 Fig — A) Pre-adsorption with an O-mannosylated peptide completely abrogated signal pattern obtained by the α-O-Man antibody on wild-type (WT) murine cerebellar cryosections (right panel), whereas pre-adsorption with the corresponding peptide lacking the O-linked mannose did not influence the staining. Nuclei were counterstained with DAPI, sections were cut sagittally. B) Madin-Darby canine kidney cells were grown in the presence or absence of O-mannosylation inhibitor R3A-5a [8] for three days. Fixed cells were stained with a monoclonal antibody (this study; α-O-Man) or a previously described polyclonal antibody (T[α-1Man]; [8]), both of which were raised against a peptide harboring an α-1-mannosylated threonine residue. Inhibitor-treatment completely abolished staining of cell-contact sites. Cellular nuclei are counterstained with DAPI. C) Co-localization of α-DG using α-IIH6 and α-O-Man on sequential sections showed substantial overlap, for example in the Purkinje cell layer (asterisk), whereas vasculature (arrowhead) was not labeled by α-O-Man antibody. Scale bar = 50 μm (A/C),10 μm (B). (TIF) [file pone.0166119.s005.tif]

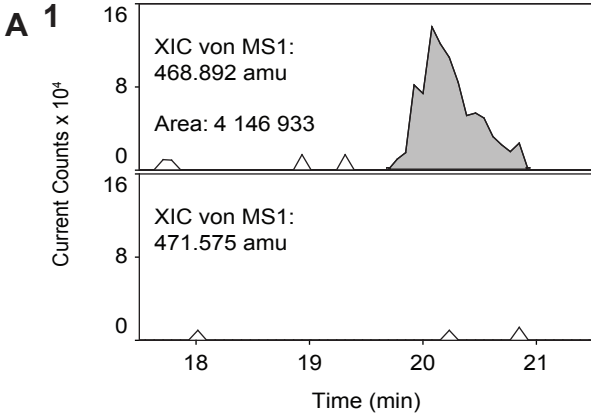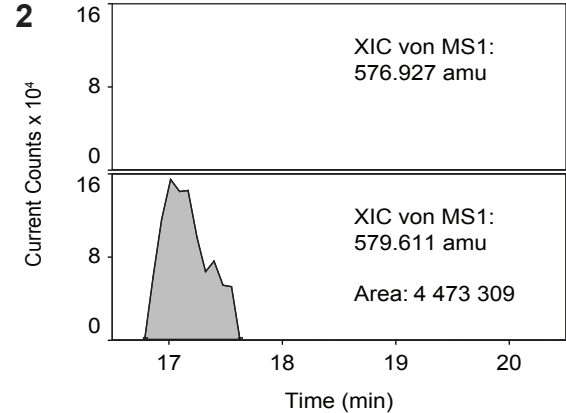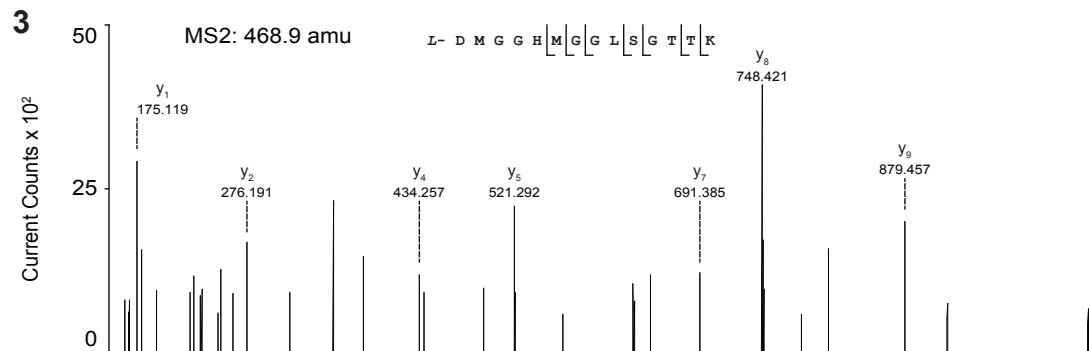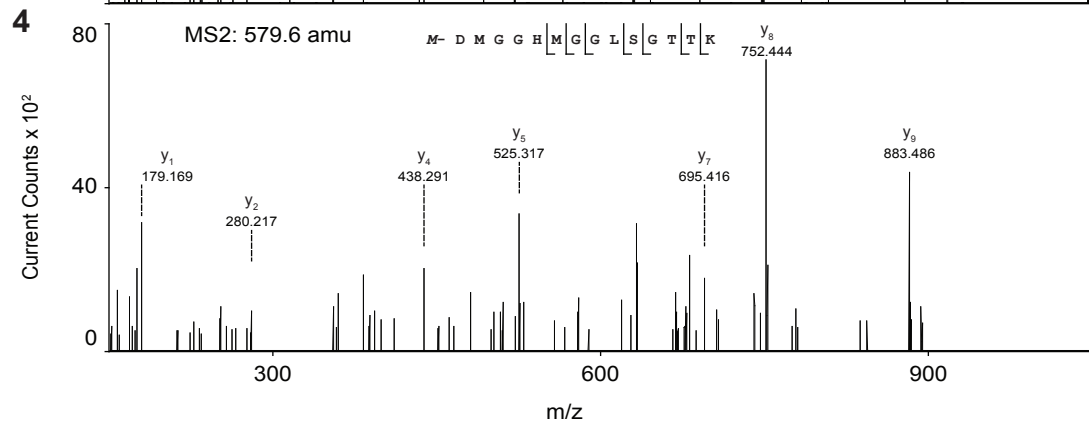

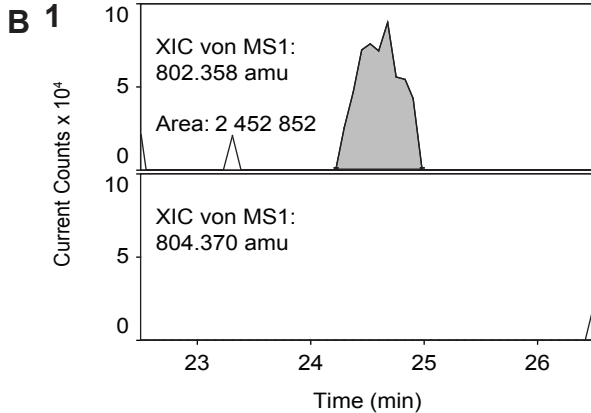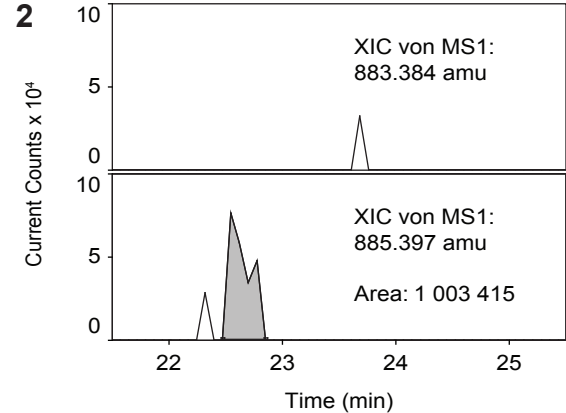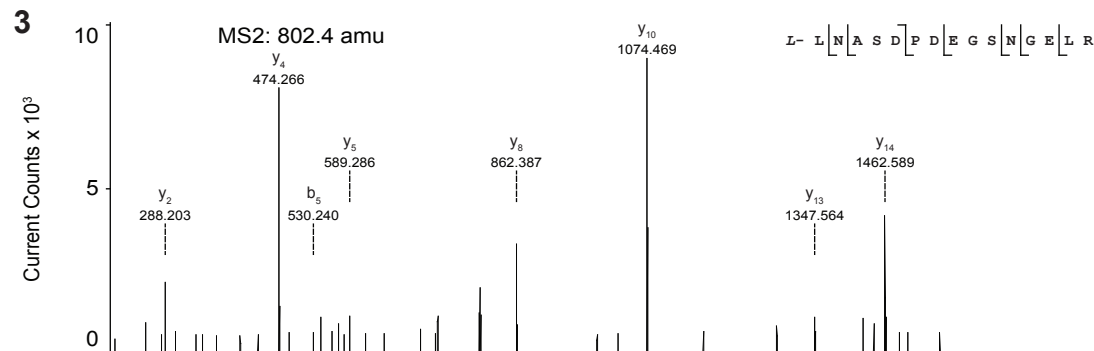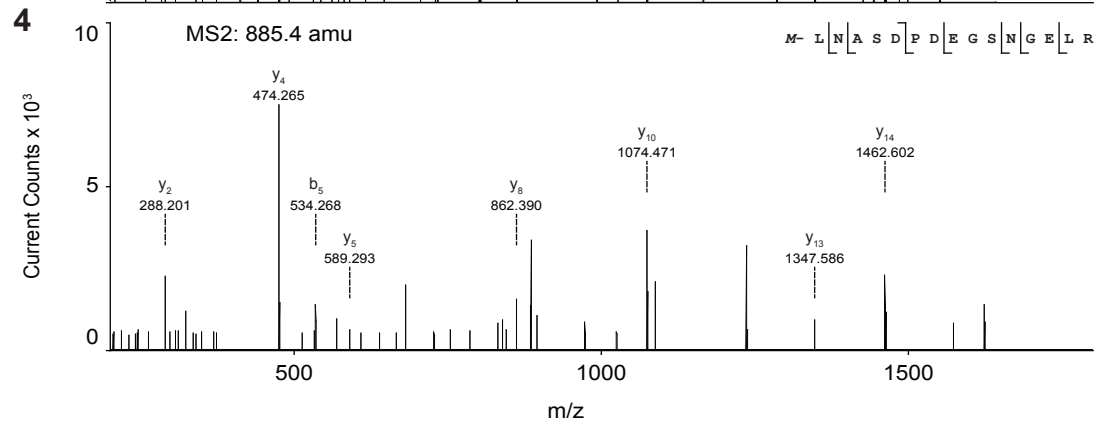

**c**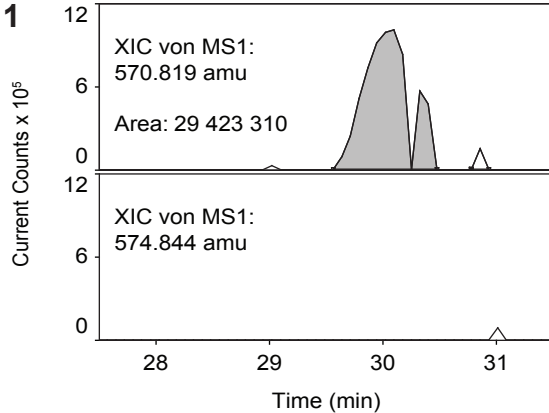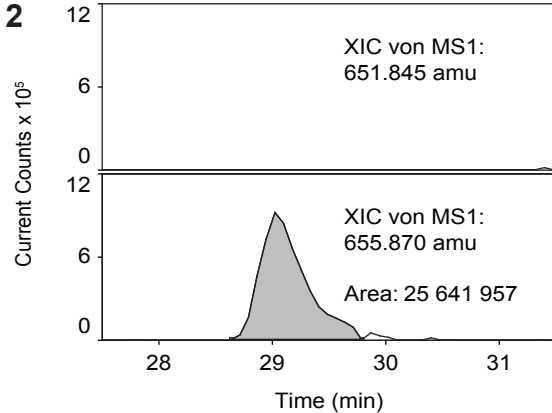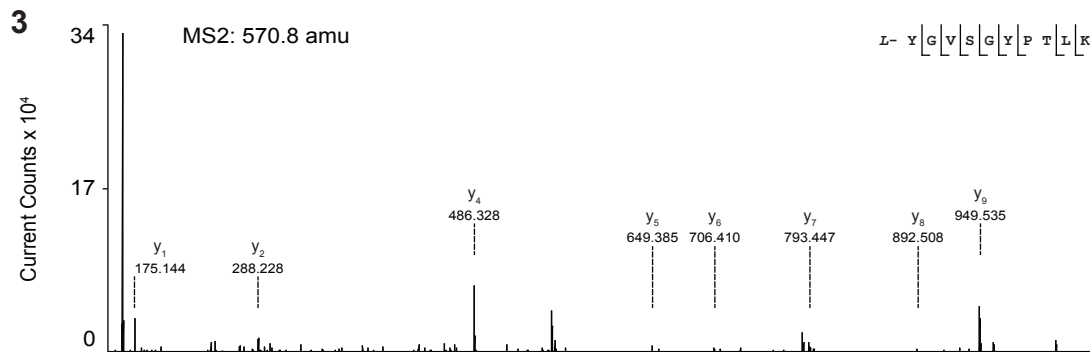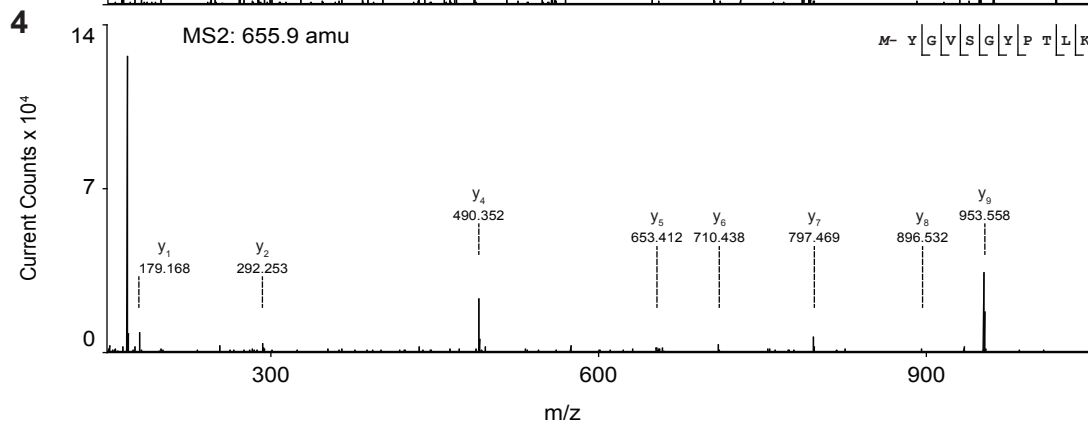

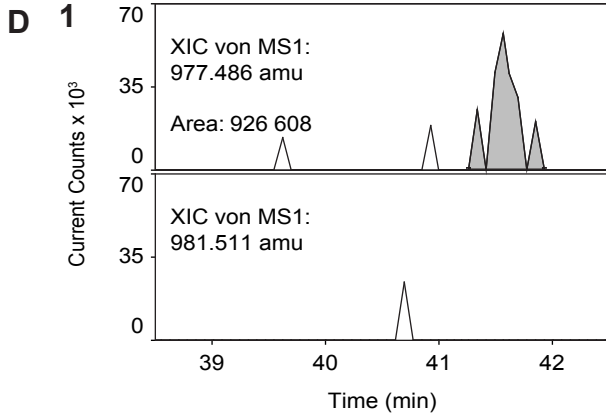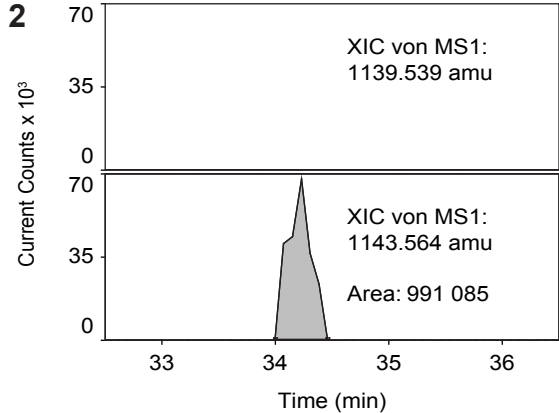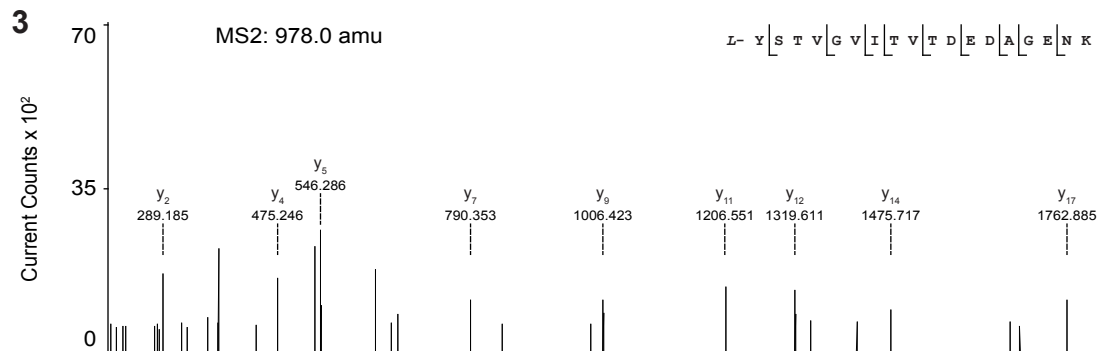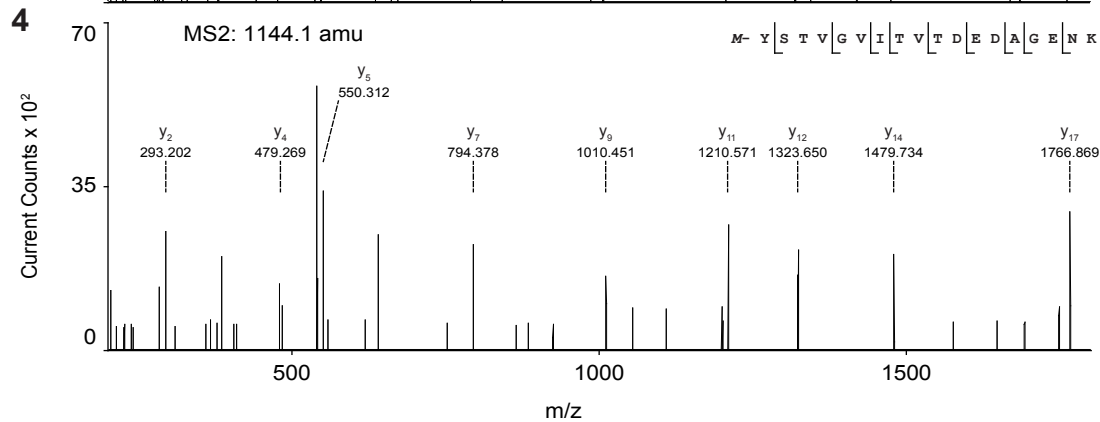

Supplement: S7 Fig — Samples were dimethyl labeled in the light and medium form, respectively. After treating the light sample with α-mannosidase both samples were mixed and analyzed by LC-MS/MS. Shown are four extracted ion chromatograms of the m/z values of light and medium labeled peptides for CDH11 (A), deaminated PCDHAC2 (B), PDIA3 (C) and PCDH9 (D). Inlay picture 1 always shows the respective deglycosylated, whereas inlay picture 2 refers to the mannosylated form. The glycopeptide was detected only in the untreated sample (medium labeled), whereas the deglycosylated peptide (light labeled) was only observed after mannosidase treatment. Inlay picture 3 shows HCD fragment spectrum of the respective precursor mass and inlay picture 4 confirms the sequence of the deglycosylated and O-mannosylated peptide of the respective protein. (PDF) [file pone.0166119.s007.pdf]

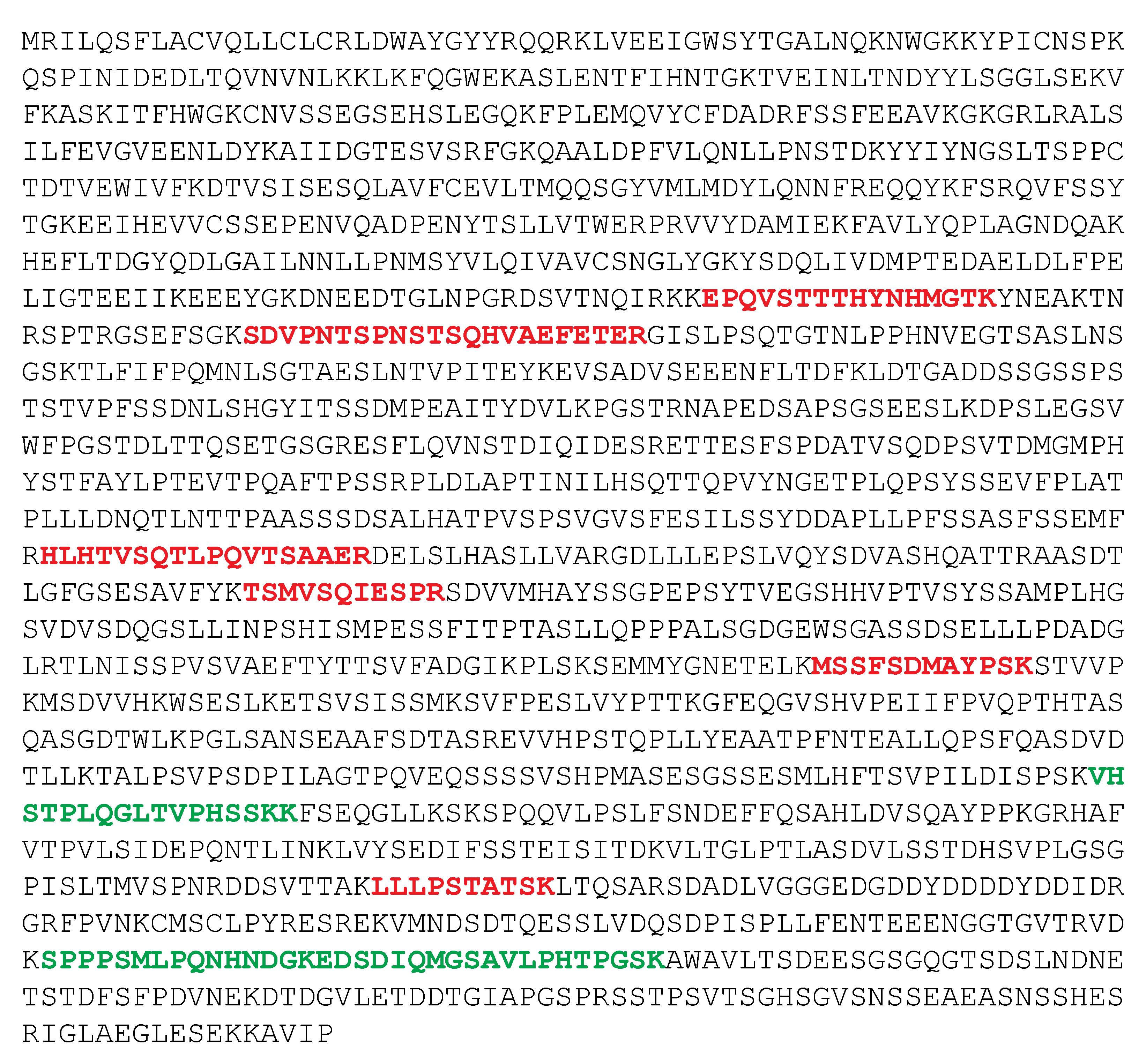

Supplement: S8 Fig — Amino acid sequence of the extracellular domain of human RPTPζ. O-hexosylated peptides identified in this study are given in bold red, whereas peptides previously identified by Trinidad and coworkers are highlighted in bold green [29]. (TIF) [file pone.0166119.s008.tif]

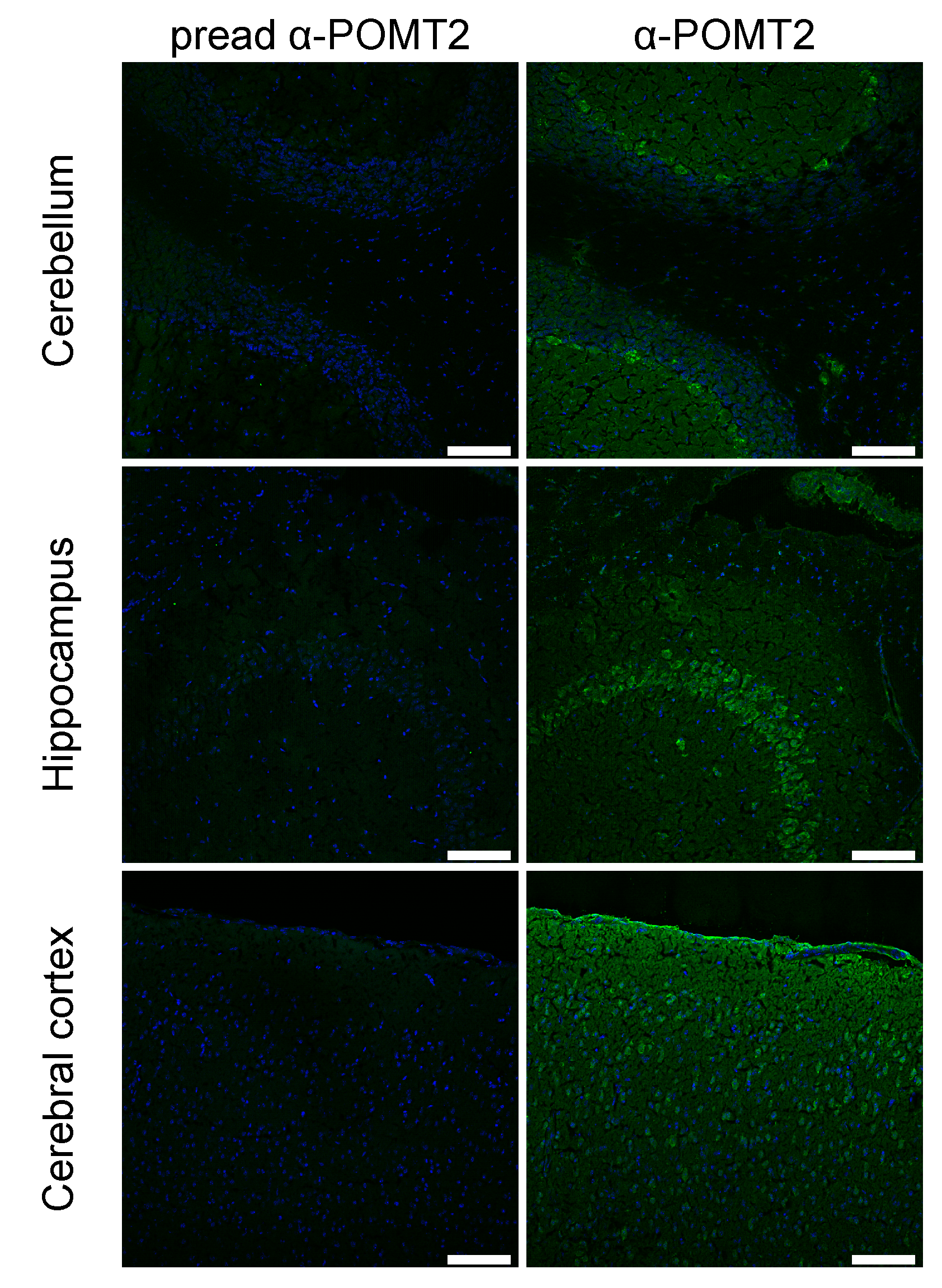

Supplement: S9 Fig — Sagittal cryosections of WT murine brain were stained with the previously described α-POMT2 antibody (see S1 File). As a control, POMT2 antibodies were preadsorbed to nitrocellulose-bound recombinant epitope and the resulting supernatant was used for immunodetection (pread α-POMT2; for details see S1 File section). POMT2 signal distribution was comparable to α-O-Man immunostaining (compare to Fig 2) and completely absent after preadsorption. Nuclei were counterstained with DAPI. Scale bar = 50 μm. (TIF) [file pone.0166119.s009.tif]

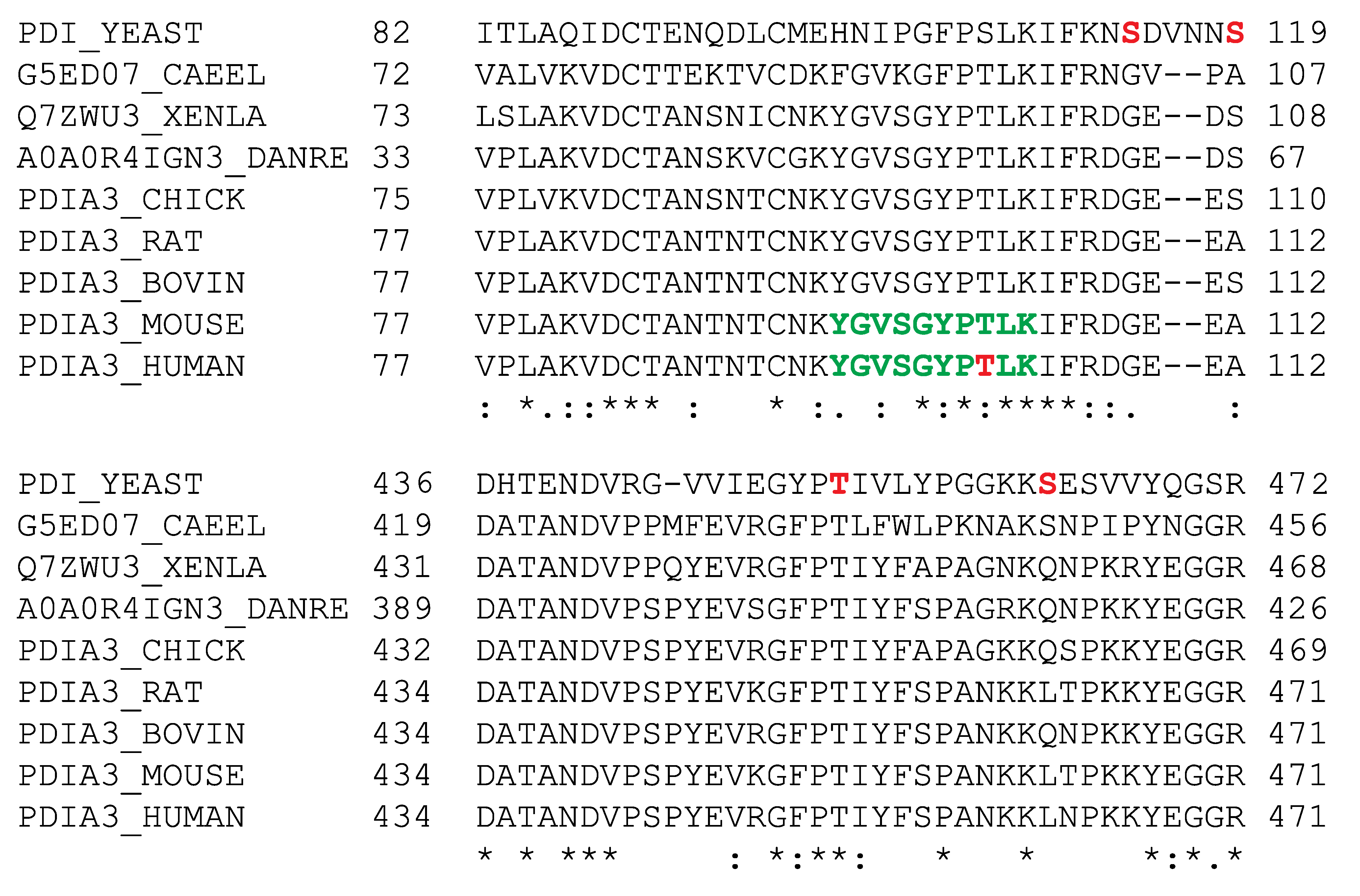

Supplement: S10 Fig — Alignment of sequences for PDIA3 protein or respective homologues from different species including, from top to bottom, Saccharomyces cerevisiae, Caenorhabditis elegans, Xenopus laevis, Danio rerio, Gallus gallus, Rattus norvegicus, Bos taurus, Mus musculus and Homo sapiens. The sequence is an extract from both Thioredoxin domains. Similarity is indicated by “.”, high similarity by “:” and identical amino acids by” *”. Identified glycopeptides from mouse brain (this study) and human breast cancer cells [31] are depicted in bold green letters. Bold red letters indicate O-mannosylated serine or threonine residues. (TIF) [file pone.0166119.s010.tif]

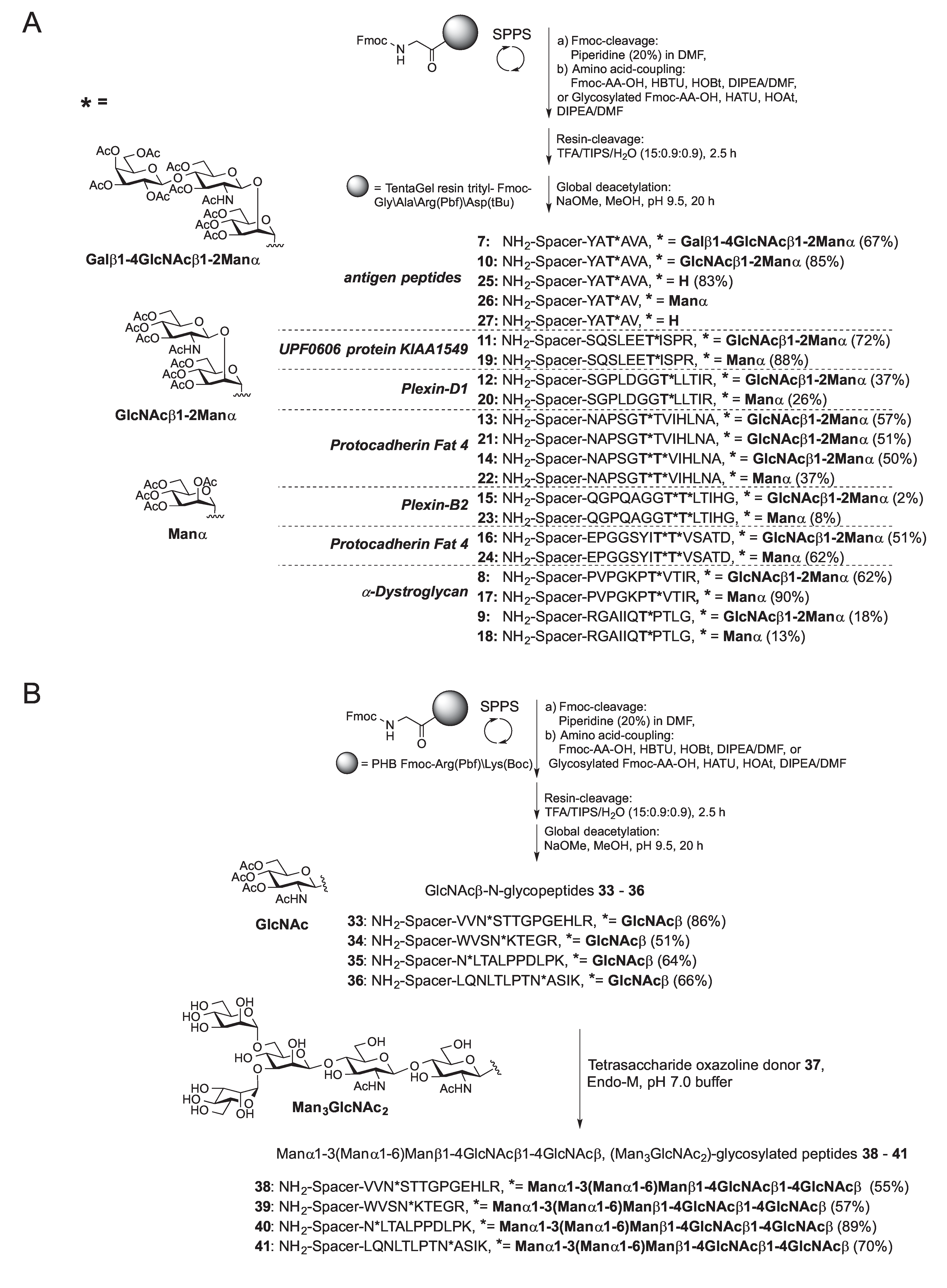

Supplement: S11 Fig — A) An overview of the Fmoc-Solid-phase peptide synthesis of the O-mannosyl glycopeptides. B) An overview of the Fmoc-Solid-phase peptide synthesis of the N-glycopeptides. Synthesis yields are given in parenthesis. (TIF) [file pone.0166119.s011.tif]
