# Supplementary material for: Protein O-Mannosylation in the Murine Brain: Occurrence of Mono-O-Mannosyl Glycans and Identification of New Substrates
Source: PLoS One. 2016 Nov 3;11(11):e0166119. doi: 10.1371/journal.pone.0166119 (PMC5094735; doi:10.1371/journal.pone.0166119)

| Raw file | Scan | Method | Score | m/z | Gene names |
|---|---|---|---|---|---|
| V-E-616 | 7178 | FTMS; HCD | 64.45 | 558.24 | Gm6483;Cdh11 |

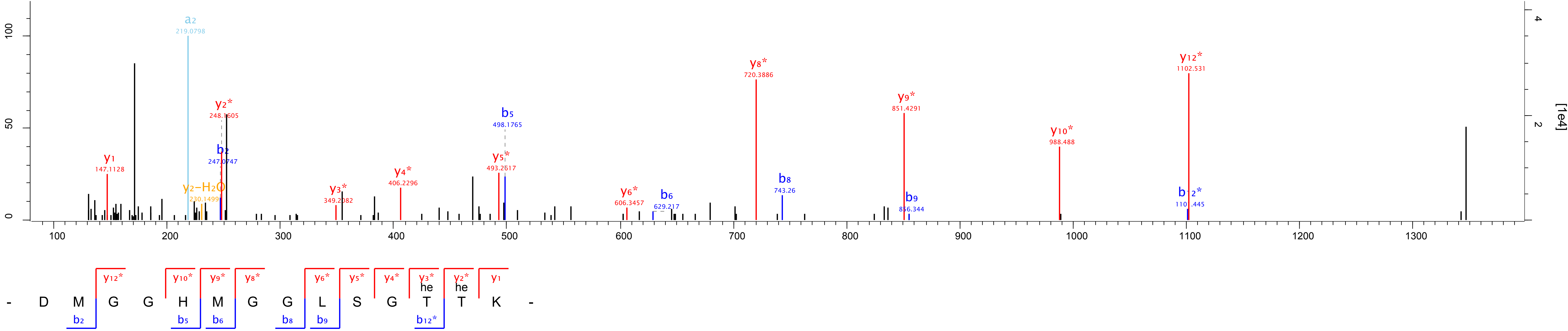

| Raw file | Scan | Method | Score | m/z | Gene names |
|---|---|---|---|---|---|
| V-E-953 | 10721 | FTMS; HCD | 156.78 | 1141.53 | Cdh13 |

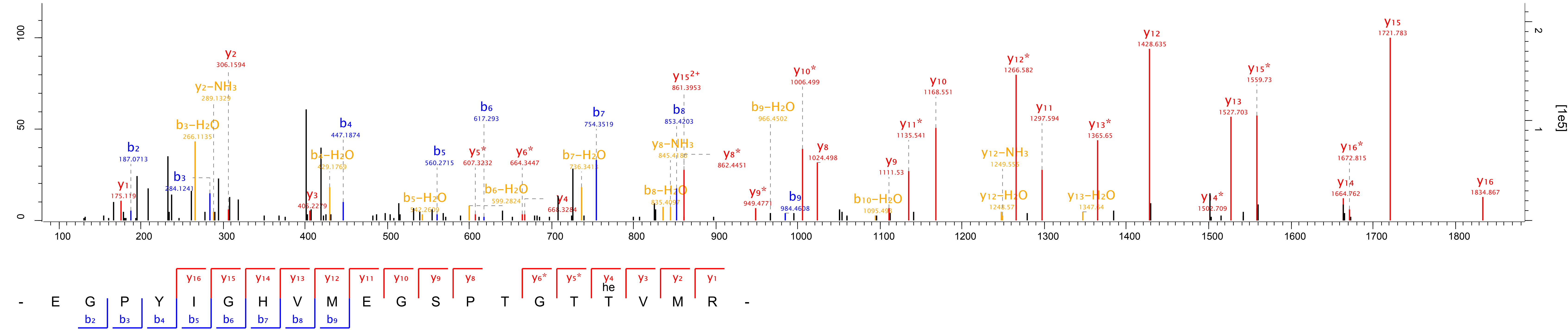

| Raw file | Scan | Method | Score | m/z | Gene names |
|---|---|---|---|---|---|
| V-E-616 | 10586 | FTMS; HCD | 100.24 | 839.03 | Fat1 |

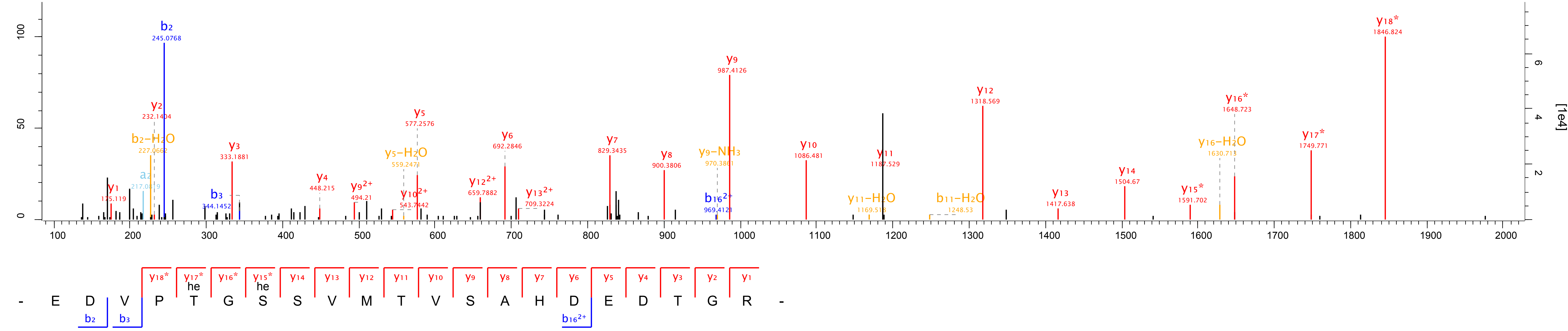

| Raw file | Scan | Method | Score | m/z | Gene names |
| --- | --- | --- | --- | --- | --- |
| 14-398-10 | 7672 | FTMS; HCD | 46.42 | 977.97 | Fat3 |

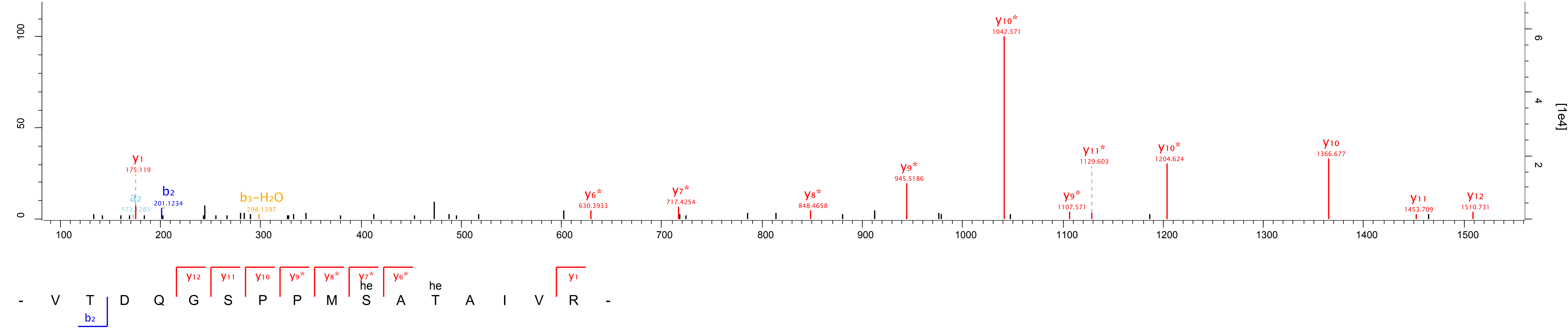

| Raw file | Scan | Method | Score | m/z | Gene names |
|---|---|---|---|---|---|
| V-E-953 | 6488 | FTMS; HCD | 48.57 | 582.82 | Itih5 |

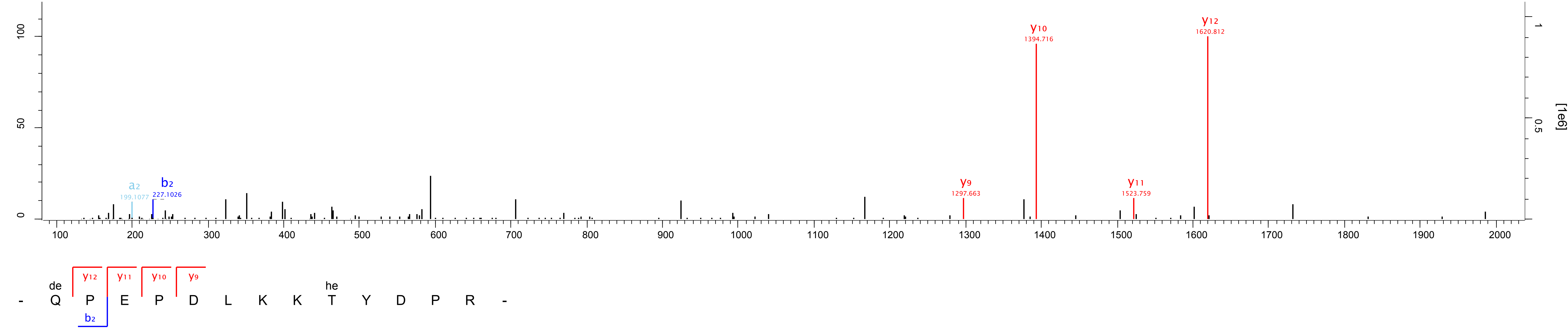

| Raw file | Scan | Method | Score | m/z | Gene names |
| --- | --- | --- | --- | --- | --- |
| V-E-615 | 4838 | FTMS; HCD | 41.51 | 766.01 | Ncan |

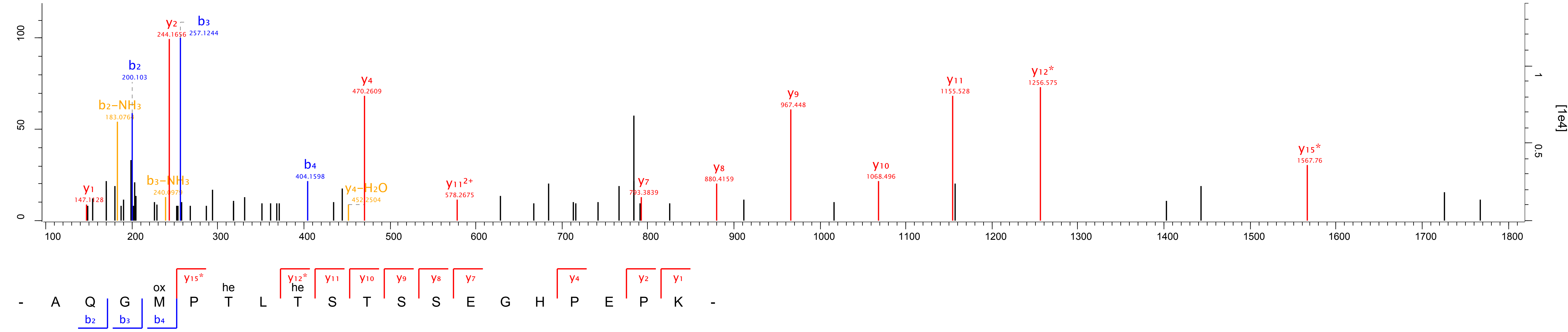

| Raw file | Scan | Method | Score | m/z | Gene names |
| --- | --- | --- | --- | --- | --- |
| V-E-924 | 7984 | FTMS; HCD | 58.81 | 764.39 | Nrxn3 |

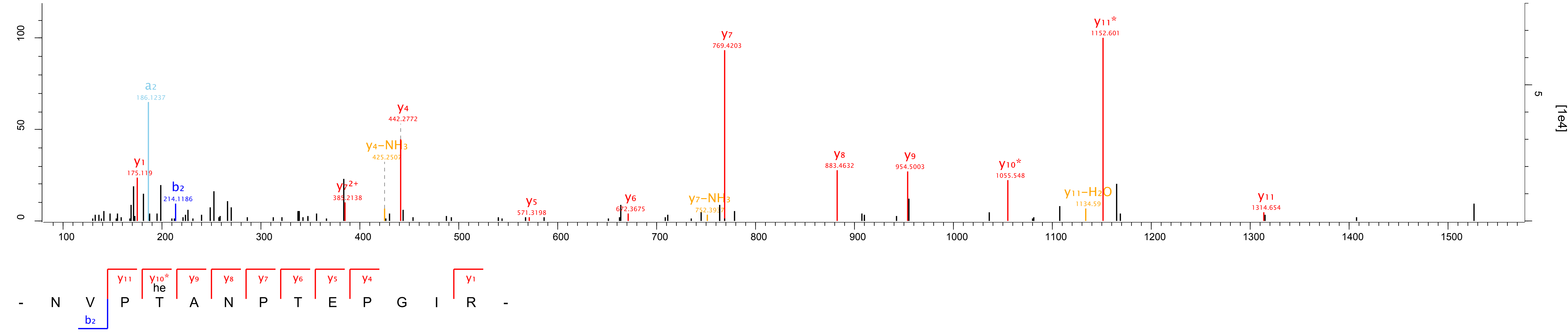

| Raw file | Scan | Method | Score | m/z | Gene names |
|---|---|---|---|---|---|
| V-E-952 | 8120 | FTMS; HCD | 100.04 | 635.34 | Pcdh10 |

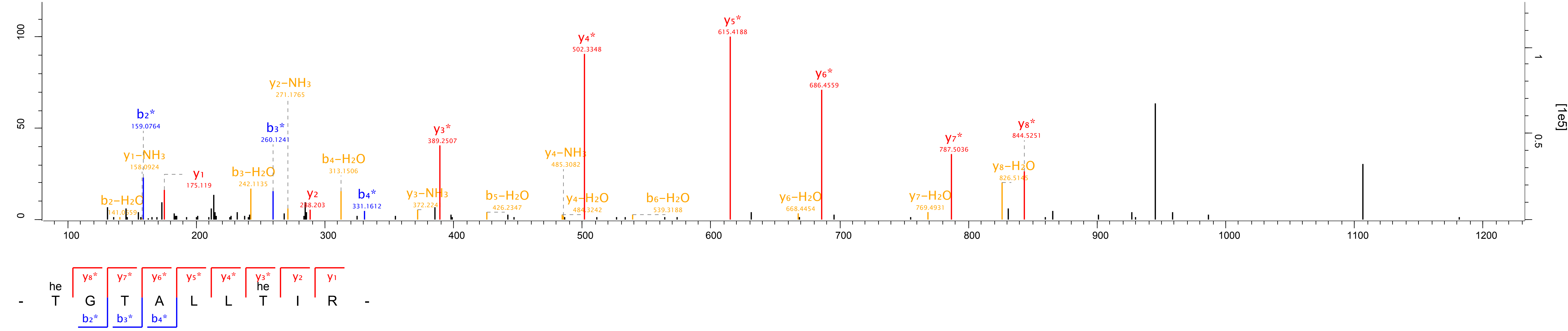

| Raw file | Scan | Method | Score | m/z | Gene names |
|---|---|---|---|---|---|
| V-E-960 | 9721 | FTMS; HCD | 76.89 | 1112.01 | Pcdh9 |

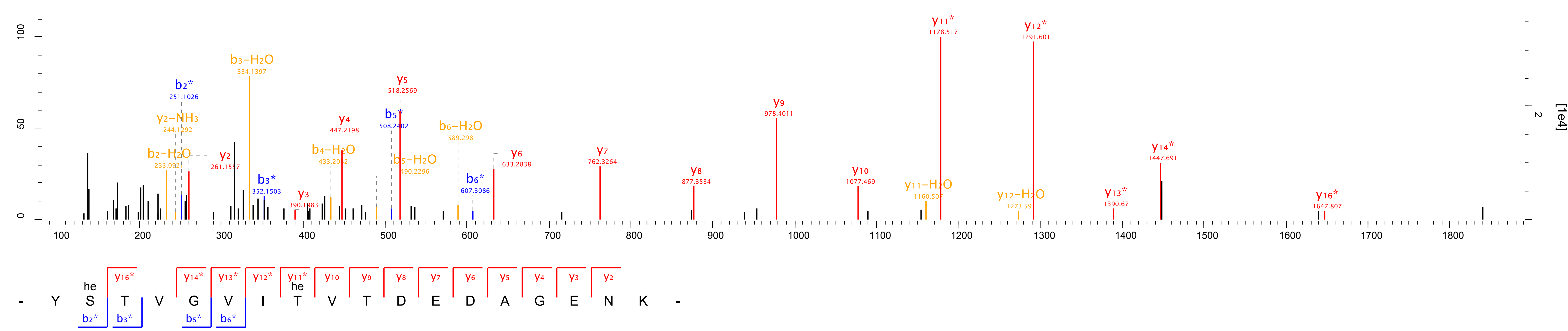

| Raw file | Scan | Method | Score | m/z | Gene names |
| --- | --- | --- | --- | --- | --- |
| V-E-924 | 8031 | FTMS; HCD | 87.68 | 869.37 | Pcdhac2 |

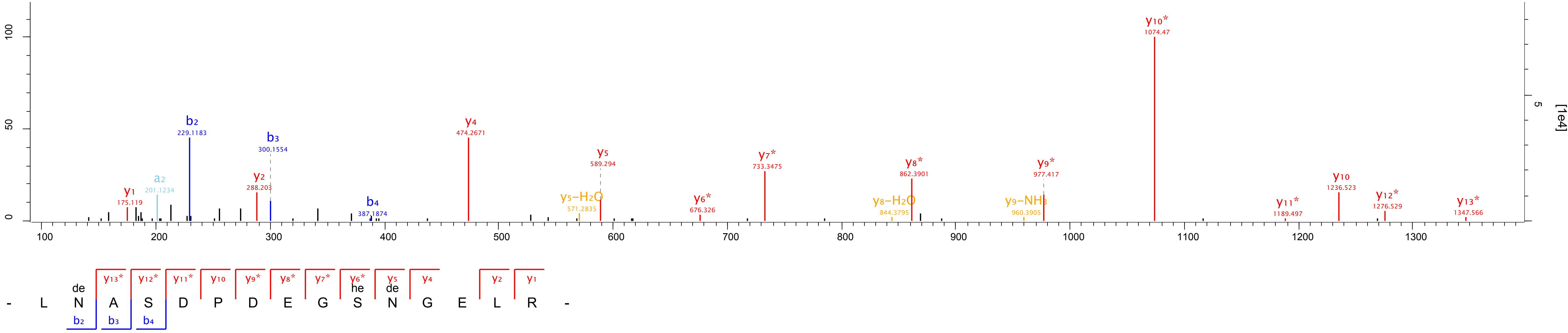

| Raw file | Scan | Method | Score | m/z | Gene names |
|---|---|---|---|---|---|
| V-E-615 | 5723 | FTMS; HCD | 85.4 | 948.43 | Pcdhga12 |

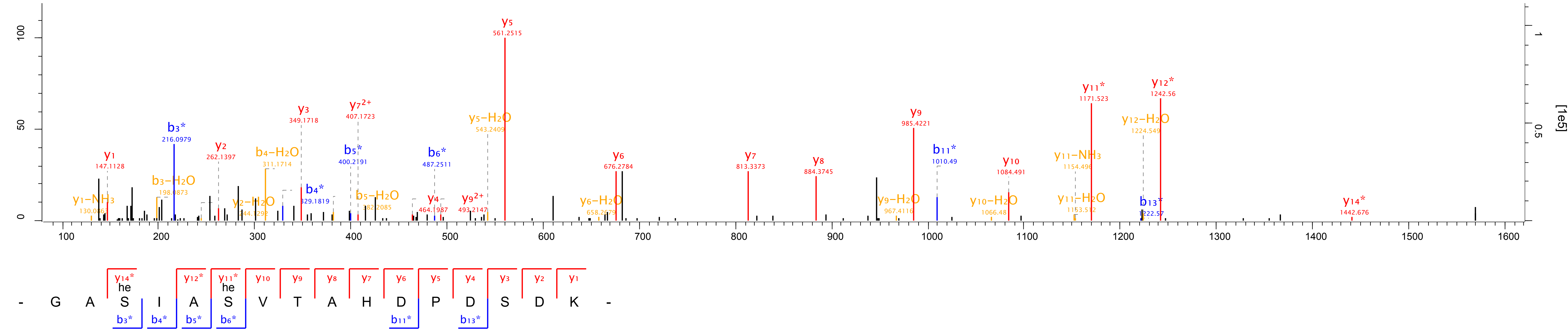

| Raw file | Scan | Method | Score | m/z | Gene names |
|---|---|---|---|---|---|
| V-E-963 | 10096 | FTMS; HCD | 68.48 | 777.92 | Pcdhgb5 |

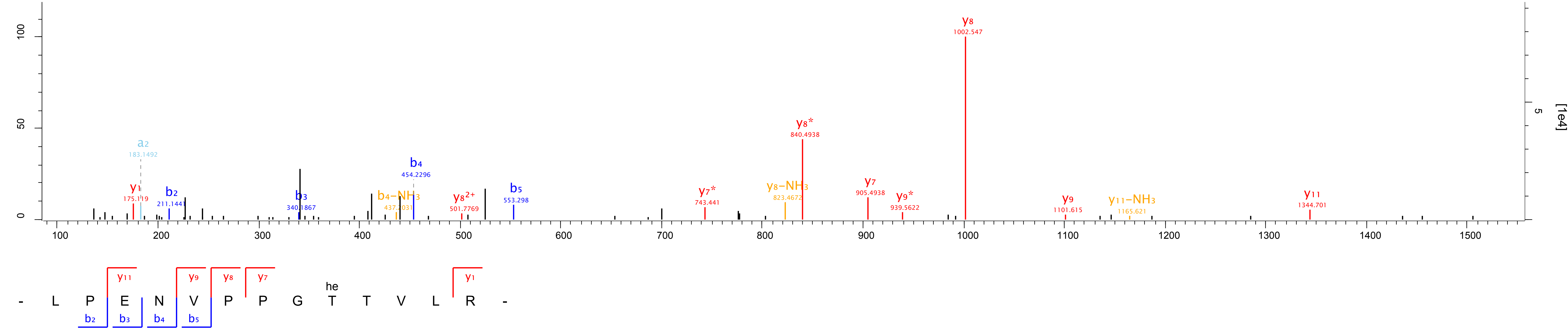

| Raw file | Scan | Method | Score | m/z | Gene names |
| --- | --- | --- | --- | --- | --- |
| V-E-957 | 8383 | FTMS; HCD | 88.02 | 623.81 | Pdia3 |

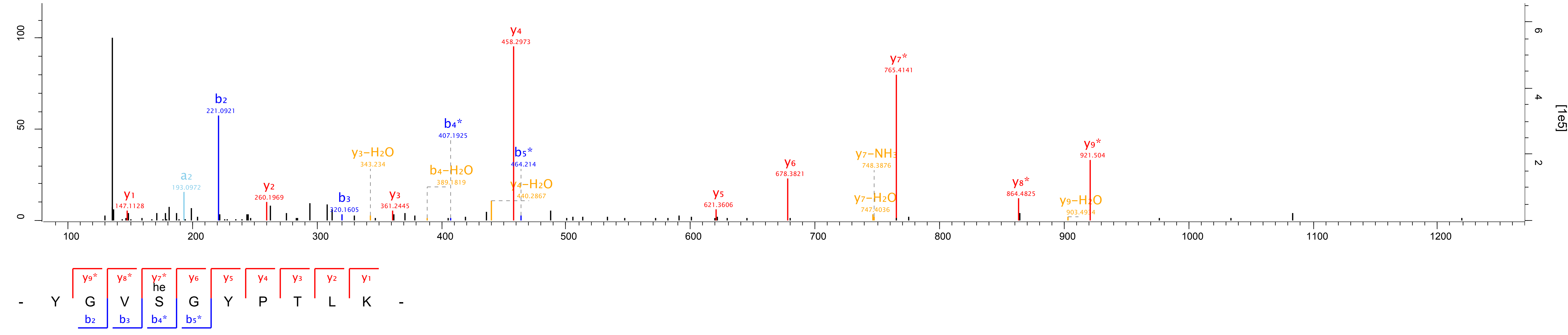

| Raw file | Scan | Method | Score | m/z | Gene names |
|---|---|---|---|---|---|
| V-E-963 | 11949 | FTMS; HCD | 50.9 | 674.87 | Plxna1 |

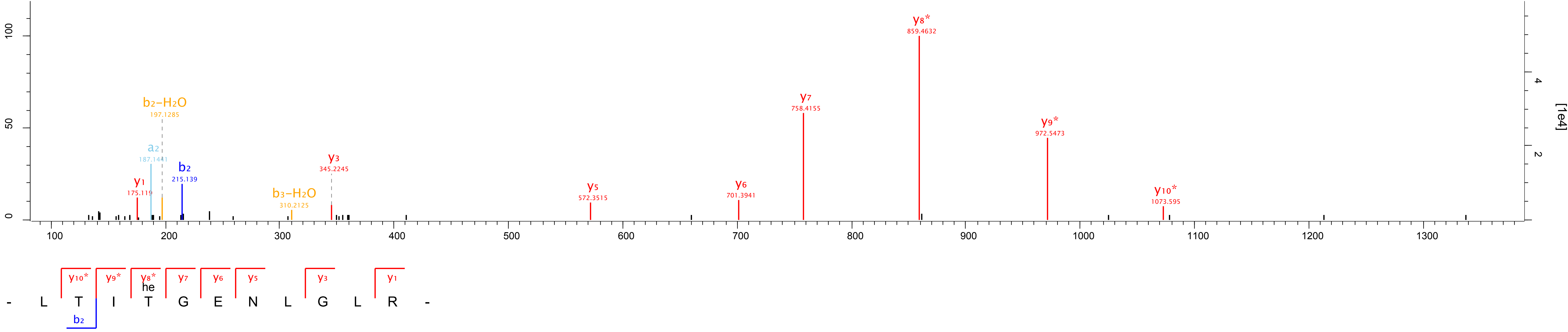

| Raw file | Scan | Method | Score | m/z | Gene names |
|---|---|---|---|---|---|
| V-E-615 | 11438 | FTMS; HCD | 62.85 | 773.04 | Plxnb2 |

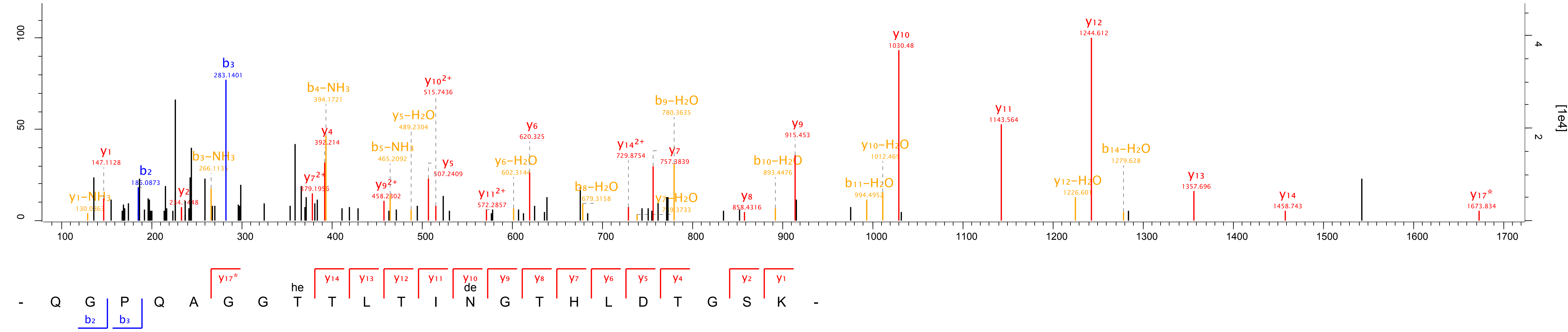

| Raw file | Scan | Method | Score | m/z | Gene names |
| --- | --- | --- | --- | --- | --- |
| V-E-616 | 6483 | FTMS; HCD | 66.15 | 665.3 | Ptprz1 |

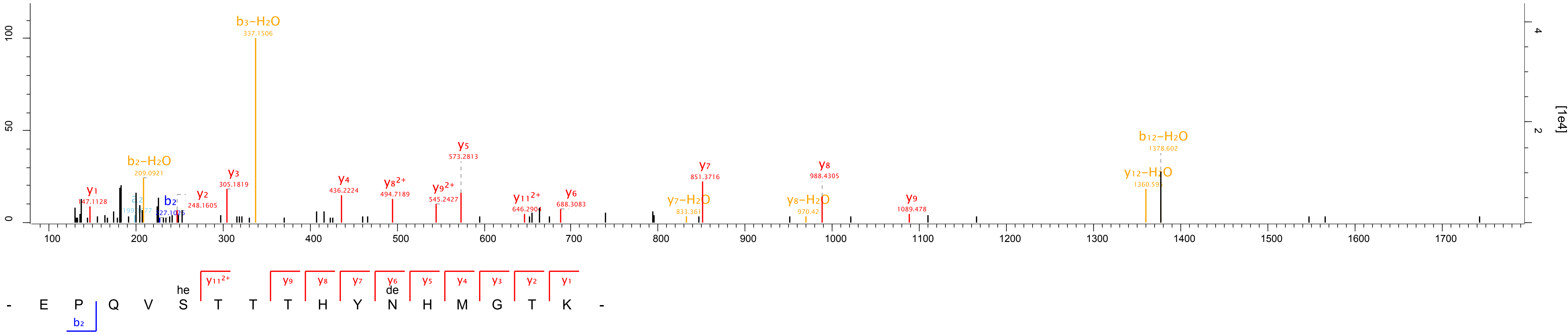

| Raw file | Scan | Method | Score | m/z | Gene names |
|---|---|---|---|---|---|
| V-E-615 | 6375 | FTMS; HCD | 89.48 | 861.39 | Ptprz1 |

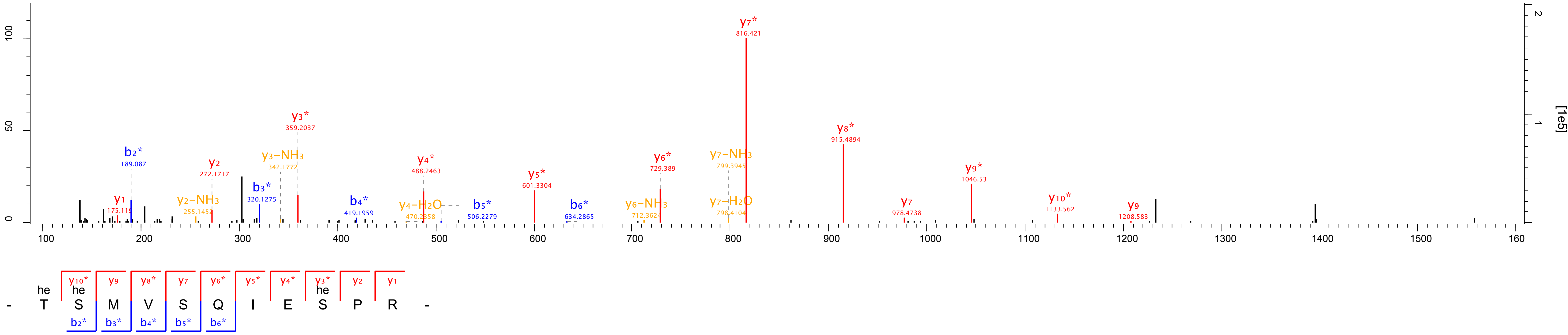

| Raw file | Scan | Method | Score | m/z | Gene names |
| --- | --- | --- | --- | --- | --- |
| V-E-615 | 7204 | FTMS; HCD | 46.86 | 876.03 | Ptprz1 |

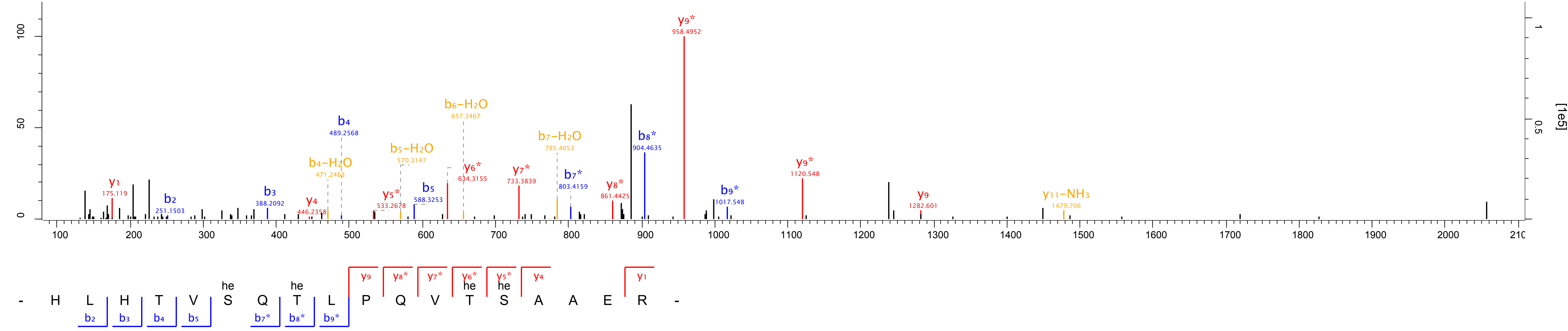

| Raw file | Scan | Method | Score | m/z | Gene names |
| --- | --- | --- | --- | --- | --- |
| V–E–616 | 12197 | FTMS; HCD | 40.62 | 866.39 | Ptprz1 |

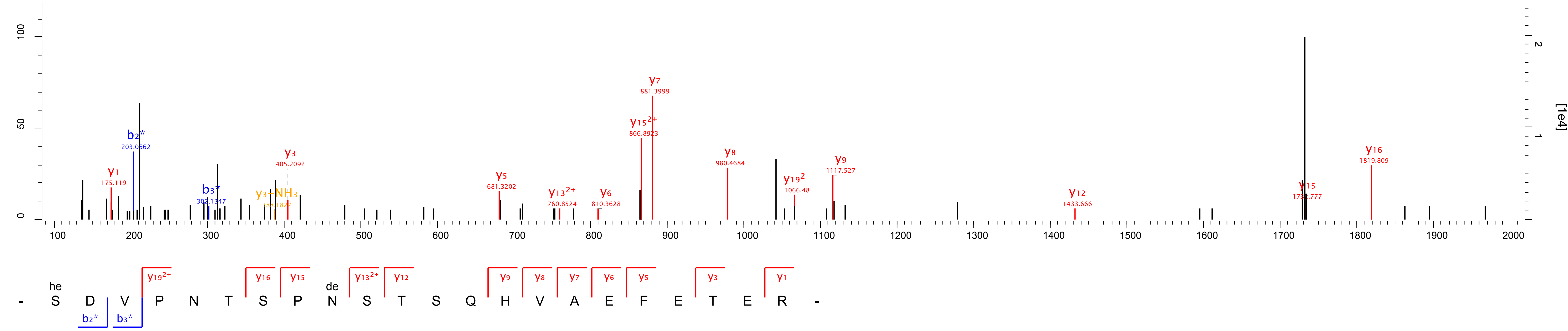

| Raw file | Scan | Method | Score | m/z | Gene names |
| --- | --- | --- | --- | --- | --- |
| V-E-959 | 9194 | FTMS; HCD | 70.2 | 756.81 | Ptprz1 |

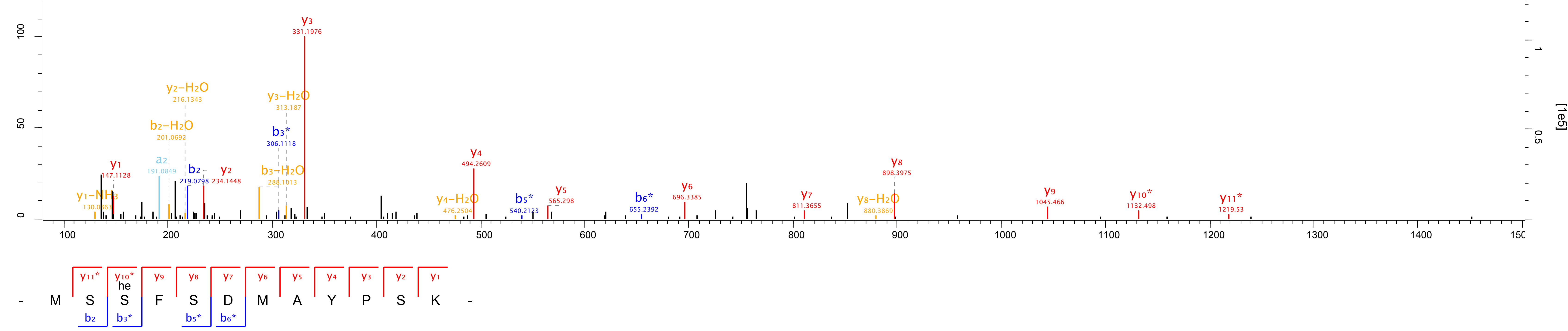

| Raw file | Scan | Method | Score | m/z | Gene names |
|---|---|---|---|---|---|
| 14-398-10 | 6885 | FTMS; HCD | 77.18 | 596.84 | Ptprz1 |

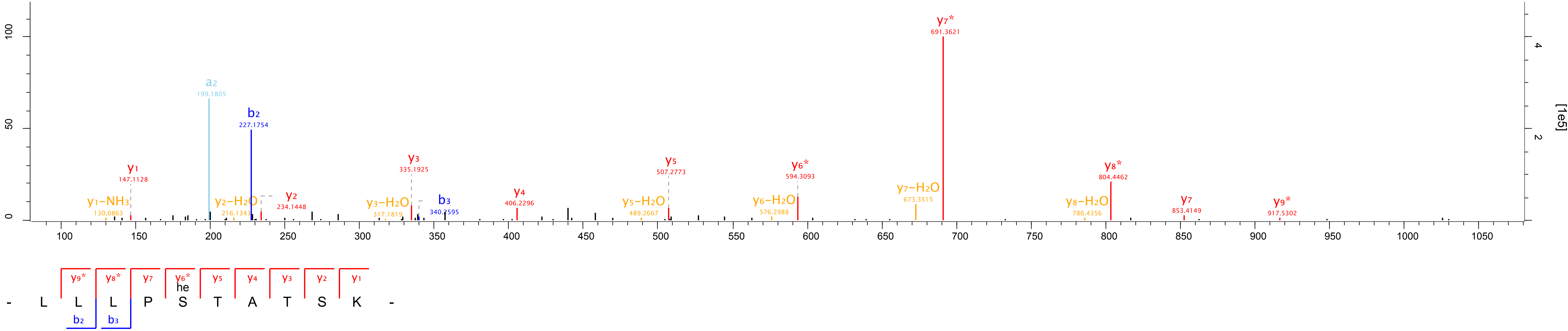

Supplement: S6 Fig — Glycopeptides identified in HCD-MS/MS analysis. Individual HCD fragment spectra/patterns of all identified glycopeptides are given. y-fragments are colored red and b-fragments are colored blue. Peptide sequence and the corresponding fragment patterns are depicted below each spectrum. Deamidation at asparagine is indicated by “de”. Presence of glycosylation was determined by neutral loss of hexoses from the precursor ion mass. Localization of glycosylation can be ambiguously mapped to serine and threonine residues. Position of hexose indicator “he” is based on the suggestion by MaxQuant. (PDF) [file pone.0166119.s006.pdf]
