# Supplementary material for: Protein O-Mannosylation in the Murine Brain: Occurrence of Mono-O-Mannosyl Glycans and Identification of New Substrates
Source: PLoS One. 2016 Nov 3;11(11):e0166119. doi: 10.1371/journal.pone.0166119 (PMC5094735; doi:10.1371/journal.pone.0166119)
Supplement: S1 File — (DOCX) [file pone.0166119.s012.docx]

**Supplementary Methods I**

**Cultivation and staining of cultured cells.**

Madin-Darby canine kidney (MDCK) cells (ATCC CRL-2936) were cultured on Nunc Lab-Tek II (Sigma; S6690) cover slides for 4 days in DMEM including 10 % FBS, 50 u Penicillin, 50 µg Streptomycin, 1 mM sodium pyruvate and 1x MEM non-essential amino acids (all from Invitrogen), in the presence of 37.5 µM R3A-5a inhibitor dissolved in DMSO or DMSO only. Cells were fixed with 4 % (wt/vol) paraformaldehyde dissolved in phosphorous buffered saline (PBS) for 10 min at 4°C. After permeabilization with 1 % Triton-X-100 for 5 minutes at 4 °C, cells were washed two times with PBS containing 0.025 % (vol/vol) Tween-20 (PBS/T) and blocked for 1 h in 2 % (wt/vol) BSA in PBS/T (PBS/T+BSA). Primary antibodies (polyclonal anti-T[α1-man] and monoclonal anti-O-Man antibodies enriched from hybridoma culture supernatant through Protein A chromatography) were applied in a 1:25 dilution in PBS/T+BSA overnight at 4 °C. Secondary antibodies (1:1000 in PBS/T+BSA; goat-anti-rabbit AlexaFluor488; Molecular Probes) were applied for 1 h after washing with PBS/T. Nuclei were counterstained with 0.3 µM DAPI for 3 min and cells were embedded in mounting medium (H-1000, Vectashield).

**Purification and pre-adsorption of hPOMT2 antibody.**

Essentially α-POMT2 antibodies were produced as described before [1]. In brief, amino acids 373 to 470 of human POMT2 were recombinantly expressed in *E. coli* BL21 cells. Enrichment of the antibody from serum was achieved using a method by Olmsted *et al* [2], for which the recombinant protein was transferred to nitrocellulose after SDS-PA gel electrophoresis, incubated with serum and eluted using a pH shift. For preadsorption this eluate was again incubated with nitrocellulose-fixed recombinant protein overnight and the supernatant employed for standard immunofluorescent detection of mouse WT brain cryosections as stated in the Materials and Methods section.

**Synthesis of glycosylated Fmoc-amino acid building blocks.**

*N*-9-Fluorenylmethoxycarbonyl-*O*-[2,3,4,6-*O-*acetyl-α-D-mannopyranosyl]-L-threonine-*tert*-butylester (3)

The glycosylated amino acid **3** was here prepared by an improved coupling strategy using a trichloroacetimidate donor instead of previous low performance couplings using the acetate as donor [3]. The trichloroacetimidate donor **1** [4] (2.98 g, 6.04 mmol, 1.2 eq) and the protected amino acid **2** [5, 6] (2.00 g, 5.04 mmol, 1.0 eq) was dissolved in dry Et_2_O (40 mL) and 4 Å molecular sieves (2.0 g) was added. The suspension was stirred under argon at room temperature (r.t.) for 30 min and trimethylsilyl -trifluoromethanesulfonate (TMSOTf) (182 μL, 1.0 mmol, 0.2 eq) was added. The reaction was kept stirring at r.t. for 30 min, then quenched with triethylamine (200 μL). The molecular sieves were removed by filtering over a layer of *Celite*. The filtrate was concentrated and purified by column chromatography (cyclohexane-ethyl acetate 10:1 to 3:1) to give **3** (2.69 g, 3.70 mmol, 73%). R*_f_* = 0.5 (^C^Hex/EtOAc 2:1). *HR-ESI-TOF-MS* (Thermo LTQ-Orbitrap mass spectrometer), *m/z*: 728.2912 ([M+H]^+^, calc. 728.2918), 745.3185 ([M+NH_4_]^+^, calc. 745.3182), 750.2738 ([M+Na]^+^, calc. 750.2738). ^1^H NMR (400 MHz, CDCl_3_) δ = 7.77 (d, *J*=7.5, 2H, Fmoc), 7.65 (d, *J*=7.4, 2H, Fmoc), 7.43 – 7.35 (m, 2H, Fmoc), 7.33 (td, *J*=7.3, 3.8, 2H, Fmoc), 5.55 (d, *J*=9.4, 1H, N*H*), 5.32 (dd, *J*=10.0, 3.2, 1H, H3), 5.25 (t, *J*=9.9, 1H, H4), 5.17 (s, 1H, H2), 4.92 (s, 1H, H1), 4.46 – 4.37 (m, 2H, Fmoc C*H*_2_), 4.37 – 4.30 (m, 2H, Thr C*H*β, Thr C*H*α), 4.30 – 4.21 (m, 2H, Fmoc C*H*, H6a), 4.15 – 4.10 (m, 1H, H6b), 4.10 – 4.04 (m, 1H, H5), 2.14 (s, 3H, Ac), 2.08 (s, 3H, Ac), 2.06 (s, 3H, Ac), 1.99 (s, 3H, Ac), 1.48 (s, 9H, tBu), 1.33 (d, *J*=6.3, 3H, ThrC*H*_3_). ^13^C NMR (101 MHz, CDCl_3_) δ = 170.6, 169.9, 169.8, 169.0 (170.6 - 169.0 Ac and Fmoc *C*=O), 156.7 (Thr *C*=O), 143.9, 141.4, 127.8, 127.2, 125.3, 125.3, 120.1, 120.1 (143.9 - 120.1 Fmoc aromatic), 99.0 (C1), 83.1 (*C*(CH_3_)_3_), 77.7 (Thr Cβ), 69.6 (C2), 69.1 (C5), 69.00 (C3), 67.5 (Fmoc *C*H_2_), 66.4 (C4), 62.7 (C6), 59.0 (Thr Cα), 47.2 (Fmoc C*H*), 28.0 (C(*C*H_3_)_3_), 20.9, 20.8, 20.8, 20.7 (20.9 - 20.7 Ac *C*H_3_), 18.1 (Thr *C*H_3_).

*N*-9-Fluorenylmethoxycarbonyl-*O*-[2,3,4,6-*O-*acetyl-α-D-mannopyranosyl]-L-threonine (4) [3]

Compound **3** (2.65 g, 3.64 mmol) was dissolved in TFA/DCM 3:1 (20 mL) and was stirred at r.t. for 0.5 hours. The solution was then concentrated and coevaporated three times with toluene, purified by column chromatography (DCM-MeOH 100:1 to 10:1) to give **4** (2.31 g, 3.45 mmol, 95%). R*_f_* = 0.3 (MeOH/DCM 1:20). *HR-ESI-TOF-MS* (Thermo LTQ-Orbitrap mass spectrometer), *m/z*: 672.2283 ([M+H]^+^, calc. 672.2292), 689.2558 ([M+NH_4_]^+^, calc. 689.2558), 694.2114 ([M+Na]^+^, calc. 694.2114).  ^1^H NMR (500 MHz, cdcl_3_) δ = 7.76 (d, *J*=7.5, 2H, Fmoc), 7.67 – 7.60 (m, 2H, Fmoc), 7.39 (td, *J*=7.4, 3.8, 2H, Fmoc), 7.35 – 7.29 (m, 2H, Fmoc), 6.06 (d, *J*=9.6, 1H, N*H*), 5.35 (dd, *J*=10.0, 3.4, 1H, H3), 5.26 (t, *J*=9.9, 1H, H4), 5.07 (dd, *J*=3.2, 1.7, 1H, H2), 4.96 (d, *J*=1.0, 1H, H1), 4.54 (dd, *J*=9.6, 2.4, 1H, Thr C*H*α), 4.46 – 4.36 (m, 3H, Thr C*H*β, Fmoc C*H*_2_), 4.26 (dd, *J*=12.1, 5.6, 2H, Fmoc C*H*, H6a), 4.14 – 4.09 (m, 1H, H6b), 4.09 – 4.06 (m, 1H, H5), 2.11 (s, 3H, Ac), 2.08 (s, 3H, Ac), 2.04 (s, 3H, Ac), 2.00 (s, 3H, Ac), 1.32 (d, *J*=6.4, 3H, ThrC*H*_3_). ^13^C NMR (126 MHz, cdcl_3_) δ = 172.4, 170.8, 170.8, 170.5, 169.9 (172.4 - 169.9 Ac and Fmoc *C*=O), 156.9 (Thr *C*=O), 144.0, 143.8, 141.4, 141.4, 127.8, 127.2, 125.3, 125.3, 120.1 (144.0 - 120.1 Fmoc aromatic), 98.9 (C1), 77.9 (Thr Cβ), 69.9 (C2), 69.1 (C3, C5), 67.5 (Fmoc *C*H_2_), 66.3 (C4), 62.6 (C6), 58.5 (Thr Cα), 47.2 (Fmoc C*H*), 20.9, 20.9, 20.8, 20.8 (20.9 - 20.8 Ac *C*H_3_), 18.0 (Thr *C*H_3_).

# General procedure: Synthesis of *O*-mannosyl glycopeptides.

The *O-*Mannosyl peptides **7** - **25** were synthesized by stepwise solid-phase peptide synthesis using the Fmoc strategy and starting with preloaded tentagel R Trt Fmoc-Gly, Fmoc-Ala, Fmoc-Arg(Pbf) or Fmoc-Asp(*t*Bu) resin (12.5 μmol scale). The glycopeptide synthesis performed using 1.5 eq of the protected Galβ1-4GlcNAcβ1-2Manα trisaccharide- (**5**) [7] or GlcNAcβ1-2Manα disaccharide- (**6**) [7] or Manα monosaccharide- (**4**) Fmoc amino acids, which were pre-activated manually using 1.5 eq HATU/HOAt and 3.0 eq of DIPEA (8 h coupling time). The standard Fmoc amino acids were coupled automatically on a Multisyntech peptide synthesizer using 8 eq of the amino acid and the HBTU/HOBt reagents and 16 eq of DIPEA (40 min). Fmoc deprotection was done according to standard conditions, 20% piperidine in DMF. After assembly of the peptide backbone, a triethyleneglycol amino acid spacer [8, 9] was coupled to the *N*-terminus followed by Fmoc deprotection. The obtained peptides were then released from the resin, and all acid sensitive side-chain protecting groups were simultaneously removed using TFA/TIPS/H_2_O 15:0.9:0.9 for 2.5 h followed by solvent concentration, lyophilization and purification using a C-18 cartridge (1 g of C-18 material, Waters, Eschborn, Germany). For saccharide deprotection, the *O-*acetyl groups were cleaved by transesterification in methanol using catalytic amounts of NaOMe at pH 9-9.5 for 24 h (deprotection monitored by analytical HPLC) to yield glycopeptides **7** - **25** which were purified by preparative HPLC.

Compound 7, Sequence: NH_2_-Spacer-YAT*AVA, *=Galβ1-4GlcNAcβ1-2Manα.

Yield: 67% (11.15 mg, 8.41 μmol). Analytical HPLC Rt= 15.31 min (Phenomenex Luna C18 (2), 3μm, 2.0 x 150 mm, Grad: MeCN (84%)/water + 0.1% FA (5:95)->(50:50), 45 min, wavelength = 214 nm). Preparative HPLC Rt= 11.29 min (Phenomenex Luna C18 (2), 10 μm, 21.2x250mm, Grad: MeCN (84%)/water + 0.1% TFA (5:95)->(55:45), 25 min, wavelength=214 nm); *HR-ESI-TOF-MS* (Thermo LTQ-Orbitrap mass spectrometer), *m/z*: 663.3080 ([M+2H]^2+^, calc. 663.3089), 671.8132 ([M+H+NH_4_]^2+^, calc. 671.8222), 674.3009 ([M+Na+H]^2+^, calc. 674.2999).

Compound 8, Sequence: NH_2_-Spacer-PVPGKPT*VTIR, *= GlcNAcβ1-2Manα.

Yield: 62% (15.5 mg, 7.8 μmol). Analytical HPLC Rt= 22.25 min (Phenomenex Luna C18 (2), 2.0 x 150 mm, 3 μm, Grad: MeCN/water + 0.1% FA (5:95)->(45:55), 40 min, wavelength=214 nm). Preparative HPLC Rt= 12.53 min (Phenomenex Luna C18 (2), 10 μm, 21.2x250mm, Grad: MeCN (84%)/water + 0.1% TFA (5:95)->(55:45), 25 min, wavelength=214 nm); *HR-ESI-TOF-MS* (Thermo LTQ-Orbitrap mass spectrometer), *m/z*: 866.9857 ([M+2H]^2+^, calc. 866.9872), 877.9768 ([M+H+Na]^2+^, calc. 877.9782), 578.3239 ([M+3H]^3+^, calc. 578.3274).

Compound 9, Sequence: NH_2_-Spacer-RGAIIQT*PTLG, *= GlcNAcβ1-2Manα.

Yield: 18% (4.3 mg, 2.2 μmol). Analytical HPLC Rt= 25.35 min (Phenomenex Luna C18 (2), 2.0 x 150 mm, 3 μm, Grad: MeCN/water + 0.1% FA (5:95)->(45:55), 40 min, wavelength=214 nm). Preparative HPLC Rt= 14.45 min (Phenomenex Luna C18 (2), 10 μm, 21.2x250mm, Grad: MeCN (84%)/water + 0.1% TFA (5:95)->(55:45), 25 min, wavelength=214 nm); *HR-ESI-TOF-MS* (Thermo LTQ-Orbitrap mass spectrometer), *m/z*: 1694.9332 ([M+H]^+^, calc. 1694.9064), 847.9578 ([M+2H]^2+^, calc. 847.9571), 578.6265 ([M+H+Na+NH_4_]^3+^, calc. 578.6435).

Compound 10, Sequence: NH_2_-Spacer-YAT*AVA, *= GlcNAcβ1-2Manα.

Yield: 85% (12.37 mg, 10.63 μmol). Analytical HPLC Rt= 15.58 min (Phenomenex Luna C18 (2), 3μm, 2.0 x 150 mm, Grad: MeCN (84%)/water + 0.1% FA (5:95)->(50:50), 45 min, wavelength = 214 nm). Preparative HPLC Rt= 11.57 min (Phenomenex Luna C18 (2), 10 μm, 21.2x250mm, Grad: MeCN (84%)/water + 0.1% TFA (5:95)->(55:45), 25 min, wavelength=214 nm); HR-ESI-TOF-MS (Thermo LTQ-Orbitrap mass spectrometer), *m/z*: 1185.5462 ([M+Na]^+^, calc. 1185.5391), 582.2816 ([M+2H]^2+^, calc. 582.2825), 593.2743 ([M+H+Na]^2+^, calc. 593.2735).

Compound 11, Sequence: NH_2_-Spacer-SQSLEET*ISPR, *= GlcNAcβ1-2Manα.

Yield: 72% (16.24 mg, 8.95 μmol). Analytical HPLC Rt= 14.75 min (Phenomenex Luna C18 (2), 3μm, 2.0 x 150 mm, Grad: MeCN (84%)/water + 0.1% FA (5:95)->(50:50), 45 min, wavelength = 214 nm). Preparative HPLC Rt= 11.33 min (Phenomenex Luna C18 (2), 10 μm, 21.2x250mm, Grad: MeCN (84%)/water + 0.1% TFA (5:95)->(55:45), 25 min, wavelength=214 nm); HR-ESI-TOF-MS (Thermo LTQ-Orbitrap mass spectrometer), *m/z*: 907.9411 ([M+2H]^2+^, calc. 907.9459), 918.9360 ([M+H+Na]^2+^, calc. 918.9369), 605.6315 ([M+3H]^3+^, calc. 605.6332), 454.4798 ([M+4H]^4+^, calc. 454.4769).

Compound 12, Sequence: NH_2_-Spacer-SGPLDGGT*LLTIR, *= GlcNAc-Man.

Yield: 37% (8.65 mg, 4.63 μmol). Analytical HPLC Rt= 23.11 min (Phenomenex Luna C18 (2), 3μm, 2.0 x 150 mm, Grad: MeCN (84%)/water + 0.1% FA (5:95)->(50:50), 45 min, wavelength = 214 nm). Preparative HPLC Rt= 16.12 min (Phenomenex Luna C18 (2), 10 μm, 21.2x250mm, Grad: MeCN (84%)/water + 0.1% TFA (5:95)->(55:45), 25 min, wavelength=214 nm); HR-ESI-TOF-MS (Thermo LTQ-Orbitrap mass spectrometer), *m/z*: 934.4905 ([M+2H]^2+^, calc. 934.4915), 945.4853 ([M+H+Na]^2+^, calc. 945.4825), 623.3311 ([M+3H]^3+^, calc. 623.3303).

Compound 13, Sequence: NH_2_-Spacer-NAPSGT*TVIHLNA, *= GlcNAcβ1-2Manα.

Yield: 57% (13.34 mg, 7.16 μmol). Analytical HPLC Rt= 17.64 min (Phenomenex Luna C18 (2), 3μm, 2.0 x 150 mm, Grad: MeCN (84%)/water + 0.1% FA (5:95)->(50:50), 45 min, wavelength = 214 nm). Preparative HPLC Rt= 13.62 min (Phenomenex Luna C18 (2), 10 μm, 21.2x250mm, Grad: MeCN (84%)/water + 0.1% TFA (5:95)->(55:45), 25 min, wavelength=214 nm); HR-ESI-TOF-MS (Thermo LTQ-Orbitrap mass spectrometer), *m/z*: 931.9649 ([M+2H]^2+^, calc. 931.9657), 942.9597 ([M+H+Na]^2+^, calc. 942.9667), 621.6474 ([M+3H]^3+^, calc. 621.6464).

Compound 14, Sequence: NH_2_-Spacer-NAPSGT*T*VIHLNA, *= GlcNAc-Man.

Yield: 50% (14.04 mg, 6.30 μmol). Analytical HPLC Rt= 16.41 min (Phenomenex Luna C18 (2), 3μm, 2.0 x 150 mm, Grad: MeCN (84%)/water + 0.1% FA (5:95)->(50:50), 45 min, wavelength = 214 nm). Preparative HPLC Rt= 12.36 min (Phenomenex Luna C18 (2), 10 μm, 21.2x250mm, Grad: MeCN (84%)/water + 0.1% TFA (5:95)->(55:45), 25 min, wavelength=214 nm); HR-ESI-TOF-MS (Thermo LTQ-Orbitrap mass spectrometer), *m/z*: 1114.5318 ([M+2H]^2+^, calc. 1114.5318), 1125.5267 ([M+H+Na]^2+^, calc. 1125.5228), 743.3582 ([M+3H]^3+^, calc. 743.3571).

Compound 15, Sequence: NH_2_-Spacer-QGPQAGGT*T*LTIHG, *= GlcNAcβ1-2Manα.

Yield: 2% (0.61 mg, 0.27 μmol). Analytical HPLC Rt= 15.04 min (Phenomenex Luna C18 (2), 3μm, 2.0 x 150 mm, Grad: MeCN (84%)/water + 0.1% FA (5:95)->(50:50), 45 min, wavelength = 214 nm). Preparative HPLC Rt= 11.27 min (Phenomenex Luna C18 (2), 10 μm, 21.2x250mm, Grad: MeCN (84%)/water + 0.1% TFA (5:95)->(55:45), 25 min, wavelength=214 nm); HR-ESI-TOF-MS (Thermo LTQ-Orbitrap mass spectrometer), *m/z*: 1136.5385 ([M+2H]^2+^, calc. 1136.5363), 1147.5302 ([M+H+Na]^2+^, calc. 1147.5273), 758.0279 ([M+3H]^3+^, calc. 758.0268).

Compound 16, Sequence: NH_2_-Spacer-EPGGSYIT*T*VSATD, *= GlcNAcβ1-2Manα.

Yield: 51% (14.79 mg, 6.34 μmol). Analytical HPLC Rt= 17.55 min (Phenomenex Luna C18 (2), 3μm, 2.0 x 150 mm, Grad: MeCN (84%)/water + 0.1% FA (5:95)->(50:50), 45 min, wavelength = 214 nm). Preparative HPLC Rt= 11.45 min (Phenomenex Luna C18 (2), 10 μm, 21.2x250mm, Grad: MeCN (84%)/water + 0.1% TFA (5:95)->(55:45), 25 min, wavelength=214 nm); HR-ESI-TOF-MS (Thermo LTQ-Orbitrap mass spectrometer), *m/z*: 1166.5179 ([M+2H]^2+^, calc. 1166.5175), 1177.5146 ([M+H+Na]^2+^, calc. 1177.5085), 778.0169 ([M+3H]^3+^, calc. 778.0142).

Compound 17, Sequence: NH_2_-Spacer-PVPGKPT*VTIR, *= Manα.

Yield: 90% (17.16 mg, 11.22 μmol). Analytical HPLC Rt= 14.36 min (Phenomenex Luna C18 (2), 3μm, 2.0 x 150 mm, Grad: MeCN (84%)/water + 0.1% FA (5:95)->(50:50), 45 min, wavelength = 214 nm). Preparative HPLC Rt= 12.46 min (Phenomenex Luna C18 (2), 10 μm, 21.2x250mm, Grad: MeCN (84%)/water + 0.1% TFA (5:95)->(55:45), 25 min, wavelength=214 nm); HR-ESI-TOF-MS (Thermo LTQ-Orbitrap mass spectrometer), *m/z*: 1529.8820 ([M+H]^+^, calc. 1529.8871), 765.4428 ([M+2H]^2+^, calc. 765.4475), 510.6306 ([M+3H]^3+^, calc. 510.6342).

Compound 18, Sequence: NH_2_-Spacer-RGAIIQT*PTLG, *= Manα.

Yield: 13% (2.38 mg, 1.60 μmol). Analytical HPLC Rt= 18.90 min (Phenomenex Luna C18 (2), 3μm, 2.0 x 150 mm, Grad: MeCN (84%)/water + 0.1% FA (5:95)->(50:50), 45 min, wavelength = 214 nm). Preparative HPLC Rt= 14.46 min (Phenomenex Luna C18 (2), 10 μm, 21.2x250mm, Grad: MeCN (84%)/water + 0.1% TFA (5:95)->(55:45), 25 min, wavelength=214 nm); HR-ESI-TOF-MS (Thermo LTQ-Orbitrap mass spectrometer), *m/z*: 1491.8346 ([M+H]^+^, calc. 1491.8270), 746.4165 ([M+2H]^2+^, calc. 746.7174), 757.4101 ([M+H+Na]^2+^, calc. 757.4084).

Compound 19, Sequence: NH_2_-Spacer- SQSLEET*ISPR, *= Manα.

Yield: 88% (11.30 mg, 11.02 μmol). Analytical HPLC Rt= 15.20 min (Phenomenex Luna C18 (2), 3μm, 2.0 x 150 mm, Grad: MeCN (84%)/water + 0.1% FA (5:95)->(50:50), 45 min, wavelength = 214 nm). Preparative HPLC Rt= 11.86 min (Phenomenex Luna C18 (2), 10 μm, 21.2x250mm, Grad: MeCN (84%)/water + 0.1% TFA (5:95)->(55:45), 25 min, wavelength=214 nm); HR-ESI-TOF-MS (Thermo LTQ-Orbitrap mass spectrometer), *m/z*: 806.4014 ([M+2H]^2+^, calc. 806.4062), 817.3959 ([M+H+Na]^2+^, calc. 817.3972), 537.9383 ([M+3H]^3+^, calc. 537.9401).

Compound 20, Sequence: NH_2_-Spacer-SGPLDGGT*LLTIR, *= Manα.

Yield: 26% (5.51 mg, 3.31 μmol). Analytical HPLC Rt= 23.59 min (Phenomenex Luna C18 (2), 3μm, 2.0 x 150 mm, Grad: MeCN (84%)/water + 0.1% FA (5:95)->(50:50), 45 min, wavelength = 214 nm). Preparative HPLC Rt= 16.64 min (Phenomenex Luna C18 (2), 10 μm, 21.2x250mm, Grad: MeCN (84%)/water + 0.1% TFA (5:95)->(55:45), 25 min, wavelength=214 nm); HR-ESI-TOF-MS (Thermo LTQ-Orbitrap mass spectrometer), *m/z*: 832.9509 ([M+2H]^2+^, calc. 832.9518), 843.9451 ([M+H+Na]^2+^, calc. 843.9428), 555.6380 ([M+3H]^3+^, calc. 555.6371).

Compound 21, Sequence: NH_2_-Spacer-NAPSGT*TVIHLNA, *= Manα.

Yield: 51% (10.54 mg, 6.35 μmol). Analytical HPLC Rt= 18.19 min (Phenomenex Luna C18 (2), 3μm, 2.0 x 150 mm, Grad: MeCN (84%)/water + 0.1% FA (5:95)->(50:50), 45 min, wavelength = 214 nm). Preparative HPLC Rt= 13.93 min (Phenomenex Luna C18 (2), 10 μm, 21.2x250mm, Grad: MeCN (84%)/water + 0.1% TFA (5:95)->(55:45), 25 min, wavelength=214 nm); HR-ESI-TOF-MS (Thermo LTQ-Orbitrap mass spectrometer), *m/z*: 830.4252 ([M+2H]^2+^, calc. 830.4260), 841.4199 ([M+H+Na]^2+^, calc. 841.4170), 553.9542 ([M+3H]^3+^, calc. 553.9532).

Compound 22, Sequence: NH_2_-Spacer-NAPSGT*T*VIHLNA, *= Manα.

Yield: 37% (8.45 mg, 4.64 μmol). Analytical HPLC Rt= 16.89 min (Phenomenex Luna C18 (2), 3μm, 2.0 x 150 mm, Grad: MeCN (84%)/water + 0.1% FA (5:95)->(50:50), 45 min, wavelength = 214 nm). Preparative HPLC Rt= 13.05 min (Phenomenex Luna C18 (2), 10 μm, 21.2x250mm, Grad: MeCN (84%)/water + 0.1% TFA (5:95)->(55:45), 25 min, wavelength=214 nm); HR-ESI-TOF-MS (Thermo LTQ-Orbitrap mass spectrometer), *m/z*: 911.4513 ([M+2H]^2+^, calc. 911.4524), 922.4462 ([M+H+Na]^2+^, calc. 922.4434), 607.9718 ([M+3H]^3+^, calc. 607.9708).

Compound 23, Sequence: NH_2_-Spacer-QGPQAGGT*T*LTIHG, *= Manα.

Yield: 8% (1.86 mg, 1.00 μmol). Analytical HPLC Rt= 15.45 min (Phenomenex Luna C18 (2), 3μm, 2.0 x 150 mm, Grad: MeCN (84%)/water + 0.1% FA (5:95)->(50:50), 45 min, wavelength = 214 nm). Preparative HPLC Rt= 11.94 min (Phenomenex Luna C18 (2), 10 μm, 21.2x250mm, Grad: MeCN (84%)/water + 0.1% TFA (5:95)->(55:45), 25 min, wavelength=214 nm); HR-ESI-TOF-MS (Thermo LTQ-Orbitrap mass spectrometer), *m/z*: 932.9547 ([M+2H]^2+^, calc. 932.9553), 943.9487 ([M+H+Na]^2+^, calc. 943.9463), 622.3069 ([M+3H]^3+^, calc. 622.3061).

Compound 24, Sequence: NH_2_-Spacer-EPGGSYIT*T*VSATD, *=Manα.

Yield: 62% (15.00 mg, 7.79 μmol). Analytical HPLC Rt= 18.81 min (Phenomenex Luna C18 (2), 3μm, 2.0 x 150 mm, Grad: MeCN (84%)/water + 0.1% FA (5:95)->(50:50), 45 min, wavelength = 214 nm). Preparative HPLC Rt= 12.42 min (Phenomenex Luna C18 (2), 10 μm, 21.2x250mm, Grad: MeCN (84%)/water + 0.1% TFA (5:95)->(55:45), 25 min, wavelength=214 nm); HR-ESI-TOF-MS (Thermo LTQ-Orbitrap mass spectrometer), *m/z*: 962.9354 ([M+2H]^2+^, calc. 962.9364), 971.4526 ([M+H+NH_4_]^2+^, calc. 971.4497), 973.9305 ([M+H+Na]^2+^, calc. 973.9274).

Compound 25, Sequence: NH_2_-Spacer-YATAVA.

Yield: 83% (8.25 mg, 10.43 μmol). Analytical HPLC Rt= 16.07 min (Phenomenex Luna C18 (2), 3μm, 2.0 x 150 mm, Grad: MeCN (84%)/water + 0.1% FA (5:95)->(50:50), 45 min, wavelength = 214 nm). Preparative HPLC Rt= 12.47 min (Phenomenex Luna C18 (2), 10 μm, 21.2x250mm, Grad: MeCN (84%)/water + 0.1% TFA (5:95)->(55:45), 25 min, wavelength=214 nm); HR-ESI-TOF-MS (Thermo LTQ-Orbitrap mass spectrometer), *m/z*: 798.4230 ([M+H]^+^, calc. 798.4249).

Compound 26, Sequence: NH_2_ -YAT*AV, *= Manα.

The antigen peptide was produced in contract with Epitomics Inc (Woerdern, Austria).

Compound 27, Sequence: NH_2_- YATAV.

The unglycosylated antigen peptide was produced in contract with Epitomics Inc (Woerdern, Austria).

Compound 28 - 31

The syntheses were described in a previous publication [7].

# General procedure: Synthesis of *N*-glycopeptides.

The *N*-glycopeptides **33** - **36** were synthesized by stepwise solid-phase peptide synthesis using the Fmoc strategy and starting with preloaded PHB Fmoc-Arg (Pbf) resin or tentagel PHB Fmoc-Lys (Boc) resin (12.5 μmol scale). The glycopeptide synthesis performed using 1.5 eq of the protected *N*-glycosylated building block **32** [10], which was pre-activated manually using 1.5 eq HATU/HOAt and 3.0 eq of DIPEA (8 h coupling time). The standard Fmoc amino acids were coupled automatically on a Multisyntech peptide synthesizer using 8 eq of the amino acid, 8 eq of the HBTU/HOBt reagents and 16 eq of DIPEA (40 min). Fmoc deprotection was done according to standard conditions, 20% piperidine in DMF. After the assembly of the peptide backbone, a triethyleneglycol amino acid spacer was coupling to the *N*-terminus and then Fmoc deprotection was performed. The obtained peptides were then released from the resin, and all acid sensitive side-chain protecting groups were simultaneously removed using TFA/TIPS/H_2_O 15:0.9:0.9 for 3 h, followed by solvent concentration, lyophilization and purification using a C-18 cartridge (1 g of C-18 material, Waters). For saccharide deprotection, the *O-*acetyl groups were removed by transesterification in methanol using catalytic amounts of NaOMe at pH 9-9.5 for 24 h (the deprotection monitored by analytical HPLC) to yield glycopeptides **33** - **36** which were purified by preparative HPLC.

Compound 33, Sequence: NH_2_-Spacer-VVN*STTGPGEHLR, *= GlcNAcβ. [11]

Compound **33** is previously reported [11]. Yield: 86% (19.0 mg, 10.70 μmol). Analytical HPLC Rt= 21.13 min (Phenomenex Luna C18 (2), 2.0 x 150 mm, 3 μm, Grad: MeCN/water + 0.1% TFA (5:95)->(45:55), 40 min, wavelength=214 nm). Preparative HPLC Rt= 11.71 min (Phenomenex Luna C18 (2), 10 μm, 21.2x250mm, Grad: MeCN (84%)/water + 0.1% TFA (5:95)->(55:45), 25 min, wavelength=214 nm); *HR-ESI-TOF-MS* (Thermo LTQ-Orbitrap mass spectrometer), *m/z*: 886.9564 ([M+2H]^2+^, calc. 886.9554), 897.9493 ([M+Na+H]^2+^, calc. 897.9564), 591.6386 ([M+3H]^3+^, calc. 591.6395), 599.3019 ([M+Na+2H]^3+^, calc. 599.3013), 604.6232 ([M+Na+NH_4_+H]^3+^, calc. 604.6424).

Compound 34, Sequence: NH_2_-Spacer-WVSN*KTEGR, *= GlcNAcβ. [11]

Compound **34** is previously reported [11]. Yield: 51% (9.43 mg, 6.36 μmol). Analytical HPLC Rt= 20.62 min (Phenomenex Luna C18 (2), 2.0 x 150 mm, 3 μm, Grad: MeCN/water + 0.1% TFA (5:95)->(45:55), 40 min, wavelength=214 nm). Preparative HPLC Rt= 11.80 min (Phenomenex Luna C18 (2), 10 μm, 21.2x250mm, Grad: MeCN (84%)/water + 0.1% TFA (5:95)->(55:45), 25 min, wavelength=214 nm); *HR-ESI-TOF-MS* (Thermo LTQ-Orbitrap mass spectrometer), *m/z*: 1482.7529 ([M+H]^+^, calc. 1482.7440), 741.8753 ([M+2H]^2+^, calc. 741.8759), 752.8684 ([M+Na+H]^2+^, calc. 752.8669), 494.9190 ([M+3H]^3+^, calc. 494.9199), 507.9036 ([M+Na+NH_4_+H]^3+^, calc. 507.9227).

Compound 35, Sequence: NH_2_-Spacer-N*LTALPPDLPK, *= GlcNAcβ.

Yield: 64% (12.7 mg, 8.02 μmol). Analytical HPLC Rt= 28.87 min (Phenomenex Luna C18 (2), 2.0 x 150 mm, 3 μm, Grad: MeCN/water + 0.1% TFA (5:95)->(45:55), 40 min, wavelength=214 nm). Preparative HPLC Rt= 15.35 min (Phenomenex Luna C18 (2), 10 μm, 21.2x250mm, Grad: MeCN (84%)/water + 0.1% TFA (5:95)->(55:45), 25 min, wavelength=214 nm); *HR-ESI-TOF-MS* (Thermo LTQ-Orbitrap mass spectrometer), *m/z*: 1584.8936 ([M+H]^+^, calc. 1584.8736), 792.9407 ([M+2H]^2+^, calc. 792.9407), 803.9349 ([M+Na+H]^2+^, calc. 803.9317), 528.9644 ([M+3H]^3+^, calc. 528.9631), 541.9481 ([M+Na+NH_4_+H]^3+^, calc. 541.9659), 396.9659 ([M+4H]^4+^, calc. 396.9743).

Compound 36, Sequence: NH_2_-Spacer-LQNLTLPTN*ASIK, *= GlcNAcβ.

Yield: 66% (15.0 mg, 9.43 μmol). Analytical HPLC Rt= 30.57 min (Phenomenex Luna C18 (2), 2.0 x 150 mm, 3 μm, Grad: MeCN/water + 0.1% TFA (5:95)->(45:55), 40 min, wavelength=214 nm). Preparative HPLC Rt= 16.05 min (Phenomenex Luna C18 (2), 10 μm, 21.2x250mm, Grad: MeCN (84%)/water + 0.1% TFA (5:95)->(55:45), 25 min, wavelength=214 nm); *HR-ESI-TOF-MS* (Thermo LTQ-Orbitrap mass spectrometer), *m/z*: 1819.0355 ([M+H]^+^, calc. 1819.0064), 910.0078 ([M+2H]^2+^, calc. 910.0071), 921.0024 ([M+Na+H]^2+^, calc. 920.9981), 607.0099 ([M+3H]^3+^, calc. 607.0073), 614.6719 ([M+Na+2H]^3+^, calc. 614.6691), 619.9933 ([M+Na+NH_4_+H]^3+^, calc. 620.0102).

# Enzymatic coupling of a oxazoline donor to generate pentasaccharide core *N*-glycopeptides.

Compound 38, Sequence: NH_2_-Spacer-VVN*STTGPGEHLR, *= Manα1-3(Manα1-6)Manβ1-4GlcNAcβ1-4GlcNAcβ, (Man_3_GlcNAc_2_). [11]

Compound **38** is previously reported [11]. Yield 55% (0.32 mg, 0.13 µmol). Analytical HPLC Rt= 15.36 min (Phenomenex Luna C18 (2), 2.0 x 150 mm, 3 μm, Grad: MeCN/water + 0.1% TFA (10:90)->(40:60), 30 min, wavelength=214 nm). Semi-preparative HPLC Rt= 15.50 min (Thermo Hypersil GOLD, 5 μm, 4.6x250mm, Grad: MeCN (84%)/water + 0.1% TFA (10:90)->(40:60), 30 min, wavelength=214 nm); *HR-ESI-TOF-MS* (Thermo LTQ-Orbitrap mass spectrometer), *m/z*: 1232.0969 ([M+2H]^2+^, calc. 1232.0761), 821.7212 ([M+3H]^3+^, calc. 821.7200), 616.5465 ([M+4H]^4+^, calc. 616.5419), 501.0277 ([M+K+4H]^5+^, calc. 501.0263).

Compound 39, Sequence: NH_2_-Spacer-WVSN*KTEGR, *= Manα1-3(Manα1-6)Manβ1-4GlcNAcβ1-4GlcNAcβ, (Man_3_GlcNAc_2_). [11]

Compound **39** is previously reported [11]. Yield 57% (0.35 mg, 0.16 µmol). Analytical HPLC Rt= 13.83 min (Phenomenex Luna C18 (2), 2.0 x 150 mm, 3 μm, Grad: MeCN/water + 0.1% TFA (10:90)->(40:60), 30 min, wavelength=214 nm). Semi-preparative HPLC Rt= 14.63 min (Thermo Hypersil GOLD, 5 μm, 4.6x250mm, Grad: MeCN (84%)/water + 0.1% TFA (10:90)->(40:60), 30 min, wavelength=214 nm); *HR-ESI-TOF-MS* (Thermo LTQ-Orbitrap mass spectrometer), *m/z*: 1086.5079 ([M+2H]^2+^, calc. 1086.4949), 724.6675 ([M+3H]^3+^, calc. 724.6658), 553.2415 ([M+K+3H]^4+^, calc. 553.2403).

Compound 40, Sequence: NH_2_-Spacer-N*LTALPPDLPK, *= Manα1-3(Manα1-6)Manβ1-4GlcNAcβ1-4GlcNAcβ, (Man_3_GlcNAc_2_).

The tetrasaccharide oxazoline donor **37** [12, 13] (1.19 mg, 1.73 µmol, 8.2 eq) and the GlcNAc-peptide **35** (0.42 mg, 0.21 µmol 1.0 eq) were dissolved in phosphate buffer (50 mM, pH 7.1, 7.75 µL) and an enzyme solution of Endo-M-N175Q (TCI chemistry, 12.5 milli-units in 6.25 µL buffer) was then added. After incubation for 1h at 25 °C, analytical HPLC showed that a faster product was obtained with full conversion and the reaction was finished. The crude product was diluted with H_2_O and purified by semi-prep HPLC directly to give glycopeptide **40**, yield 89% (0.42 mg, 0.18 µmol). Analytical HPLC Rt= 21.99 min (Phenomenex Luna C18 (2), 2.0 x 150 mm, 3 μm, Grad: MeCN/water + 0.1% TFA (10:90)->(40:60), 30 min, wavelength=214 nm). Semi-preparative HPLC Rt= 22.41 min (Thermo Hypersil GOLD, 5 μm, 4.6x250mm, Grad: MeCN (84%)/water + 0.1% TFA (10:90)->(40:60), 30 min, wavelength=214 nm); *HR-ESI-TOF-MS* (Thermo LTQ-Orbitrap mass spectrometer), *m/z*: 1138.0721 ([M+2H]^2+^, calc. 1138.0614), 759.0502 ([M+3H]^3+^, calc. 759.0435), 579.0296 ([M+K+3H]^4+^, calc. 579.0236).

Compound 41, Sequence: NH_2_-Spacer-LQNLTLPTN*ASIK, *= Manα1-3(Manα1-6)Manβ1-4GlcNAcβ1-4GlcNAcβ, (Man_3_GlcNAc_2_)_._

The tetrasaccharide oxazoline donor **37** [12, 13] (1.03 mg, 1.49 µmol, 6.8 eq) and the GlcNAc-peptide **36** (0.40 mg, 0.22 µmol 1.0 eq) were dissolved in a phosphate buffer (50 mM, pH 7.1, 7.75 µL) and an enzyme solution, Endo-M-N175Q (TCI chemistry, 12.5 milli-units in 6.25 µL buffer), was added. After incubation for 1h at 25 °C, analytical HPLC showed that a faster product was obtained with full conversion and the reaction was finished. The crude product was diluted with H_2_O and purified by semi-prep HPLC directly to give glycopeptide **51**, yield 70% (0.35 mg, 0.15 µmol). Analytical HPLC Rt= 24.42 min (Phenomenex Luna C18 (2), 2.0 x 150 mm, 3 μm, Grad: MeCN/water + 0.1% TFA (10:90)->(40:60), 30 min, wavelength=214 nm). Semi-preparative HPLC Rt= 23.57 min (Thermo Hypersil GOLD, 5 μm, 4.6x250mm, Grad: MeCN (84%)/water + 0.1% TFA (10:90)->(40:60), 30 min, wavelength=214 nm); *HR-ESI-TOF-MS* (Thermo LTQ-Orbitrap mass spectrometer), *m/z*: 1255.1527 ([M+2H]^2+^, calc. 1255.1278), 837.0907 ([M+3H]^3+^, calc. 837.0878), 637.5616 ([M+K+3H]^4+^, calc. 637.5568).

For HPLC chromatograms of the individual compounds, please see S2 File.

**(Glyco)peptide microarray analysis of monoclonal antibody RKU-1-3-5**

# Antibody binding recognition of *O*-Mannosyl glycopeptides

The synthesized peptides **7** - **31** (Table S3) (0.25 mM of peptide in 150 mM phosphate buffer pH 8.5, 0.05% Tween-20, 85 picoL spots) were spotted and immobilized on amino reactive and biocompatible hydrogel slides with a surface functionalized with carboxylic acids activated as *N*-hydroxysuccinimide esters (Slide H, Schott-Nexterion, Jena, Germany). Using a piezo-driven non-contact spotter (iTWO200, M2-automation, Berlin, Germany) the spotting performed in an 8×1 array (well) format with 5 replicates of each glycopeptide in every block. The printed peptides were allowed to react in a humidity chamber (75% humidity) for 24 h and unreacted NHS-esters were capped with 1 mM ethanolamine at pH 9 for 1 h. The slides were washed with phosphate buffered saline (PBS), 0.05% Tween-20 buffer (PBST) and water and then dried with a stream of argon for storage or directly incubated with primary antibodies. The slides were incubated with the rabbit monoclonal antibody RKU-1-3-5 (diluted 1/1.5, 1/3 and 1/10 in PBST buffer from 0.39 μg/μL) for 1 h min and then washed 3 x 10 min with PBST buffer. For readout, the slides were incubated 1 h with a biotin labeled secondary anti-rabbit IgG antibody (Life technologies, diluted 1/2000 in PBST buffer from 2 mg/mL) and again washed 3 x 10 min with PBST buffer. Then the slides were incubated with Cy5 streptavidin for 30 min (Invitrogen, diluted 1/1000 in PBST buffer), washed 3 x 10 min with PBST buffer, water and slides were dried. The fluorescence was then detected on a Typhoon Trio^+^ scanner (General Electrics) and the signals were analyzed and quantified using the Image quant software. The mean and standard deviations of the spot replicates are presented in the graph (Figure S1 Fig and Table S3).

# *N*-glycopeptide microarrays for analysis of antibody binding specificity

The synthesized peptides **38** - **41** (0.1 mM of peptide in 150 mM phosphate buffer pH 8.5, 0.05% Tween-20, 85 picoL spots) were spotted and immobilized on amino reactive and biocompatible hydrogel slides with a surface functionalized with carboxylic acids activated as *N*-hydroxysuccinimide esters (Slide H, Schott-Nexterion, Jena, Germany). Using a piezo-driven non-contact spotter (iTWO200, M2-automation, Berlin, Germany) the spotting performed in an 8×2 array (well) format with 5 replicates for each glycopeptides in every block. The printed peptides were allowed to react in a humidity chamber (75% humidity) for 24 h and unreacted NHS-esters were trapped with 1 mM ethanolamine at pH 9 for 1 h. The slides were washed with phosphate buffered saline (PBS), 0.05% Tween-20 buffer (PBST) and water and then dried with a stream of argon for storage or directly incubated with primary antibodies. The slides were incubated with a) monoclonal antibody RKU-1-3-5 (diluted 1/3 in PBST buffer from 0.39 μg/μL) for 1 h or b) Biotin-ConA (0.1 mg/mL in PBST buffer) followed by washing 3 x 10 min with PBST buffer. For readout the slides were subsequently incubated a) 1 h with a biotin labeled secondary anti-rabbit IgG antibody (Life technologies, diluted 1/2000 in PBST buffer from 2 mg/mL), washed 3 x 10 min with PBST buffer, then incubation with Cy5 streptavidin for 30 min was followed (Invitrogen, diluted 1/1000 in PBST buffer) or in b) direct incubation with with Cy5 streptavidin for 30 min. The slides were washed 3 x 10 min with PBST buffer, water, and dried. Fluorescence was detected on a Typhoon Trio^+^ scanner (General Electrics) and the signals were analyzed and quantified using the Image quant software. The mean and standard deviations of the spot replicates are presented in the graph (Figure S4 Fig and Table S4).

**NGL-based microarray analyses**

For the screening analysis, the microarray (in-house designation ‘Glycosciences Array Sets 32-39’) containing 492 lipid-linked oligosaccharide probes (S3 File) was used. The probes were printed non-covalently in duplicate on nitrocellulose-coated glass slides at 2 and 5 fmol/spot [14]. This is an array validated with a wide spectrum of lectins and antibodies. Microarray analysis of the monoclonal antibody RKU-1-3-5 was performed essentially as described [15]. In brief, after blocking arrayed slides with 0.02% (v/v) Blocker Casein (Pierce), 1% (w/v) bovine serum albumin (Sigma A8577) in Hepes buffered saline (5 mM Hepes, pH 7.4, 150 mM NaCl, 5 mM CaCl2), RKU-1-3-5 was assayed at 1:3 dilution, followed by biotinylated anti-rabbit IgG (Sigma) at 1:200. As diluent the blocker solution was used. To detect binding Alexa Fluor-647-labeled streptavidin from Molecular Probes was used at 1 µg/ml. Data analysis and presentation was performed with dedicated glycan microarray software [16]. For analyses of RKU-1-3-5 at different dilutions (1:25, 1:50 and 1:100), a focused array that was named ‘N-glycan related Array Set 1’ containing 52 oligosaccharide probes was used.

**Supplementary Literature**

1. Willer T, Amselgruber W, Deutzmann R, Strahl S. Characterization of POMT2, a novel member of the PMT protein O-mannosyltransferase family specifically localized to the acrosome of mammalian spermatids. Glycobiology. 2002;12(11):771-783.

2. Olmsted JB. Affinity purification of antibodies from diazotized paper blots of heterogeneous protein samples. J Biol Chem. 1981;256(23):11955-11957.

3. Salvador LA, Elofsson M, Kihlberg J. Preparation of building blocks for glycopeptide synthesis by glycosylation of Fmoc amino acids having unprotected carboxyl groups. Tetrahedron. 1995;51(19):5643-5656.

4. Ren T, Liu D. Synthesis of targetable cationic amphiphiles. Tetrahedron letters. 1999;40(43):7621-7625.

5. Paquet A. Introduction of 9-fluorenylmethyloxycarbonyl, trichloroethoxycarbonyl, and benzyloxycarbonyl amine protecting groups into O-unprotected hydroxyamino acids using succinimidyl carbonates. Canadian Journal of Chemistry. 1982;60(8):976-980.

6. Barton D, Ferreira J, Jaszberenyi J, Hanessian S. Preparative Carbohydrate Chemistry. by S Hanessian, Marcel Dekker, Inc, New York. 1997:151.

7. Yu J, Westerlind U. Synthesis of a glycopeptide vaccine conjugate for induction of antibodies recognizing O-mannosyl glycopeptides. Chembiochem. 2014;15(7):939-945.

8. Keil S, Claus C, Dippold W, Kunz H. Towards the Development of Antitumor Vaccines: A Synthetic Conjugate of a Tumor-Associated MUC1 Glycopeptide Antigen and a Tetanus Toxin Epitope This work was supported by the Deutsche Forschungsgemeinschaft and by the Stiftung Rheinland-Pfalz fur Innovation. S.K. is grateful for a Kekule-Stipendium from the Fonds der Chemischen Industrie. Angew Chem Int Ed Engl. 2001;40(2):366-369.

9. Dziadek S, Hobel A, Schmitt E, Kunz H. A fully synthetic vaccine consisting of a tumor-associated glycopeptide antigen and a T-cell epitope for the induction of a highly specific humoral immune response. Angew Chem Int Ed Engl. 2005;44(46):7630-7635.

10. Ibatullin FM, Selivanov SI. Reaction of N-Fmoc aspartic anhydride with glycosylamines: a simple entry to N-glycosyl asparagines. Tetrahedron Letters. 2009;50(46):6351-6354.

11. Yu J, Schorlemer M, Gomez Toledo A, Pett C, Sihlbom C, Larson G, et al. Distinctive MS/MS Fragmentation Pathways of Glycopeptide-Generated Oxonium Ions Provide Evidence of the Glycan Structure. Chemistry. 2016;22(3):1114-1124.

12. Rising TW, Heidecke CD, Moir JW, Ling Z, Fairbanks AJ. Endohexosaminidase-catalysed glycosylation with oxazoline donors: fine tuning of catalytic efficiency and reversibility. Chemistry. 2008;14(21):6444-6464.

13. Li B, Zeng Y, Hauser S, Song H, Wang LX. Highly efficient endoglycosidase-catalyzed synthesis of glycopeptides using oligosaccharide oxazolines as donor substrates. J Am Chem Soc. 2005;127(27):9692-9693.

14. Liu Y, Childs RA, Palma AS, Campanero-Rhodes MA, Stoll MS, Chai W, et al. Neoglycolipid-based oligosaccharide microarray system: preparation of NGLs and their noncovalent immobilization on nitrocellulose-coated glass slides for microarray analyses. Methods Mol Biol. 2012;808:117-136.

15. Palma AS, Liu Y, Childs RA, Herbert C, Wang D, Chai W, et al. The human epithelial carcinoma antigen recognized by monoclonal antibody AE3 is expressed on a sulfoglycolipid in addition to neoplastic mucins. Biochem Biophys Res Commun. 2011;408(4):548-552.

16. Stoll MS & Feizi T. Software tools for storing, processing and displaying carbohydrate microarray data. Proceeding of the Beilstein Symposium on Glyco-Bioinformatics, 4-8 October, 2009, Potsdam, Germany. Kettner, C. 123-140. 2009. Beilstein Institute for the Advancement of Chemical Sciences, Frankfurt, Germany. http://www.beilstein-institut.de/en/publications/proceedings/glyco-2009
