# Supplementary material for: Protein O-Mannosylation in the Murine Brain: Occurrence of Mono-O-Mannosyl Glycans and Identification of New Substrates
Source: PLoS One. 2016 Nov 3;11(11):e0166119. doi: 10.1371/journal.pone.0166119 (PMC5094735; doi:10.1371/journal.pone.0166119)
Supplement: S2 File — (DOCX) [file pone.0166119.s013.docx]

**Supplementary Methods II**

**HPLC chromatograms of the individual compounds used for the glycoopeptide arrays**

# HPLC Chromatogram of synthetic *O*-mannosyl peptides

Compound 7, Sequence: NH_2_-Spacer-YAT*AVA, *=Gal-GlcNAc-Man.


Compound 8, Sequence: NH_2_-Spacer-PVPGKPT*VTIR, *=GlcNAc-Man.


Compound 9, Sequence: NH_2_-Spacer-RGAIIQT*PTLG, *=GlcNAc-Man.


Compound 10, Sequence: NH_2_-Spacer-YAT*AVA, *= GlcNAc-Man.


Compound 11, Sequence: NH_2_-Spacer-SQSLEET*ISPR, *= GlcNAc-Man.

Compound 12, Sequence: NH_2_-Spacer-SGPLDGGT*LLTIR, *= GlcNAc-Man.


Compound 13, Sequence: NH_2_-Spacer-NAPSGT*TVIHLNA, *= GlcNAc-Man.


Compound 14, Sequence: NH_2_-Spacer-NAPSGT*T*VIHLNA, *= GlcNAc-Man.


Compound 15, Sequence: NH_2_-Spacer-QGPQAGGT*T*LTIHG, *= GlcNAc-Man.

Compound 16, Sequence: NH_2_-Spacer-EPGGSYIT*T*VSATD, *= GlcNAc-Man.


Compound 17, Sequence: NH_2_-Spacer-PVPGKPT*VTIR, *=Man.


Compound 18, Sequence: NH_2_-Spacer-RGAIIQT*PTLG, *=Man.


Compound 19, Sequence: NH_2_-Spacer- SQSLEET*ISPR, *=Man.


Compound 20, Sequence: NH_2_-Spacer-SGPLDGGT*LLTIR, *=Man.


Compound 21, Sequence: NH_2_-Spacer-NAPSGT*TVIHLNA, *=Man.


Compound 22, Sequence: NH_2_-Spacer-NAPSGT*T*VIHLNA, *=Man.


Compound 23, Sequence: NH_2_-Spacer-QGPQAGGT*T*LTIHG, *=Man.


Compound 24, Sequence: NH_2_-Spacer-EPGGSYIT*T*VSATD, *=Man.


Compound 25, Sequence: NH_2_-Spacer-YATAVA.


# HPLC Chromatogram *N*-glycopeptides

Compound 35, Sequence: NH_2_-Spacer-N*LTALPPDLPK, *=GlcNAc.

Compound 36, Sequence: NH_2_-Spacer-LQNLTLPTN*ASIK, *=GlcNAc.

Compound 40, Sequence: NH_2_-Spacer-N*LTALPPDLPK, *= Man_3_GlcNAc_2_.

0.0

5.0

10.0

15.0

20.0

25.0

30.0

35.0

40.0

45.1

-200

250

500

750

1,000

Pep14030608+C4F7

UV_VIS_1

mAU

min

1 - 21.987

WVL:214 nm

Flow: 200 µl/min

%B: 10.0 %

40.0

90.0

5.0

%C: 0.0 %

0.0

5.0

10.0

15.0

20.0

25.0

30.0

35.0

40.0

45.1

-200

250

500

750

1,000

Pep14030613+C4F7

UV_VIS_1

mAU

min

1 - 24.417

WVL:214 nm

Flow: 200 µl/min

%B: 10.0 %

40.0

90.0

5.0

%C: 0.0 %

Compound 41, Sequence: NH_2_-Spacer-LQNLTLPTN*ASIK, *= Man_3_GlcNAc_._

0.0

5.0

10.0

15.0

20.0

25.0

30.0

35.0

40.0

45.1

-200

250

500

750

1,000

Pep14030613+C4F7

UV_VIS_1

mAU

min

1 - 24.417

WVL:214 nm

Flow: 200 µl/min

%B: 10.0 %

40.0

90.0

5.0

%C: 0.0 %
