# Supplementary material for: Protein O-Mannosylation in the Murine Brain: Occurrence of Mono-O-Mannosyl Glycans and Identification of New Substrates
Source: PLoS One. 2016 Nov 3;11(11):e0166119. doi: 10.1371/journal.pone.0166119 (PMC5094735; doi:10.1371/journal.pone.0166119)
Supplement: S1 Table — Monoclonal or polyclonal antibodies are indicated with m or p, respectively. Vendors and order numbers are provided, Antibody IDs from the Antibody Registry are indicated when available and used dilutions are given. (DOCX) [file pone.0166119.s015.docx]

| **Antibody/Lectin** | **Dilution** | **Antibody ID** | **P/M** | **Distributor** |
| --- | --- | --- | --- | --- |
| Rabbit α-Oman | 1:25 - 1:100 | - | M | This study |
| Rabbit T[α-1Man] | 1:25 | - | P | Lommel et al. 2013 [7] |
| Rabbit α-Laminin | 1:200 | AB_477163 | P | Sigma (L9393) |
| Rabbit α-Versican | 1:50 | AB_2241501 | P | Santa Cruz (sc-25831) |
| Rabbit α-POMT2 | 1:25 | - | P | Willer et al. 2002 [69] |
| Sheep α-Neurocan | 1:200 | AB_2149717 | P | R&D Systems (AF5800) |
| Mouse α-IIH6 | 1:50 | AB_309828 | M | Millipore (05-593) |
| Mouse α-PentaHis | 1:2500 | - | M | Qiagen (34660) |
| Goat α-Fox-3 (NeuN) | 1:200 | - | P | Santa Cruz (sc-246957) |
| Goat α-Plexin-B2 | 1:50 | AB_654244 | P | Santa Cruz (sc-34504) |
| Goat α-RPTPζ | 1:50 | AB_2173120 | P | Santa Cruz (sc-1110) |
| Goat α-Calbindin D28K | 1:50 | AB_634520 | P | Santa Cruz (sc-7691) |
| Goat α-Gephyrin | 1:50 | AB_640962 | P | Santa Cruz (sc-6411) |
| Goat α-Cre Recombinase | 1:50 | AB_1562201 | P | Santa Cruz (sc-83398) |
| Goat α-Glial fibrillary acidic protein (GFAP) Alexa Fluor 488 | 1:25 | AB_641023 | P | Santa Cruz (sc-6171) |
| Goat-α-rabbit Alexa Fluor 488 | 1:1000 | AB_143165 | P | Life Technologies (A-11008) |
| Donkey-α-rabbit Alexa Fluor 488 | 1:1000 | AB_2535792 | P | Life Technologies (A-21206) |
| Donkey-α-goat Alexa Fluor 568 | 1:1000 | AB_2534104 | P | Life Technologies (A-11057) |
| Donkey-α-rabbit Alexa Fluor 568 | 1:1000 | AB_2534017 | P | Life Technologies (A-10042) |
| Goat-α-mouse Alexa Fluor 488 | 1:1000 | AB_10566289 | P | Life Technologies (A-11001) |
| Donkey-α-sheep Cy3 | 1:100 | AB_2340728 | P | Jackson Immuno Research (713-165-147) |
| Rhodamine peanut agglutinin | 1:200 | - | - | Vector Labs (RL-1072) |
| Biotinylated Wisteria Floribunda Lectin (WFA) | 1:50 | - | - | Vector Labs (B-1355) |
| Avidin Alexa Fluor 488 | 1:100 | - | - | Life Technologies (A-21370) |
