# Supplementary material for: Protein O-Mannosylation in the Murine Brain: Occurrence of Mono-O-Mannosyl Glycans and Identification of New Substrates
Source: PLoS One. 2016 Nov 3;11(11):e0166119. doi: 10.1371/journal.pone.0166119 (PMC5094735; doi:10.1371/journal.pone.0166119)
Supplement: S2 Table — (DOCX) [file pone.0166119.s016.docx]

| **Pos.** | **Probe** | **Sequence** | **Fluorescence intensities at different RKU-1-3-5 dilutions** | | |
| --- | --- | --- | --- | --- | --- |
|  |  |  | **1:25** | **1:50** | **1:100** |
| 1 | Man2(α2) | Manα-2Man-DH | 3,613 | 1,436 | 632 |
| 2 | Man2(α3) | Manα-3Man-DH | 11,460 | 6,503 | 2,745 |
| 3 | Man2(α6) | Manα-6Man-DH | 22,211 | 18,039 | 12,389 |
| 4 | Man3(α3,α6) | Manα-6Man-DH  │  Manα-3 | 10,017 | 3,431 | 2,695 |
| 5 | Man5(α3,α6) | Manα-3  │ Manα-6Manα-6Man-DH  │  Manα-3 | 7,579 | 4,545 | 1,806 |
| 6 | Man1GN1 | Manβ-4GlcNAc-DH | 6,710 | 3,059 | 1,575 |
| 7 | Man2GN1 | Manα-3Manβ-4GlcNAc-DH | 12,530 | 7,732 | 4,819 |
| 8 | Man2aGN2 | Manα-6Manβ-4GlcNAcβ-4GlcNAc-DH | 662 | 171 | 128 |
| 9 | Man3GN2 | Manα-6  │  Manβ-4GlcNAcβ-4GlcNAc-DH  │ Manα-3 | 4,890 | 2,514 | 1,173 |
| 10 | Man3XylGN2 | Manα-6  │ Xylβ-2Manβ-4GlcNAcβ-4GlcNAc-DH  │  Manα-3 | 4,952 | 2,368 | 1,006 |
| 11 | Man3FGN2 | Manα-6 Fucα-6  │ │  Manβ-4GlcNAcβ-4GlcNAc-DH  │ Manα-3 | 4,283 | 1,566 | 1,102 |
| 12 | Man3FXylGN2 | Manα-6  │ Xylβ-2Manα-4GlcNAcβ-4GlcNAc-DH  │ │  Manα-3 Fucα-3 | 9,012 | 3,153 | 2,224 |
| 13 | Man4aGN2 | Manα-3Manα-6  │  Manβ-4GlcNAcβ-4GlcNAc-DH  │  Manα-3 | 10,017 | 7,127 | 2,908 |
| 14 | Man4bGN2 | Manα-6  │ Manα-3Manα-6  │  Manβ-4GlcNAcβ-4GlcNAc-DH | 3,046 | 1,482 | 558 |
| 15 | Man5GN2 | Manα-6  │ Manα-3Manα-6  │  Manβ-4GlcNAcβ-4GlcNAc-DH  │  Manα-3 | 2,578 | 1,779 | 666 |
| 16 | Man6GN2 | Manα-6  │ Manα-3Manα-6  │  Manβ-4GlcNAcβ-4GlcNAc-DH  │ Manα-2Manα-3 | 4,480 | 1,665 | 1,066 |
| 17 | Man7(D1)GN2 | Manα-6  │  Manα-3Manα-6  │  Manβ-4GlcNAcβ-4GlcNAc-DH  │ Manα-2Manα-2Manα-3 | 4,984 | 2,603 | 1,089 |
| 18 | Man7(D1)GN2-AO | Manα-6  │  Manα-3Manα-6  │  Manβ-4GlcNAcβ-4GlcNAc-AO  │ Manα-2Manα-2Manα-3 | 1,965 | 1,064 | 465 |
| 19 | Man7(D3)GN2 | Manα-2Manα-6  │  Manα-3Manα-6  │  Manβ-4GlcNAcβ-4GlcNAc-DH  │  Manα-2Manα-3 | 5,377 | 2,574 | 1,264 |
| 20 | Man8(D1D3)GN2 | Manα-2Manα-6  │  Manα-3Manα-6  │  Manβ-4GlcNAcβ-4GlcNAc-DH  │ Manα-2Manα-2Manα-3 | 6,941 | 2,515 | 1,812 |
| 21 | Man9GN2 | Manα-2Manα-6  │ Manα-2Manα-3Manα-6  │  Manβ-4GlcNAcβ-4GlcNAc-DH  │ Manα-2Manα-2Manα-3 | 2,569 | 1,292 | 545 |
| 22 | Man9GN2-AO | Manα-2Manα-6  │ Manα-2Manα-3Manα-6  │  Manβ-4GlcNAcβ-4GlcNAc-AO  │ Manα-2Manα-2Manα-3 | 1,519 | 729 | 335 |
| 23 | Glc1Man9GN2 | Manα-2Manα-6  │  Manα-6  │ │  Manα-2Manα-3 Manβ-4GlcNAcβ-4GlcNAc-DH  │ Glcα-3Manα-2Manα-2Manα-3 | 1,160 | 420 | 352 |
| 24 | Glc1Man9GN2-AO | Manα-2Manα-6  │  Manα-6  │ │  Manα-2Manα-3 Manβ-4GlcNAcβ-4GlcNAc-AO  │ Glcα-3Manα-2Manα-2Manα-3 | 1,092 | 389 | 245 |
| 25 | Glc2Man7(D1)GN1-AO | Manα-6  │  Manα-3Manα-6  │  Manβ-4GlcNAc-AO  │ Glcα-3Glcα-3Manα-2Manα-2Manα-3 | 1,879 | 753 | 340 |
| 26 | Glc2Man9GN2-AO | Manα-2Manα-6  │  Manα-6  │ │  Manα-2Manα-3 Manβ-4GlcNAcβ-4GlcNAc-AO  │ Glcα-3Glcα-3Manα-2Manα-2Manα-3 | 627 | 228 | 80 |
| 27 | Glc3Man7(D1)GN1-AO | Manα-6  │  Manα-3Manα-6  │  Manβ-4GlcNAc-AO  │ Glcα-2Glcα-3Glcα-3Manα-2Manα-2Manα-3 | 1,353 | 552 | 251 |
| 28 | Glc3Man9GN2-AO | Manα-2Manα-6  │  Manα-6  │ │  Manα-2Manα-3 Manβ-4GlcNAc-AO  │ Glcα-2Glcα-3Glcα-3Manα-2Manα-2Manα-3 | 1,094 | 389 | 214 |
| 29 | N1 | Galβ-4GlcNAcβ-2Manα-6 Fucα-6  │ │  Manβ-4GlcNAcβ-4GlcNAc-DH  │  Manα-3 | 279 | 48 | 16 |
| 30 | N2 | Manα-6  │  Manβ-4GlcNAcβ-4GlcNAc-DH  │ Galβ-4GlcNAcβ-2Manα-3 | 35 | - | - |
| 31 | N4 | Galβ-4GlcNAcβ-2Manα-6  │  Manβ-4GlcNAcβ-4GlcNAc-DH  │  Manα-3 | 1,869 | 720 | 327 |
| 32 | N3 | GlcNAcβ-2Manα-6 Fucα-6  │ │ Galβ-4 Manβ-4GlcNAcβ-4GlcNAc-DH  │  GlcNAcβ-2Manα-3 | - | - | - |
| 33 | NGA2 | GlcNAcβ-2Manα-6  │  Manβ-4GlcNAcβ-4GlcNAc-DH  │ GlcNAcβ-2Manα-3 | 313 | 51 | 2 |
| 34 | NGA2F | GlcNAcβ-2Manα-6 Fucα-6  │ │  Manβ-4GlcNAcβ-4GlcNAc-DH  │ GlcNAcβ-2Manα-3 | - | 1 | - |
| 35 | NGA2B | GlcNAcβ-2Manα-6  │  GlcNAcβ-4Manβ-4GlcNAcβ-4GlcNAc-DH  │ GlcNAcβ-2Manα-3 | 15 | 63 | 30 |
| 36 | NGA3B | GlcNAcβ-2Manα-6  │  GlcNAcβ-4Manβ-4GlcNAcβ-4GlcNAc-DH  │ GlcNAcβ-4Manα-3  │  GlcNAcβ-2 | 70 | 110 | - |
| 37 | NGA4 | GlcNAcβ-6  │ GlcNAcβ-2Manα-6  │  Manβ-4GlcNAcβ-4GlcNAc-DH  │ GlcNAcβ-2Manα-3  │  GlcNAcβ-4 | - | - | - |
| 38 | NGA5B | GlcNAcβ-2  │ GlcNAcβ-4Manα-6  │ │  GlcNAcβ-6 │  │  GlcNAcβ-4Manβ-4GlcNAcβ-4GlcNAc-DH  │ GlcNAcβ-4Manα-3  │  GlcNAcβ-2 | - | - | - |
| 39 | GNMan5BGN2 | Manα-6  │  Manα-3Manα-6  │  GlcNAcβ-4Manβ-4GlcNAcβ-4GlcNAc-DH  │ GlcNAcβ-2Manα-3 | 134 | 32 | 43 |
| 40 | NA2 | Galβ-4GlcNAcβ-2Manα-6  │  Manβ-4GlcNAcβ-4GlcNAc-DH  │ Galβ-4GlcNAcβ-2Manα-3 | 17 | 7 | - |
| 41 | NA2F | Galβ-4GlcNAcβ-2Manα-6 Fucα-6  │ │  Manβ-4GlcNAcβ-4GlcNAc-DH  │ Galβ-4GlcNAcβ-2Manα-3 | 61 | 25 | - |
| 42 | NA2F-AO | Galβ-4GlcNAcβ-2Manα-6 Fucα-6  │ │  Manβ-4GlcNAcβ-4GlcNAc-AO  │ Galβ-4GlcNAcβ-2Manα-3 | 230 | 125 | 37 |
| 43 | NA2FB | Galβ-4GlcNAcβ-2Manα-6 Fucα-6  │ │  GlcNAcβ-4Manβ-4GlcNAcβ-4GlcNAc-DH  │ Galβ-4GlcNAcβ-2Manα-3 | - | - | - |
| 44 | NA3-Lex | Galβ-4GlcNAcβ-2Manα-6  │ Fucα-3 Manβ-4GlcNAcβ-4GlcNAc-DH  │  Galβ-4GlcNAcβ-4Manα-3  │  Galβ-4GlcNAcβ-2 | 46 | 28 | 27 |
| 45 | NA4 | Galβ-4GlcNAcβ-6  │ Galβ-4GlcNAcβ-2Manα-6  │  Manβ-4GlcNAcβ-4GlcNAc-DH  │ Galβ-4GlcNAcβ-4Manα-3  │   Galβ-4GlcNAcβ-2 | 161 | 40 | - |
| 46 | A2F(2-3) | NeuAcα-3Galβ-4GlcNAcβ-2Manα-6 Fucα-6  │ │  Manβ-4GlcNAcβ-4GlcNAc-DH  │ NeuAcα-3Galβ-4GlcNAcβ-2Manα-3 | - | - | - |
| 47 | A2(2-6) | NeuAcα-6Galβ-4GlcNAcβ-2Manα-6  │  Manβ-4GlcNAcβ-4GlcNAc-DH  │ NeuAcα-6Galβ-4GlcNAcβ-2Manα-3 | - | - | - |
| 48 | Man-Ser | Manα-Ser-DH | 20,599 | 21,782 | 12,612 |
| 49 | Man-Ser-Succ | Manα-Ser-Succ-DH | 17,050 | 12,350 | 6,657 |
| 50 | Man-Thr | Manα-Thr-DH | 8,013 | 4,465 | 2,494 |
| 51 | GalNAc-Ser | GalNAcα-Ser-DH | 344 | 113 | 17 |
| 52 | Notch-1 | Fucα-Thr-DH | 496 | 185 | 53 |

^a^ Pos, Probe position in the screening microarray.

^b^ Unless otherwise specified the NGLs are prepared from reducing oligosaccharides by reductive amination with the amino lipid, 1,2-dihexadecyl-*sn*-glycero-3-phosphoethanolamine (DHPE); AO, NGLs prepared from reducing oligosaccharides by oxime ligation with an aminooxy (AO) functionalized DHPE (Liu et al., Chem. Biol. 14, 847–859, 2007).

^c^ Average fluorescence intensity of the duplicate spots.

-, signal less than 1.

Abbreviations:

Gal: Galactose

GalNAc: N-Acetylgalactosamine

Glc: Glucose

GlcNAc: N-Acetylglucosamine

Man: Mannose

Fuc: Fucose

NeuAc: N-Acetylneuraminic acid

Succ: Succinic anhydride

Xyl: Xylose
