# Supplementary material for: Protein O-Mannosylation in the Murine Brain: Occurrence of Mono-O-Mannosyl Glycans and Identification of New Substrates
Source: PLoS One. 2016 Nov 3;11(11):e0166119. doi: 10.1371/journal.pone.0166119 (PMC5094735; doi:10.1371/journal.pone.0166119)
Supplement: S3 Table — Glycan positions are indicated in the peptide sequence by an asterisk (*). Spacers are indicated in the peptide sequence (Sp). (DOCX) [file pone.0166119.s017.docx]

| **Comp Nr** | **Origin** | **Sequence** | **Glycan** |
| --- | --- | --- | --- |
| 28 | α-Dystroglycan | *Sp*PVPGKPT*VTIR | Galβ1-4GlcNAcβ1-2Manα |
| 29 | α-Dystroglycan | *Sp*RGAIIQT*PTLG |  |
| 30 | antigen sequence | *Sp*GT*G |  |
| 7 | antigen sequence | *Sp*YAT*AVA |  |
| 8 | α-Dystroglycan | *Sp*PVPGKPT*VTIR | GlcNAcβ1-2Manα |
| 9 | α-Dystroglycan | *Sp*RGAIIQT*PTLG |  |
| 31 | antigen sequence | *Sp*GT*G |  |
| 10 | antigen sequence | *Sp*YAT*AVA |  |
| 11 | UPF0606 protein KIAA1549 | *Sp*SQSLEET*ISPR |  |
| 12 | Plexin-D1 | *Sp*SGPLDGGT*LLTIR |  |
| 13 | Protocadherin Fat 4 | *Sp*NAPSGT*TVIHLNA |  |
| 14 | Protocadherin Fat 4 | *Sp*NAPSGT*T*VIHLNA | GlcNAcβ1-2Manα x2 |
| 15 | Plexin-B2 | *Sp*QGPQAGGT*T*LTIHG |  |
| 16 | Protocadherin Fat 4 | *Sp*EPGGSYIT*T*VSATD |  |
| 26 | antigen sequence | YAT*AV | Manα |
| 17 | α-Dystroglycan | *Sp*PVPGKPT*VTIR |  |
| 18 | α-Dystroglycan | *Sp*RGAIIQT*PTLG |  |
| 19 | UPF0606 protein KIAA1549 | *Sp*SQSLEET*ISPR |  |
| 20 | Plexin-D1 | *Sp*SGPLDGGT*LLTIR |  |
| 21 | Protocadherin Fat 4 | *Sp*NAPSGT*TVIHLNA |  |
| 22 | Protocadherin Fat 4 | *Sp*NAPSGT*T*VIHLNA | Manα x2 |
| 23 | Plexin-B2 | *Sp*QGPQAGGT*T*LTIHG |  |
| 24 | Protocadherin Fat 4 | *Sp*EPGGSYIT*T*VSATD |  |
| 25 | antigen sequence | *Sp*YATAVA | non-glycan |
| 27 | antigen sequence | YATAV |  |
