# Supplementary material for: Protein O-Mannosylation in the Murine Brain: Occurrence of Mono-O-Mannosyl Glycans and Identification of New Substrates
Source: PLoS One. 2016 Nov 3;11(11):e0166119. doi: 10.1371/journal.pone.0166119 (PMC5094735; doi:10.1371/journal.pone.0166119)
Supplement: S4 Table — Glycan positions are indicated in the peptide sequence by an asterisk (*). Spacers are indicated in the peptide sequence (Sp). (DOCX) [file pone.0166119.s018.docx]

| **Comp Nr** | **Origin** | **Sequence** | **Glycans** |
| --- | --- | --- | --- |
| 38 | Thrombospondin-1: | *Sp*VVN*STTGPGEHLR | Man_3_-GlcNAc_2_ |
| 39 | Antithrombin III: | *Sp*WVSN*KTEGR |  |
| 40 | Platelet glycoprotein 1b alpha: | *Sp*N*LTALPPDLPK |  |
| 41 | Multimerin 1: | *Sp*LQNLTLPTN*ASIK |  |
